# Questing for Integrin Targeting Theranostics for Cancer Cell-Selective Molecules

**DOI:** 10.1021/acsomega.6c00193

**Published:** 2026-05-05

**Authors:** Valentina Giraldi, Tania Pecoraro, Andrea Maurizio, Alessia Milana, Luisa De Cola, Monica Baiula, Daria Giacomini

**Affiliations:** † Department of Chemistry “Giacomo Ciamician”, 9296 University of Bologna, Via Piero Gobetti, 85, 40129 Bologna, Italy; ‡ Department of Pharmaceutical Sciences (DiSFarm), University of Milan, Via Luigi Mangiagalli, 25, 20133 Milano, Italy; § Department of Pharmacy and Biotechnology, University of Bologna, Via Irnerio, 48, 40126 Bologna, Italy

## Abstract

Small molecules may play a significant role as theranostic agents for the diagnosis and treatment of some severe disorders, such as cancer. A theranostic agent must have a therapeutic moiety, an imaging group for diagnostics, and a targeting portion for specific cell recognition. In this study, we synthesized two new molecules, **D** and **E**, as potential theranostic agents, consisting of a β-lactam portion acting as a selective α_4_β_1_ integrin agonist, a fluorescent imaging probe for diagnostics purposes, and a cytotoxic portion for anticancer activity. Compound **D** incorporates a 1,8-naphthylimide fluorophore for imaging and the 5-FU cytotoxic unit, whereas compound **E** features BODIPY moiety serving both as a photosensitizer and as a cytotoxic agent. Both compounds were evaluated for their photophysical properties. The binding assays for α_4_β_1_ integrin revealed that only **E** had a good affinity (*K*_D_ 68.2 ± 1.0 μM). In adhesion assays with Jurkat E6.1, K562, HT-29, and HEK-293 cells, **E** retained the selective agonist activity for α_4_β_1_ integrin (EC_50_ 0.92 ± 0.14 μM) as the parental β-lactam ligand **A**. Upon photosensitization, compound **E** induced a significant concentration-dependent reduction in cell viability across all tested cell lines, regardless of integrin expression. Internalization experiments indicated a nonselective cellular uptake of compound **E**, likely due to its high lipophilicity, which may outweigh the contribution of structural elements responsible for specific integrin recognition.

## Graphical abstract

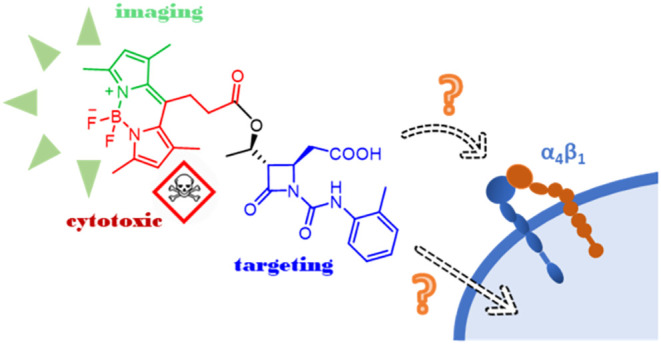

## Introduction

Theranostic is a field that encompasses diagnostic imaging and therapeutic treatment.^1^ This integration emphasizes the interplay between diagnosis, drug delivery, and treatment response monitoring, and aims to minimize adverse effects, thus facilitating a targeted and personalized therapeutic approach.^2,3^ Theranostic agents (theranostics) offer innovative strategies for the diagnosis and treatment of disorders such as cancer, infections, cardiovascular diseases, neurological and neurodegenerative disorders, and inflammatory disorders.^4–6^ Treatment approaches with theranostics are based on several systems: in nuclear medicine with constructs that include radioactive nuclides,^4,7^ in nanomedicine using nanoparticles or quantum dots,^8^ with macromolecular systems constituted by polymers or gels conjugated with therapeutic drugs,^9,10^ and with small drug conjugates.^11^ Functional nanomaterials and radioligands could offer significant potential as theranostics, but they showed several critical issues,^12^ including, among others, potential toxicity and suboptimal biodistribution with accumulation in organs. Even polymeric drug conjugates or organic nanocarriers such as liposomes have some drawbacks in triggering immune responses, thus resulting in allergic reactions or immunotoxicity.^12^ Considering these disadvantages, small molecules and their conjugates come into action as lead players as theranostics.^11^

A small molecule as a theranostic agent must contain a therapeutic subunit as the drug, the imaging moiety appointed to the diagnostics, and the biological target recognition portion to reduce off-target and side effects. In particular, it has been recognized how molecular interactions with receptors that control cell-to-cell communications may mediate effective biological targets.^13^

Integrins are transmembrane receptors that function as cell adhesion and signaling proteins. They are found on the surface of various cell subsets and have been recognized as valuable drug targets.^14^ These receptors are overexpressed in many types of cancer cells, mediate several hallmarks of cancer and could represent an effective biological target for theranostic agents.^15–21^ In particular, α_4_β_1_ integrin, expressed on several types of cancer cells, is involved in metastasis, mediating transendothelial migration, and contributes to the development of drug resistance.^17^ Moreover, α_4_β_1_ is expressed on hematopoietic stem cells and can be considered a biomarker of malignant transformation.^22^ Furthermore, integrins are well-established pharmacological targets for several significant pathologies, including neurodegenerative diseases such as Alzheimer’s disease, multiple sclerosis, and stroke.^23,24^

Concerning fluorophores, 1,8-Naphthalimide-derivatives and boron-dipyrromethenes (BODIPYs) are emerging intermediates in supramolecular chemistry, biomedicine, and materials science owing to their spectroscopic and electronic properties.^25^ As integrin-targeting drug delivery systems containing the naphthalimide fluorophore, small conjugates targeting α_v_β_3_ integrin were developed.^26,27^ They have an RGD-cyclopeptide as the integrin targeting unit connected by a disulfide linker to 1,8-naphthalimide and camptothecin. In these systems, the delivery of the drug can be directly monitored through the change in the emission properties of the naphthalimide core triggered by a reductive linker cleavage in cancer cells.^28^

Recently, near-infrared fluorescent BODIPY dyes were conjugated with RGD cyclopeptides (cRGD) for integrin recognition.^29,30^ BODIPY dyes are activatable photosensitizers for photodynamic therapy because, on light exposure, allowed an in situ production of highly reactive oxygen species (ROS) as cytotoxic agents.^29^ Imaging experiments indicated that the BODIPY-cRGD conjugate could preferentially enter cancer cells overexpressing α_v_β_3_ integrin, and the RGD peptide not only enabled the dye to detect α_v_β_3_ integrin high expression but also improved the solubility properties of the BODIPY derivative.^30^

Our previous studies provided a series of novel molecules designed to selectively target different integrins, mainly RGD-binding or leukocyte subtypes, which are capable of modulating integrin-mediated cellular processes. Some ligands behave as agonists, promoting cell adhesion and intracellular signaling, while others, acting as integrin antagonists, are able to inhibit integrin-dependent cellular functions. In addition, integrin agonists can promote integrin trafficking, can be internalized within cells, and thus represent good candidates as targeting units for delivery systems and theranostics.^31,32^ In a previous project, we realized targeted conjugated molecules for selective delivery of the cytotoxic agent 5-fluorouracil (5-FU) to cancer cells via specific integrin binding.^31^ To promote an active integrin-targeting action, we inserted a β-lactam core based on the ligand **A** as a model agonist compound (Figure 1). As previously demonstrated, the *o*-tolyl-urea linked to the β-lactam nitrogen atom and the carboxylic acid on the side-chain of the ring are crucial for integrin recognition.^33,34^ The 5-FU conjugate molecule **B** (Figure 1) emerged as a selective cytotoxic agent as it showed a differential pro-apoptotic concentration-dependent effect against K562 cells overexpressing α_5_β_1_ integrin, and not against Jurkat E6.1 cells overexpressing α_4_β_1_.^31^ This selectivity could be ascribed to the different integrin expression patterns in cancer cells and the antithetical integrin activity of **B** as both an agonist toward α_5_β_1_ integrin and an antagonist for α_4_β_1_. The insertion of a fluorescent moiety in the model compound **A**, as in ligand **C**, allowed us to check the intracellular uptake of the ligand by flow cytometry.^31^ The fluorescent-FITC-conjugated **C**, as an integrin agonist for both α_4_β_1_ and α_5_β_1_, was internalized in a concentration-dependent manner in both Jurkat E6.1 and K562 cells, expressing the targeted integrins. Supported by these results and to match (i) a selective cell internalization mediated by a β-lactam-based ligand for integrin recognition, (ii) an imaging probe by fluorescent residues, and (iii) a cytotoxic portion, we designed and synthesized two new conjugate molecules, **D** and **E**, as potential theranostic agents targeting cancer cells expressing α_4_β_1_ integrin.

**Figure 1:**
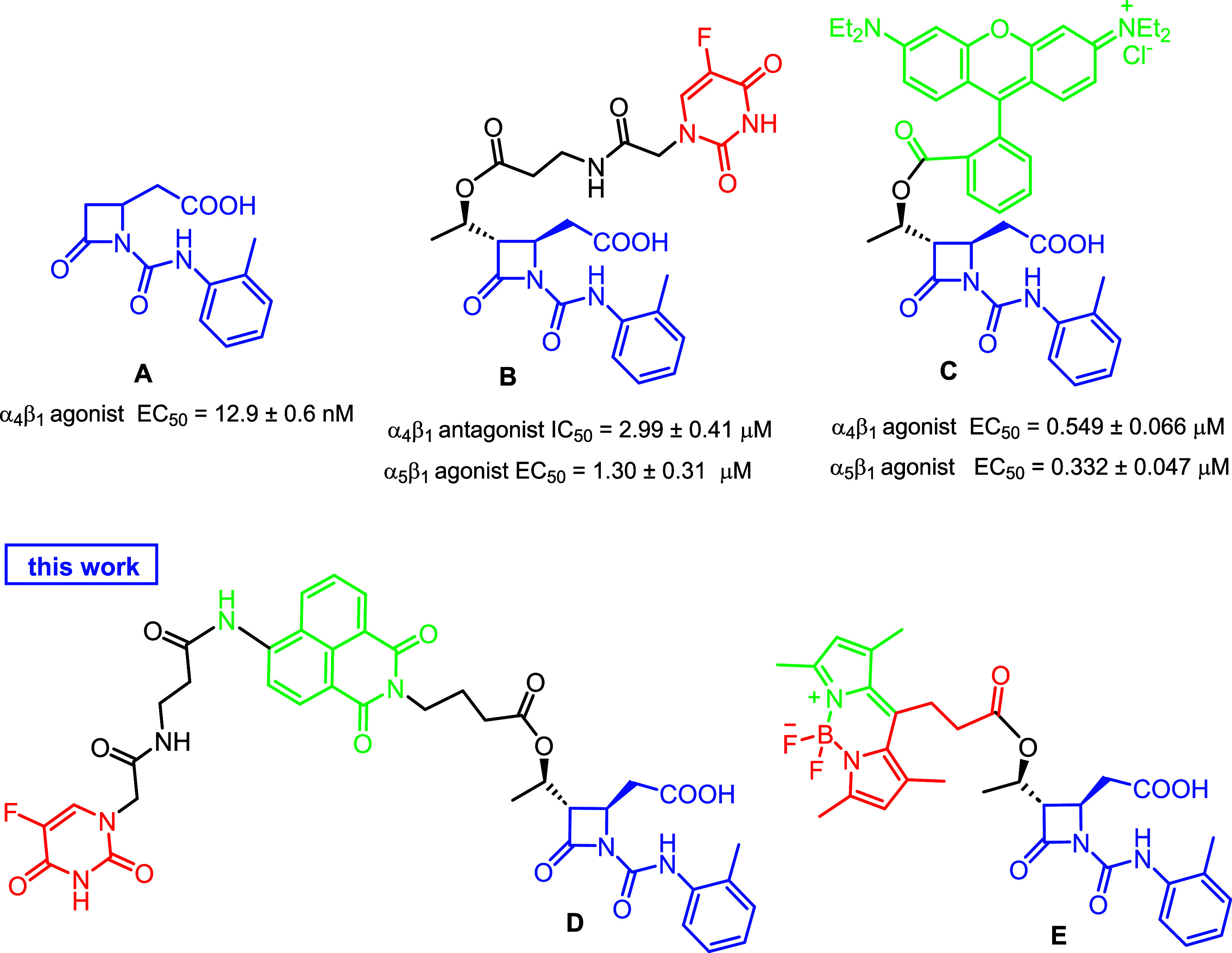
β-Lactam compounds previously reported (A–C) and selected for the design of the new molecules (D, E). Blue is the integrin-recognition core, red is the cytotoxic moiety, and green is the fluorescent portion for imaging visualization.

Compound **D** is a direct evolution of the previously developed ligands,^31^ and it could be considered an all-in-one system with the three distinct functionalities of a theranostic agent into one integrated molecule: the ureidic β-lactam portion for integrin recognition, 1,8-naphthylimide for imaging, and the cytotoxic 5-FU. Compound **E**, on the contrary, could be considered a one-for-all system with the β-lactam portion to target integrins and the BODIPY as photosensitizer and cytotoxic agent.^35,36^ Therefore, this study aimed to characterize the affinity of both theranostic conjugates **D** and **E**, and their respective negative control molecules, to α_4_β_1_ integrin, their selectivity toward other integrin heterodimers, and their cytotoxic effects in various cancer cell models expressing specific integrins.

## Results and Discussion

### Chemistry

The retro-synthetic analysis of compound **D** is outlined in Figure 2. The convergent synthesis made use of ester and amide bonds as cleavable linkages by cellular enzymes, to build-in the specific portions of the theranostic. The β-lactam portion **3**, the integrin targeting unit, was coupled with the intermediate **10**, composed in turn by the cytotoxic 5-FU portion **6** and by 1,8-naphthylimide **9** for imaging. Compounds **3**, **6**, and **9** could be synthesized from commercially available reagents **SM1**, **SM2**, and **SM3**, respectively.

**Figure 2:**
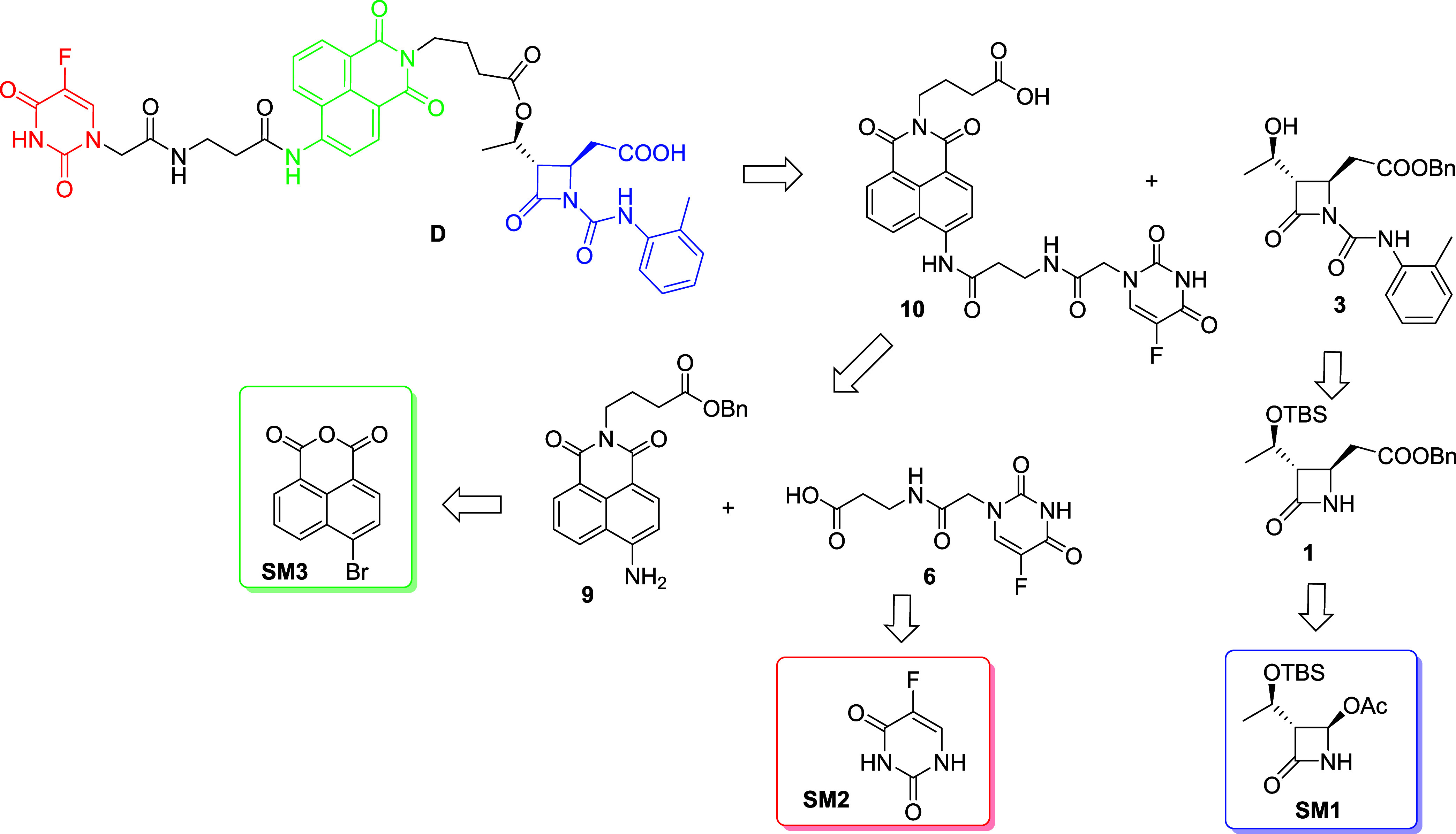
Retrosynthetic route for compound **D**.

The β-lactam **1** was obtained from the commercially available (3*R*,4*R*)-3-[(*R*)-1-*tert*-butyldimethylsiloxyethyl]-4-acetoxy-2-azetidinone **SM1** using an organozinc reagent as already reported (Scheme 1).^33^ Acylation on the nitrogen with o-tolyl isocyanate and triethylamine (TEA) gave the intermediate **2**, which upon deprotection by BF_3_·Et_2_O afforded **3** in good yields. The synthesis of the cytotoxic portion started from 5-Fluoro uracil **SM2** ^37^ that was regioselectivity functionalized with chloroacetic acid and KOH in water yielding **4** in moderate yields (Scheme 1). Compound **4** was then coupled to the commercially available β-alanine benzyl ester as PTSA salt, previously desalted with TEA, in the presence of coupling agents HOBt and EDCl. The workup procedure was optimized and **5** was obtained in good purity and yield. Deprotection of the benzyl ester by fast hydrogenolysis gave the cytotoxic intermediate **6**. This step was quite sensitive to a prolonged reaction time, which could give a defluorination reaction of the intermediate.^38^ Starting from the commercial 4-bromo-1,8-naphthalic anhydride **SM3**, a nucleophilic substitution reaction was carried out with the γ-aminobutyric acid (GABA) benzyl ester as PTSA salt desalted in situ with TEA, obtaining compound **7** (Scheme 1). Then, an aromatic nucleophilic substitution of the bromide with sodium azide easily gave the intermediate **8**. Finally, azide reduction with NaHS afforded the imaging portion **9**. Formation of the amide bond between amine **9** and acid **6** was the most critical point of the whole synthesis owing to the poor nucleophilicity of the naphthylamine. To improve this step, different coupling agents and conditions were studied (see Supporting Information Table S1),^39^ but carbodiimides or propane phosphonic acid anhydride (T3P)^40^ gave compound **10** in very low yields. Activation of the carboxylic acid **6** via the corresponding acyl chloride was then investigated (Supporting Information Scheme S1). The one-pot procedure using SOCl_2_ in DCM or oxalyl chloride in THF followed by the addition of pyridine and a solution of amine **9** in DMF or THF gave **10** after filtration as a yellow solid in yields up to 63%. Compound **10** was deprotected by hydrogenolysis in DMF to give **11**, whose esterification with the β-lactam alcohol **3** with excess EDCl and DMAP in a 2:1 mixture of anhydrous DCM and DMF to give **12**. Purification of the crude by liquid chromatography was a difficult task due to the low solubility of the compound; thus, isolation of **12** was achieved through acid–base work up and trituration in acetonitrile. The final deprotection by hydrogenolysis of **12** gave the final compound D in good yields and purity (see Supporting Information for experimental procedures).

**Scheme 1:**
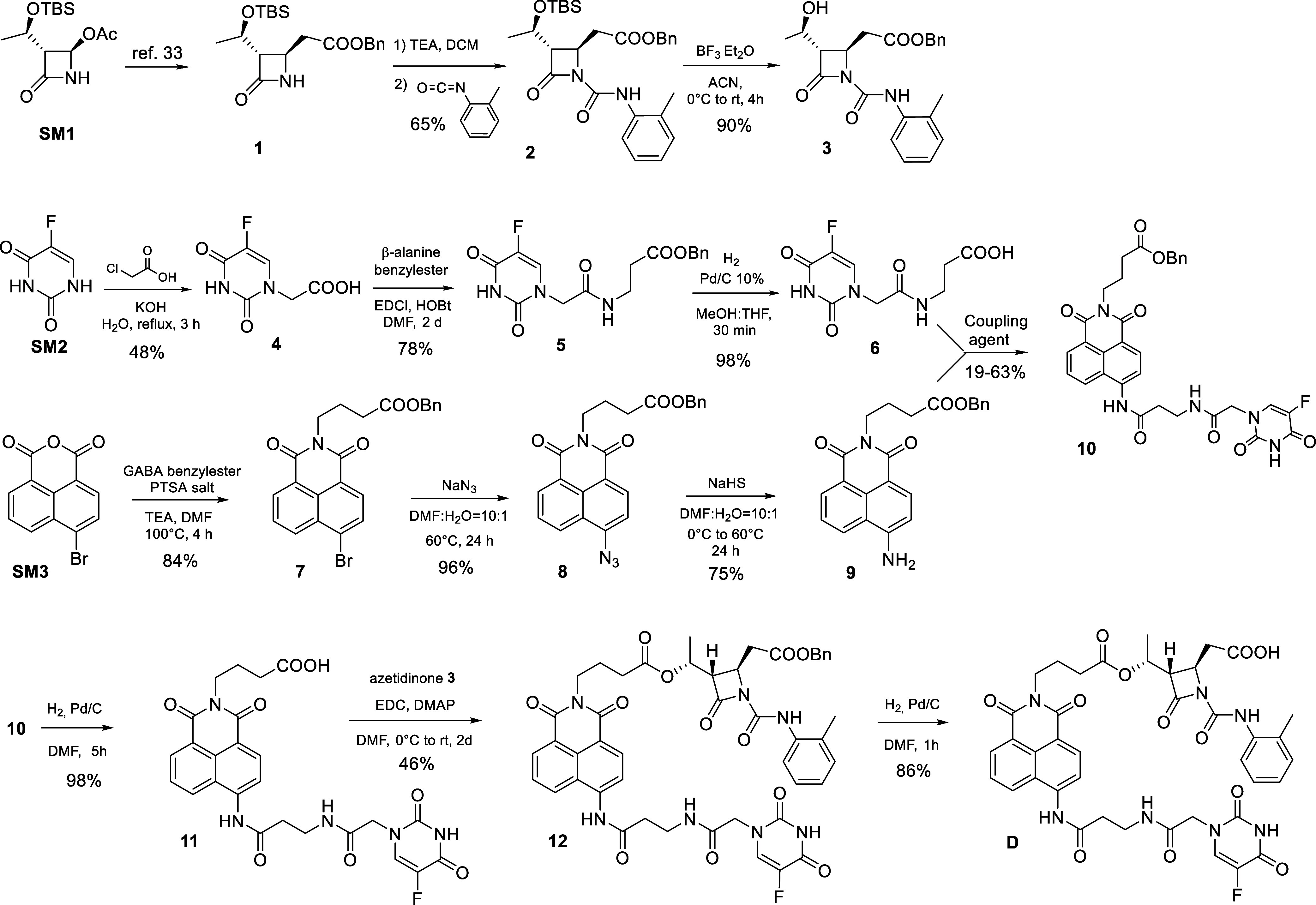
Synthesis of Intermediates **3**-**10** and **D**

**Table 1:**
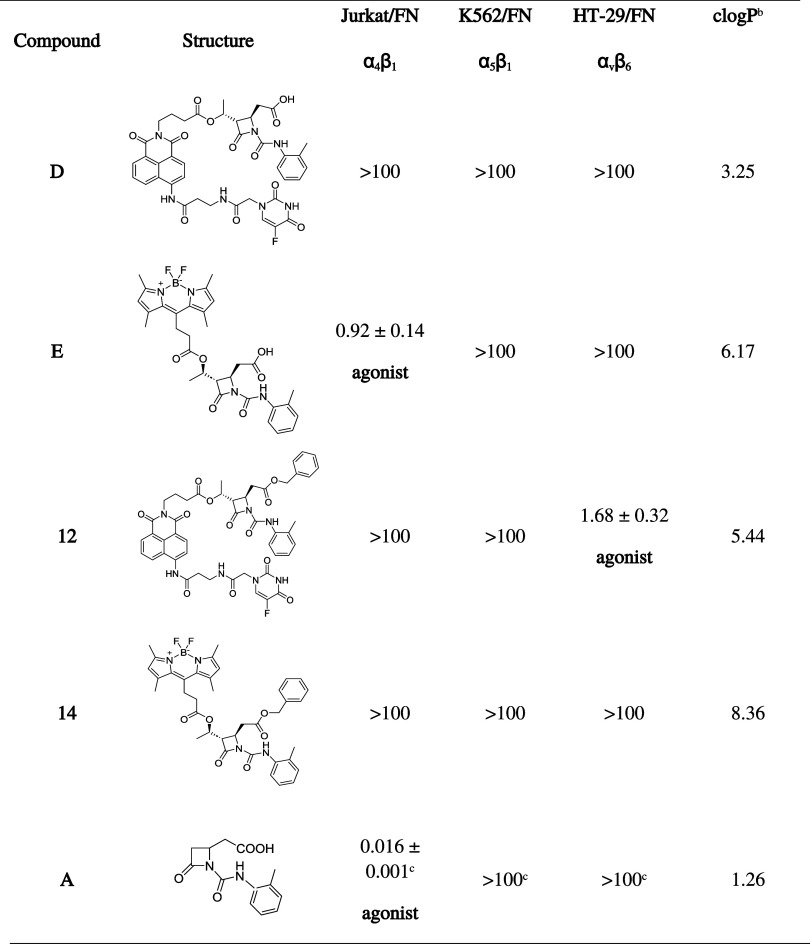
Effects of Compounds **D**, **E**, **12**, **14**, and **A** on Integrin-Mediated Cell Adhesion Performed on Jurkat E6.1 for α_4_β_1_, K562 for α_5_β_1_, and HT-29 Cells for α_v_β_6_ Integrin^a^

a Values represent the mean ± SD of three independent experiments performed in quadruplicate, and are shown as EC_50_ (μM).

b Calculated logP (CLogP) values were obtained with ChemDraw 21.0 program (specific algorithms for calculating logP from fragment-based methods were developed by the Medicinal Chemistry Project of CambridgeSoft and BioByte).

c Data previously reported.^46^

Synthesis of compound **E** started from the intermediate **13** prepared according to the literature with the commercially available 2,4-dimethylpyrrole and succinic anhydride in the presence of TEA and BF_3_.Et_2_O (Scheme 2).^41^ The low yields of this reaction are due to the formation of several byproducts and to a difficult purification of the target compound, which often required two purification runs by liquid chromatography. Intermediate **13** was then coupled with azetidinone **3** with a Steglich esterification by carbodiimide EDC to give **14**, which underwent the benzyl ester deprotection by hydrogenolysis as final step to compound **E**. This could be purified by flash chromatography to obtain purity grades above 95% for biological and photophysical studies.

**Scheme 2:**
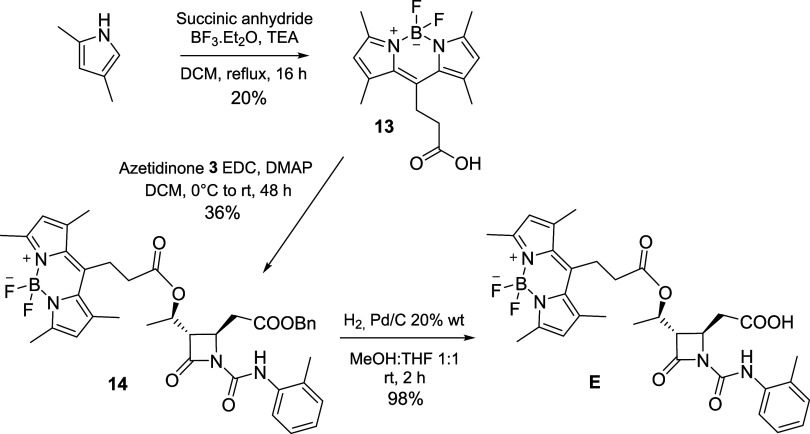
Synthesis of Compound **E**

### Photophysical Characterization

The photophysical properties of compounds **D** and **E** were evaluated. Absorption, emission, and excitation spectra were recorded to find the maximum absorption and emission λ, lifetimes of the excited states, and determination of quantum yields. All the results are shown in Figure 3. These studies were conducted at 10 μM concentration in PBS supplemented with 1% DMSO to allow complete solubilization.

**Figure 3:**
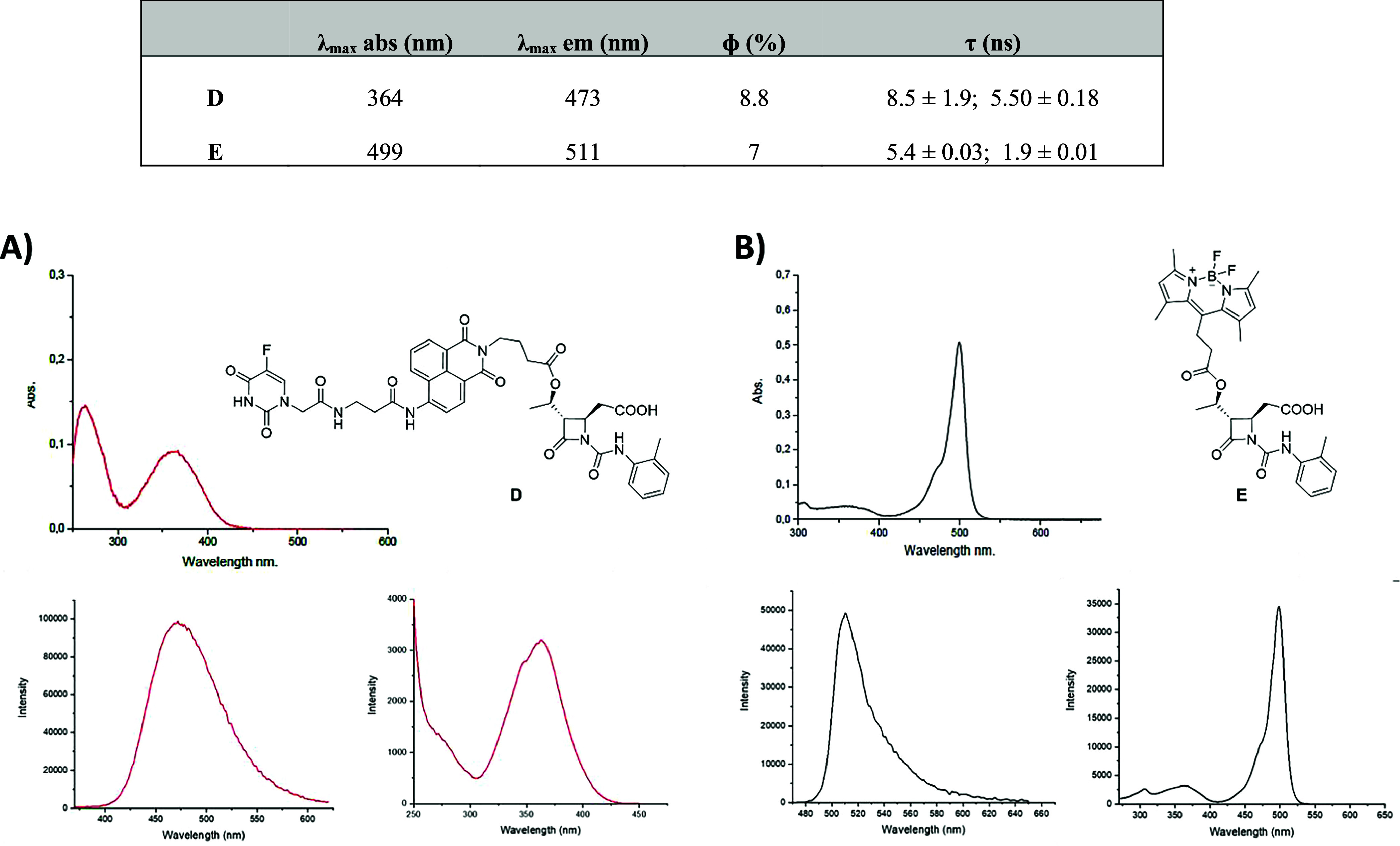
Photophysical data of compounds **D** and **E**. (A) Absorption (top), emission (bottom left), and excitation (bottom right) spectra of compound **D**. Emission spectrum was recorded after excitation at 320 nm; excitation spectrum recorded setting the emission at 470 nm. (B) Absorption (top), emission (bottom left), and excitation (bottom right) spectra of compound **E**. Emission spectrum was recorded after excitation at 450 nm; excitation spectrum recorded setting the emission at 505 nm.

Compounds **D** and **E** exhibit good fluorescence quantum yields (see data in Figure 3). This is a significant advantage, as a high quantum yield implies that these compounds are efficient light emitters, making them highly promising for applications like bioimaging, biosensors, and diagnostic tools where robust fluorescent signals are required. Furthermore, the absorption and emission properties of compounds **D** and **E** are in good agreement with those of analogous molecules reported in scientific literature.^26,42^ These results not only validate our experimental data but also confirm that compounds **D** and **E** behave as expected for their chemical class and support their potential utility in theranostic systems.

### Pharmacological Characterization of the New Theranostics

To assess the binding affinity to α_4_β_1_ integrin of conjugated compounds **D**, **E**, and **12** and **14** as parent esters, we developed a saturation binding experiment using LDV as a cold ligand and Jurkat E6.1 cells, endogenously expressing α_4_β_1_. Specific binding data from saturation binding assays were best fitted to a one-affinity site model, and *K*_D_ values are shown in Figure 4. Compound **A** is considered the parental reference integrin ligand.^33^ Compounds **12** and **14** were tested as tentative negative controls because, lacking the free carboxylic acid group necessary for binding at the metal ion dependent site (MIDAS) of integrins, in a previous study, benzylic ester derivatives were shown to be inactive against α_4_β_1_ integrin.^33^ As shown in Figure 4, the binding of compound **E** to α_4_β_1_ integrin expressed on Jurkat 6.1 cells was both concentration-dependent and saturable. However, the binding affinity of **E** significantly decreased compared to that of the parental compound **A**, likely due to the substantial steric bulk of the theranostic compound. Unexpectedly, compound **D** exhibited no affinity toward α_4_β_1_ integrin. Regarding the two control compounds, **12** was unable to bind to α_4_β_1_ integrin, but compound **14** demonstrated a binding affinity comparable to that of **E**.

**Figure 4:**
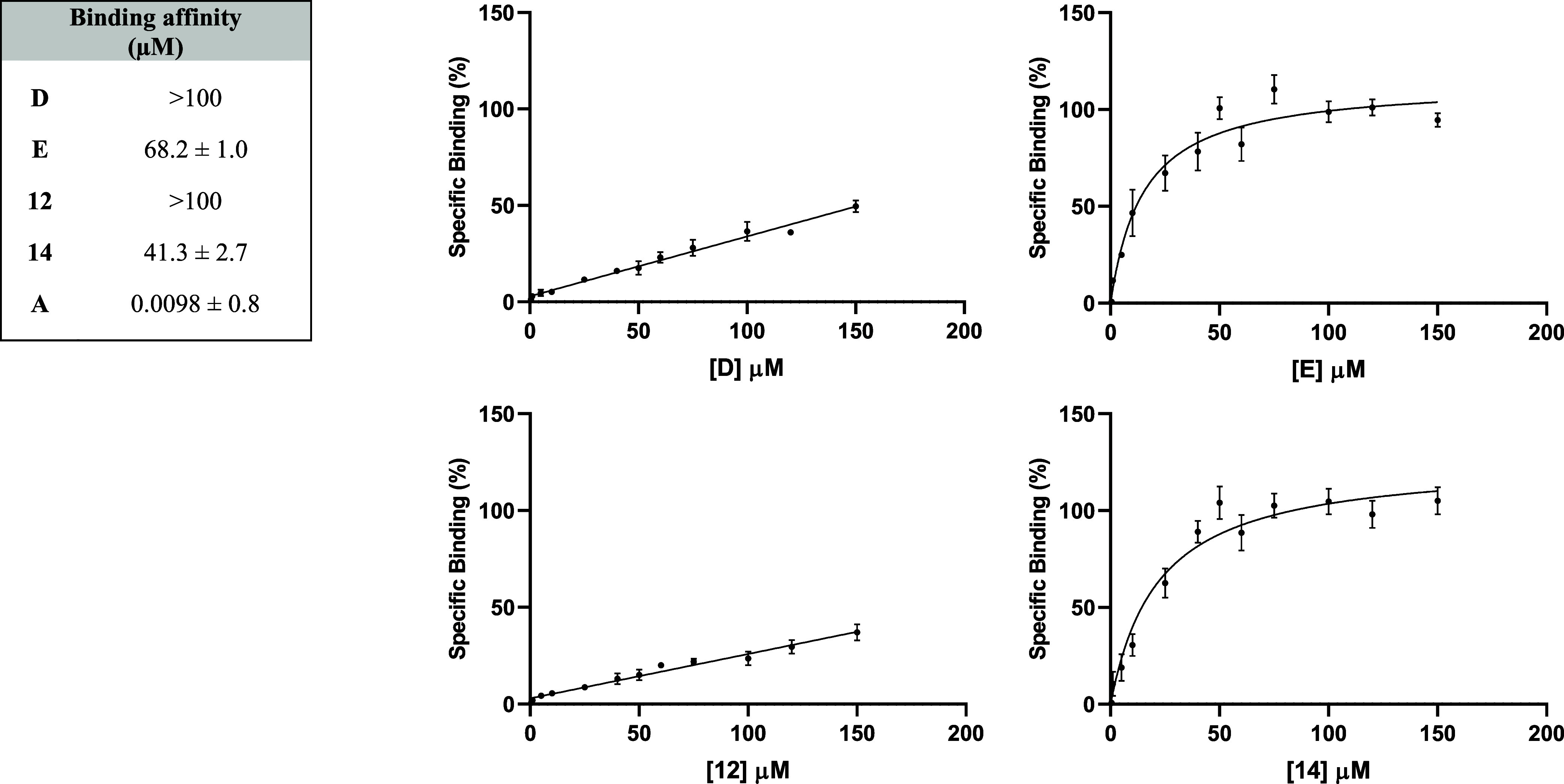
Binding affinity (*K*_D_, μM) of compounds **D**, **E**, **12**, and **14** for α_4_β_1_ integrin determined by saturation binding experiments on intact Jurkat E6.1 cells. Values are reported as *K*_D_ (μM) for the compounds, **D**, **E**, **12**, and **14**, and as *K*_i_ (μM) for compound **A**, as previously reported.^33^ Saturation binding curves to Jurkat E6.1 cells, endogenously expressing α_4_β_1_ integrin. Specific binding was obtained by subtracting the nonspecific binding (in the presence of 50 μM LDV) from the total binding of the conjugated compounds in the presence of vehicle (DMSO). Data shown represent the mean ± SD of three independent experiments, performed in triplicate.

### Assessment of New Theranostic Compounds’ Potency and Selectivity in Cell Adhesion Assays

To define the activity at the receptor and measure integrin-mediated cell adhesion, the theranostic compounds **D**, **E**, **12**, and **14** were tested using cell adhesion assays. First, we assessed their effects on α_4_β_1_ integrin-mediated cell adhesion using Jurkat E6.1 cells. Then, to evaluate compounds’ selectivity, we used erythroleukemic K562 and colorectal adenocarcinoma HT-29 cells, endogenously expressing α_5_β_1_ and α_v_β_6_ integrin, respectively. The three integrins considered are expressed on different types of cancer cells and are involved in tumor progression, invasion, and metastasis,^43–45^ thus representing relevant therapeutic targets for fighting cancer. The results derived from cell adhesion assays are summarized in Table 1, and concentration–response curves are shown in Figures S12–S14 (Supporting Information). Compound **E** was the only compound active toward α_4_β_1_ integrin. It maintained a similar agonist activity to the parental compound **A**, and a good potency in the submicromolar range. Theranostic compound **D** was unable to modulate α_4_β_1_ integrin-mediated cell adhesion, confirming the results obtained with the saturation binding assay, underlying its inability to interact with the receptor. Compound **12** did not modify α_4_β_1_ integrin-mediated cell adhesion, as expected.^33^ Unexpectedly, compound **14**, which showed a good binding to α_4_β_1_ integrin (Figure 4), had no activity on α_4_β_1_ integrin-mediated cell adhesion. As a tentative interpretation of these results, compound **14** may bind to α_4_β_1_ integrin and displace the LDV ligand, yet fail to induce the conformational changes required for receptor activation. This suggests that binding alone is not sufficient to trigger integrin-mediated cell adhesion.

Alternatively, the theranostic moiety of the molecule may interfere with the proper engagement of the β-lactam targeting unit, thereby impairing receptor activation. On the other hand, compound **14** may interact with α_4_β_1_ integrin through a different region of the molecule, leading to a nonproductive binding mode that does not induce the active conformational state of the receptor.^16^

These hypotheses are further complicated by the lack of high-resolution structural information for α_4_β_1_ integrin, as no crystal or cryo-EM structures are currently available, limiting a precise understanding of ligand–receptor interactions.

Following the activity observed with compound **A**, none of the four theranostic compounds was able to interfere with α_5_β_1_ integrin-mediated cell adhesion. In addition, compound **12** acted as a selective agonist for α_V_β_6_, in contrast to the parental compound **A**, previously observed as a highly selective ligand for α_4_β_1_.^33^ Overall, the theranostic compound **E** retained the agonist activity of the parental compound **A** toward α_4_β_1_ integrin. On the contrary, the other theranostics **D**, **12** (both containing 1,8-naphthylimide and 5-FU), and **14** were shown to be mostly inactive toward α_4_β_1_ integrin. Nevertheless, all four compounds were further characterized to evaluate their cytotoxic effects on cancer cells.

### Evaluation of Cell Phototoxicity of the Theranostic Compounds in Various Cell Models

Phototoxicity of the theranostic compounds was assessed via MTT assay across different cell models. The cancer cell lines Jurkat E6.1, K562, and HT-29 cells were chosen to evaluate the potential selective cytotoxic effect of the targeted theranostic agents based on specific integrin expression. Furthermore, HEK-293 cells were used as a noncancer cell model, and as they also express only the β_1_ integrin subunit. First, to verify the low dark toxicity of BODIPY conjugates, Jurkat E6.1, K562, HT-29, and HEK-293 cells were exposed to different concentrations (0.1–50 μM) of **E** or **14** for 24 h without light irradiation. Both compounds displayed no cytotoxicity in the dark (Figure S15, Supporting Information), confirming the low intrinsic cytotoxicity of BODIPY conjugates in the absence of photoactivation. To determine BODIPY-conjugate **E** and **14** photodynamic cytotoxicity, cell lines were treated with increasing concentrations (0.1–50 μM) of the theranostics and incubated for 1 h to enable cellular uptake. Then, cells were irradiated with a low irradiance white light LED for 15 min. After 24 h, an MTT assay was conducted to assess the compounds’ cytotoxic effects. Cell phototoxicity results are shown in Figure 5, and the calculated half-maximal inhibitory concentration (IC_50_) values are summarized in Table 2.

**Figure 5:**
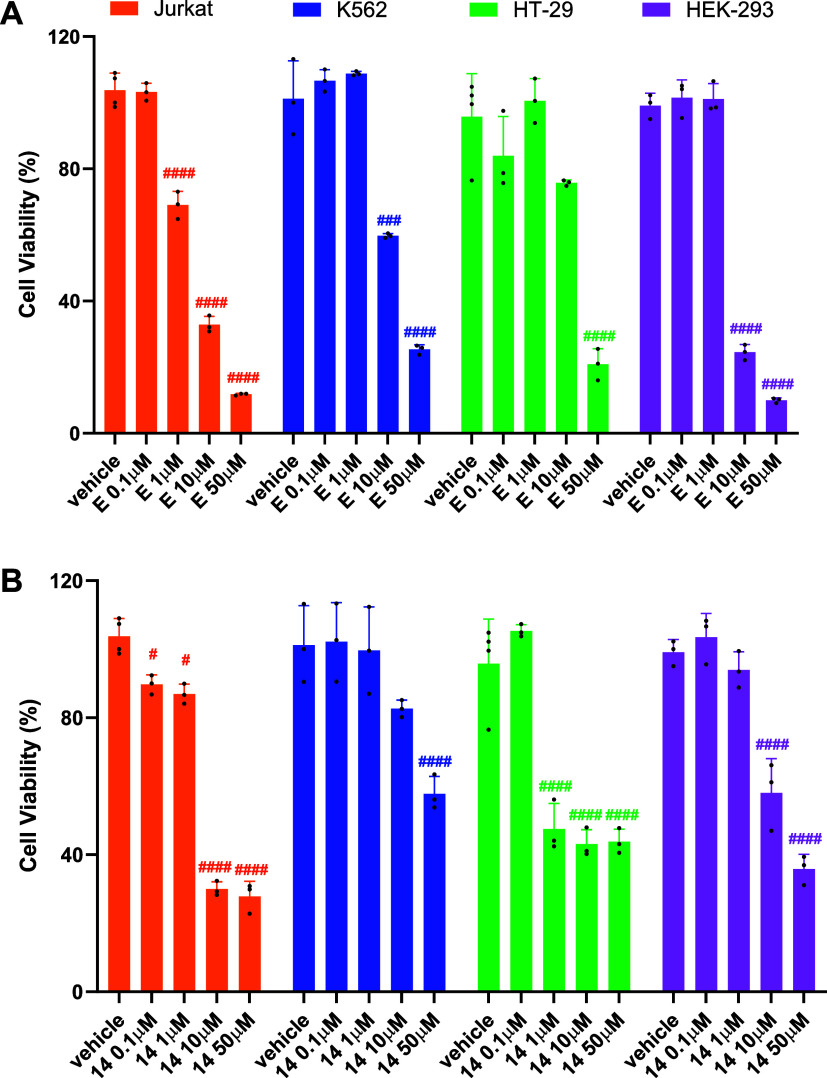
Theranostic compounds **E** and **14** significantly induced a concentration-dependent reduction in cell viability after photosensitization in various cell lines, regardless of the integrin heterodimer expressed. Jurkat E6.1, K562, HT-29, and HEK-293 cells were treated with different concentrations (0.1–50 μM) of **E** (panel A) or **14** (panel B) for 1 h, followed by photosensitization for 15 min; then, the cells were incubated for 24 h, and cell viability was assessed via MTT assay. Data shown represent the mean ± SD of three independent experiments, performed in triplicate. # *p* < 0.05, ### *p* < 0.001, #### *p* < 0.0001 vs vehicle (Dunnett’s test after ANOVA).

**Table 2:** Effects of the Theranostic Compounds **E** and **14** on Cell Viability Measured after Photosensitization via MTT Assay on Different Cell Lines, including Jurkat E6.1 (Expressing α_4_β_1_ Integrin), K562 (Expressing α_5_β_1_), HT-29 (Expressing α_V_β_6_), and HEK-293 (Expressing β_1_) Cells^a^ ^,^ ^b^ ^,^ ^c^

compound	Jurkat/α_4_β_1_	K562/α_5_β_1_	HT-29/α_V_β_6_	HEK-293/β_1_
**E**	2.3 ± 0.2	13.8 ± 1.9	273 ± 15	5.4 ± 0.7
**14**	2.9 ± 0.7	24.7 ± 5.3	0.27 ± 0.06	9.6 ± 1.3

a Values represent the mean ± SD of three independent experiments performed in triplicate.

b The corresponding concentration–response curves used to derive the IC_50_ values are reported in Figure S16 (Supporting Information).

c Data are presented as IC_50_ (μM)

Unexpectedly, although compound **E** behaved as a selective α_4_β_1_ integrin agonist in binding and cell adhesion assays, we observed a significant concentration-dependent reduction in cell viability induced by **E** in all cell models used in this study, regardless of the integrin heterodimers expressed. Moreover, **E** displayed comparable cytotoxic potency in Jurkat E6.1, K562, and HEK-293 cells, and with a less pronounced effect in HT-29 cells. Similarly, compound **14** reduced cell viability in all considered cell lines but exhibited higher potency against HT-29 cells.

The cytotoxicity of 1,8-naphthylimide-5-FU conjugates was also evaluated. Jurkat E6.1, K562, HT-29, and HEK-293 cells were treated with increasing concentrations (0.1–50 μM) of compounds **D** or **12** for 24 h, and cell viability was assessed by MTT assay. Neither compound induced significant changes in cell viability in any of the tested cell models (Figure S17 Supporting Information).

Overall, the cytotoxic effect of BODIPY conjugates **E** and **14** was observed only upon light irradiation, confirming the need for theranostic compound activation and supporting the possible application of BODIPY conjugates for *in vivo* cancer treatment. Disappointingly, although **E** possesses an α_4_β_1_ integrin targeting unit, it showed an unselective behavior, reducing cell viability also in cells that do not express its target. Similarly, **14** displayed an undiscriminating cytotoxic effect, despite its ability to bind α_4_β_1_ integrin.

This unexpected behavior of both **E** and **14** may stem from an increased lipophilicity of the whole molecule caused by conjugation with BODIPY, resulting in a higher nonspecific cellular uptake.^30^ In the literature, some examples of BODIPY probe conjugated with RGD (cRGD) peptides for α_v_β_3_ integrin targeting were reported.^29,30^ In those cases, the lipophilicity of the BODIPY moiety was counterbalanced by a large number of hydrogen bond donor/acceptor (HD/HA) groups due to the peptide fragment and with some residues of poly­(ethylene glycol) chains (PEG). Those molecules largely exceeded the Lipinski’s rule of five for druggable molecules, also due to their high molecular weights (MW). However, for a smaller compound such as **E**, characterized by a restricted number of HD/HA groups and lower MW, the lipophilic interactions with the cellular membrane likely prevailed over the specific interaction with integrins. This highlights the need for a careful balance between lipophilicity and polar functionalities in the design of integrin-targeting theranostic agents to preserve selectivity.

### Cellular Uptake of Compounds **E** and **14**

To verify our hypothesis of nonspecific cellular uptake, we evaluated the extent of cellular internalization of compounds **E** and **14** by measuring cell fluorescence through flow cytometry. The same cell lines used in the previous experiments (Jurkat E6.1, K562, HT-29, and HEK-293 cells) were exposed to varying concentrations (0.1–50 μM) of the theranostics **E** and **14** for 1 or 2 h. Cellular uptake was also measured for compounds **D** and **12**, but neither of them was internalized (data not shown). On the contrary, internalization of both **E** and **14** was observed in all cell lines used, regardless of the integrin heterodimer expressed (Figure 6), at both time points considered. Cellular uptake was concentration-dependent and greater for compound **E** in K562 and HT-29 cells, which do not express α_4_ integrin. Compound **14** was internalized to a greater extent than **E** in all cell lines, in a concentration- and time-dependent manner (Figure 6B). Overall, these results confirmed the nonspecific cell internalization of both **E** and **14**, which could be due to a higher lipophilicity of the entire theranostic molecules (*c* log *P* = 6.17 and 8.36, respectively, Table 1)^30^ compared to the model compound **A** (*c* log *P* = 1.26). In this hypothesis, changing the imaging portion with a more hydrophilic moiety with a lower *c* log *P* value could improve the specificity of internalization. The results from internalization experiments are consistent with the unselective behavior of **E** and **14** observed in the cytotoxicity assay. Likely, the integrin targeting action of compound **A**, present in the theranostic agent **E** conjugated with BODIPY, was overcome by the substantial lipid-like nature of the whole molecule, abolishing completely an eventual integrin-mediated and selective cytotoxic effect.

**Figure 6:**
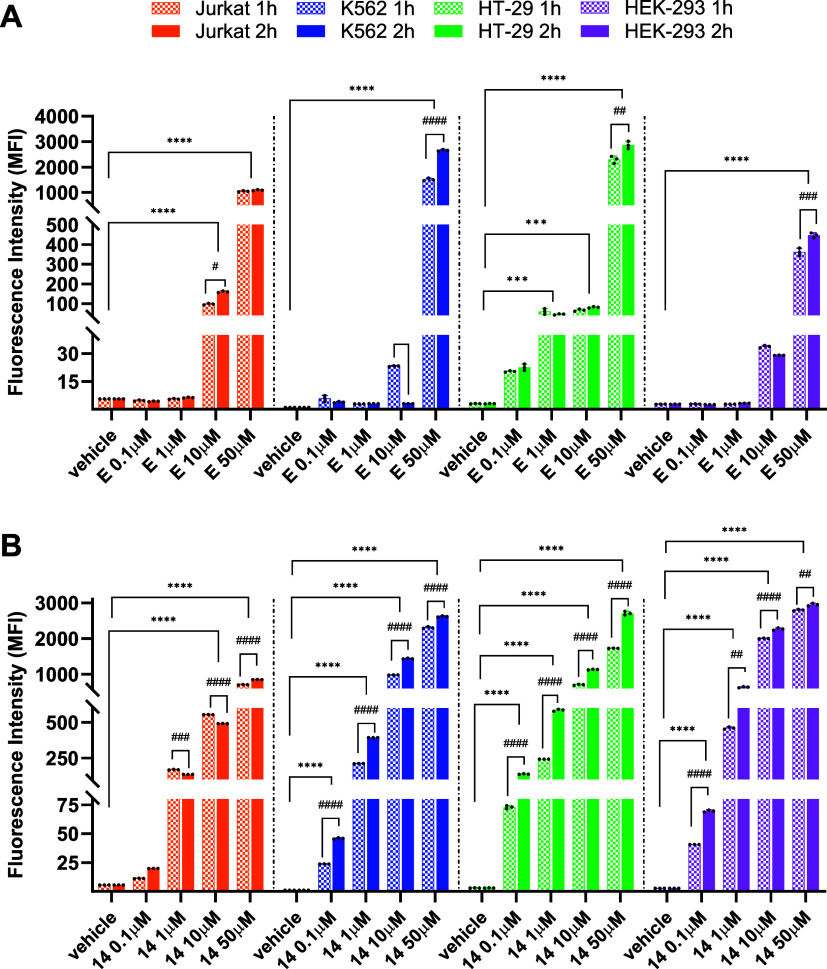
Cellular uptake of theranostic compounds **E** (panel A) and **14** (panel B) by Jurkat E6.1, K562, HT-29, and HEK293 cells. Cells were incubated with **E** or **14** (0.1 – 50 μM) or the vehicle alone (vehicle) for 1 or 2 h. The fluorescence intensity of the cells (MFI: mean fluorescence intensity, arbitrary units) corresponds to theranostics intracellular uptake and was quantified by flow cytometry. Values are mean ± SD from three independent experiments conducted in triplicate. ***p < 0.001; ****p < 0.0001 vs the vehicle of the same cell line; #*p* < 0.05, ## *p* < 0.01, ### *p* < 0.001, ####*p* < 0.0001 vs 1 h treatment (Dunnett’s test after ANOVA).

## Conclusion

In conclusion, we designed and synthesized two novel compounds **D** and **E** to act as theranostic agents. The design of new molecules was inspired to β-lactam **A**, taken as a model of a selective and potent agonist of integrin α_4_β_1,_ and to compounds **B** and **C** as conjugates of the parent compound **A** with 5-FU as cytotoxic agent, and FITC as fluorophoric residue, respectively, to study cell internalization. Compound **D** has a β-lactam portion to target α_4_β_1_ integrin, 1,8-naphthylimide for imaging, and the cytotoxic 5-FU, whereas compound **E** has the β-lactam portion and BODIPY as photosensitizer and cytotoxic agent. The synthesis of the two molecules was accomplished by a careful selection of the synthetic strategy, reagents, and reaction conditions to ensure compatibility with the functional groups of the target molecules. Both compounds exhibited good quantum yield for fluorescence, absorbance, and emission properties. Only compounds **E** and **14** were able to bind to α_4_β_1_ integrin with a good affinity in the micromolar range. In adhesion tests with Jurkat E6.1, K562, HT-29, and HEK-293 cells, compound **E** retained the selective agonist activity of the parent β-lactam ligand **A** toward α_4_β_1_ integrin, whereas **12** emerged as a selective agonist of cell adhesion mediated by α_v_β_6_ integrin. After photosensitization, compound **E** showed a nonspecific cytotoxic effect significantly inducing a concentration-dependent reduction in cell viability of Jurkat E6.1, K562, HT-29, and HEK-293 cells, regardless of the expressed integrins. Results from internalization experiments are consistent with an unselective behavior of **E** observed in the cytotoxicity assay. This behavior was in contrast with the selective internalization of previously studied parent compound **C**, and could be due to the prevalence of high lipophilicity of **E** with respect to the action of structural elements for a specific integrin recognition. Further studies are in due course to increase the hydrophilicity of the new molecules and to address a more efficient recognition by specific integrins.

## Materials and Methods

Commercial reagents were used as received without additional purification. ^1^H and ^13^C NMR spectra were recorded with an INOVA 400 and a Bruker Avance 600 MHz instrument with a 5 mm probe. All chemical shifts were quoted relative to deuterated solvent signals (δ in ppm and J in Hz). ATR-FTIR spectra of pure compounds were recorded with a Bruker α instrument and with an Agilent Technologies CARY 630 FTIR, in transmittance mode with a 4 cm^–1^ resolution in the 4000–400 cm^–1^ range. The purities of the target compounds **D** and **E** were assessed as being >95% using UPLC. UPLC-MS analyses were performed with an Agilent Technologies 1260 Infinity II instrument, coupled with an Agilent Technologies Infinity Lab LC/MSD XT single-quadrupole mass spectrometer in full scan mode from *m*/*z* = 50 to 2600, in positive ion mode. The HPLC is equipped with a Phenomenex Gemini 3 μm C18 (100 mm × 3 mm) column; the following method was used: H_2_O/ACN, 0.4 mL/min; gradient from 30 to 80% of ACN in 8 min, 80% of ACN until 25 min, 40 °C. Compounds **3**,^31^ **4**,^47^ and **13** ^41^ were synthesized accordingly to already published procedures. Experimental procedures for compounds **5**-**14**, **D** and, **E** are reported in Supporting Information. All tested compounds have a purity ≥90%

### Cell Culture

Jurkat E6.1 (immortalized cell line from human blood leukemic T-cell lymphoblasts, expressing α_4_β_1_ integrin), HT-29 (human colorectal adenocarcinoma cell line, expressing α_v_β_6_ integrin) and K562 (human erythroleukemic cell line, expressing α_5_β_1_ integrin) were routinely grown in RPMI-1640 (Life Technologies, Carlsbad, CA, USA) supplemented with 1% l-glutamine and 10% FBS (fetal bovine serum, Life Technologies). K562 cells were treated with 65 nM PMA (phorbol 12-myristate 13-acetate, Sigma-Aldrich, Milan, Italy) 48 h before the experiments to induce differentiation and, consequently, to increase α_5_β_1_ integrin expression. HEK-293 (human embryonic kidney cell line, expressing β_1_ integrin subunit), were cultured in MEM (minimum essential medium, Life Technologies) supplemented with 1% l-glutamine, 1% NEAA (nonessential amino acids, Life Technologies), and 10% FBS. Cells were kept at 37 °C under 5% CO_2_ humidified atmosphere. All cell lines were purchased from American Type Culture Collection (ATCC, Rockville, MD, USA) and routinely tested for mycoplasma contamination. The cell models employed in this study are widely used to investigate potential agonists or antagonists of cell adhesion as well as integrin-mediated selective cellular uptake.^31^

### Saturation Binding Assay

To determine theranostic compounds’ binding affinity for α_4_β_1_ integrin, a saturation binding assay was performed using Jurkat E6.1 cells and increasing concentrations of theranostic compounds in the presence of either vehicle (DMSO) or 50 μM LDV (Tocris Bioscience). Jurkat E6.1 cells (50,000 cells/sample) were collected and suspended in 0.1% BSA in HEPES Buffer (NaCl 110 mM; KCl 10 mM; Glucose 10 mM; MgCl_2_ 1 mM; CaCl_2_ 1.5 mM; HEPES 30 mM; pH 7.4; 0.1% BSA w/v). Cells were then preincubated with increasing concentrations of the theranostic compounds (10^–7^ – 10^–4^ M) or vehicle (DMSO) for 30 min at room temperature in the dark. LDV (50 μM, Tocris Bioscience) was added to Jurkat E6.1 cells and incubated for another 30 min at room temperature in the dark. At the end of the incubation with LDV, Jurkat E6.1 cells were washed with 0.1% BSA in HEPES Buffer and plated in a black 96-well plate (Corning Costar) to determine fluorescence intensity. 1,8-naphthylimide (Ex364 nm/Em473 nm, comp. **D** and **12**) or BODIPY (Ex499 nm/Em511 nm, comp. **E** and **14**) fluorescence intensity was measured using an EnSpire Multimode Plate Reader (PerkinElmer, Waltham, MA, USA). Nonspecific binding (NSB) was determined in the presence of unlabeled LDV; specific binding was measured by subtracting the NSB from the total cell-bound fluorescence in the presence of vehicle (DMSO).

Using nonlinear regression analysis with GraphPad Prism 10.4.1, the equilibrium dissociation constant (*K*_D_) values were determined. Each experiment was carried out in triplicate and repeated at least 3 times.

### Cell Adhesion Assays

The cell adhesion assays were performed as previously reported.^31,32^ Briefly, regarding adhesion assay on Jurkat E6.1, K562, and HT-29 cells, clear 96-well plates (Corning Costar) were coated overnight at 4 °C by passive adsorption with FN (fibronectin, 10 μg/mL; Sigma-Aldrich, Milan, Italy). For adhesion assays mediated by α_5_β_1_ integrin, K562 cells were treated with 64.85 nM PMA for 48 h to enhance α_5_β_1_ integrin expression. The day after, FN-coated 96-well plates were blocked with 1% BSA in HBSS (Hank’s Balanced Salt Solution, Life Technologies) for 30 min at 37 °C for adhesion assays on Jurkat E6.1 and HT-29 cells; plates to be used for K562 cells were blocked with 1% BSA in PBS (Phosphate-buffered saline, Life Technologies) for 1 h at 37 °C. Cells were counted and preincubated with increasing concentrations of the compounds (10^–10^ – 10^–4^ M) or the vehicle (DMSO) for 30 min at 37 °C (Jurkat E6.1 and HT-29 cells) or 30 min at room temperature (K562 cells). Then, cells were plated (50,000 cells/well) and incubated in FN-coated wells (30 min at 37 °C for Jurkat E6.1; 90 min at room temperature for HT-29; 1 h at room temperature for K562). All the wells were then washed three times with blocking solution to remove nonadherent cells, and 50 μL/well of hexosaminidase substrate [1:1 solution of 4-nitrophenyl-*N*-acetyl-β-d-glucosaminide 7.5 mM in 0.09 M citrate buffer (pH 5.0) and 0.5% Triton X-100 in H_2_O] was added and incubated for 1 h at room temperature. Hexosaminidase substrate is transformed into 4-nitrophenol by β-*N*-acetylglucosaminidase, and absorbance was measured at 405 nm after the addition of stopping solution (Glycine 50 mM; EDTA 5 mM; pH 10.4) using an EnSpire Multimode Plate Reader (PerkinElmer, Waltham, MA, USA).

The number of adherent cells was determined via comparison with a standard curve made in the same plate. Experiments were carried out in quadruplicate and repeated at least three times. Data analysis and IC_50_ or EC_50_ values were calculated using GraphPad Prism 10.4.1 (GraphPad Software, San Diego, CA, USA).

### Cell Viability-MTT Assay

Cell viability after 24 h exposure to BODIPY-conjugated compounds **E** and **14** was assayed by means of the MTT assay as previously reported,^48^ with the following modifications. Cell cultures (Jurkat E6.1 or K562 cells) or cell suspensions after trypsinization (HT-29 or HEK-293 cells) were stained with 0.4% erythrosine, and cell density was then determined by microscopic counting. 15,000 cells/sample were then seeded into each well of a 96-well plate and incubated at 37 °C for a suitable time (1 h for suspension cells and 24 h for adherent cells). Cells were then treated with compounds **E** or **14** (0.1 – 1 – 10 – 50 μM) for 1 h at 37 °C, to enable compounds’ cellular uptake. Afterward, to allow singlet oxygen generation in BODIPY-conjugated ligands **E** and **14**, cells were irradiated for 15 min with a low irradiance white light LED, which has a peak emission at λ_max_ = 668 ± 3 nm; 24 mW/cm^2^ at room temperature. These conditions were determined by comparing the efficacy of several irradiation time points (from 15 to 30 min). Experiments were repeated in the absence of BODIPY-conjugated compounds photosensitization, to confirm its necessity to induce a cytotoxic effect. Following photosensitization, cells were incubated for 24 h at 37 °C. MTT (0.25 mg/mL; Sigma-Aldrich, Milan, Italy) was then added, and the plate was incubated for 4 h at 37 °C. After that, dimethyl sulfoxide (DMSO; Sigma-Aldrich, Milan, Italy) was directly added into each well to dissolve formazan. The plate was shaken for 5 min at room temperature, and absorbance was measured at 570 nm using an EnSpire Multimode Plate Reader (PerkinElmer, Waltham, MA, USA). Experiments were carried out in triplicate and repeated at least three times. Data were represented by a graph showing the percentage of viable cells versus vehicle. No phototoxicity was detected from the irradiation only without compound treatment.

### Cellular Uptake

Intracellular uptake of BODIPY-conjugated compounds was evaluated by flow cytometry as previously reported,^31^ with the following adjustments. Jurkat E6.1 and K562 cells (50,000 cells/sample) were suspended in blocking solution (1% BSA in HBSS) and treated with compounds **E** or **14** (0.1 – 1 – 10 – 50 μM) for 1 or 2 h at 37 °C. HT-29 and HEK-293 cells were seeded in a 24-well plate (50,000 cells/well) 48 h before the experiments and then treated as previously described. Afterward, samples were washed three times with blocking solution, and cellular uptake was quantified by flow cytometry on a Guava easyCyte 5 flow cytometer (Merck Millipore, Vimodrone, Italy).

### Statistical Analysis

All assays were carried out in triplicate for each sample. Continuous variables are presented as mean ± standard deviation when normally distributed; data were tested using one-way ANOVA followed by Dunnett post-test. Data analysis and IC_50_ values referring to binding and adhesion assays were fitted using a sigmoidal dose–response equation using GraphPad Prism software. Statistical analyses were performed using GraphPad Prism (version 10.4.2; GraphPad Software, Inc., La Jolla, CA, USA). *P* < 0.05 was considered significant.

## Supplementary Material



## Notes

Additional experimental details, materials, and methods. ^1^H, ^13^C and ^19^F NMR spectra for all compounds (PDF)
