# Supplementary material for: Questing for Integrin Targeting Theranostics for Cancer Cell-Selective Molecules
Source: ACS Omega. 2026 May 5;11(19):28471–83. doi: 10.1021/acsomega.6c00193 (PMC13191554; doi:10.1021/acsomega.6c00193)
Supplement: Supplementary file 1 [file ao6c00193_si_001.pdf]

Questing for Integrin Targeting Theranostics for Cancer Cell-selective Molecules.

Valentina Giraldi, Tania Pecoraro, Andrea Maurizio, Alessia Milana, Luisa De Cola, Monica Baiula,* and Daria Giacomini*

Supporting Information

Table of contents

1. Reaction conditions and synthesis of compound 10. Table S1 and Scheme S1 page S2
2. Experimental procedures for compounds 5-14, D, and E pages S3-S8
3. Copies of ^{1}H, ^{13}C and ^{19}F NMR spectra pages S9-S27
Figure S1. ^{1}H, ^{13}C and ^{19}F NMR spectra of compound 5 pages S9-S10
Figure S2. ^{1}H, ^{13}C and ^{19}F NMR spectra of compound 6 pages S11-S12
Figure S3. ^{1}H, and ^{13}C spectra of compound 7 page S13
Figure S4. ^{1}H, and ^{13}C spectra of compound 8 page S14
Figure S5. ^{1}H, and ^{13}C spectra of compound 9 page S15
Figure S6. ^{1}H, ^{13}C and ^{19}F NMR spectra of compound 10 pages S16-S17
Figure S7. ^{1}H, ^{13}C and ^{19}F NMR spectra of compound 11 pages S18-S19
Figure S8. ^{1}H, ^{13}C and ^{19}F NMR spectra of compound 12. pages S20-S21
Figure S9. ^{1}H, ^{13}C and ^{19}F NMR spectra of compound D. pages S22-S23
Figure S10. ^{1}H, ^{13}C and ^{19}F NMR spectra of compound 14. pages S24-S25
Figure S11. ^{1}H, ^{13}C and ^{19}F NMR spectra of compound E. pages S26-S27
4. Concentration-response curves from cell adhesion assays for compounds **D**, **E**, **12**, and **14** pages S28-S29
Figure S12. Concentration-response curves from cell adhesion assays on $\alpha_4\beta_1$ integrin-mediated Jurkat E6.1 cell adhesion to FN. page S28
Figure S13. Concentration-response curves from cell adhesion assays on $\alpha_5\beta_1$ integrin-mediated K562 cell adhesion to FN. page S29
Figure S14. Concentration-response curves from cell adhesion assays on $\alpha_V\beta_6$ integrin-mediated HT-29 cell adhesion to FN. page S29
5 Cell viability assay in various cell lines treated with the theranostic compounds (Figures S15-S17) page S30-S32
Figure S15. Effects of compounds **E** and **14** on cell viability without photosensitization page S30
Figure S16. Concentration-response curves from MTT assays for compounds **E** and **14** on Jurkat E6.1, K562, HT-29, and HEK-293 cells to determine IC_{50} values page S31
Figure S17. Effects of compounds **D** and **12** on cell viability page S32
6 HPLC-MS spectra of target compounds **D** and **E** pages S33-S34
Figure S18. HPLC-MS spectra of compound **D** page S35
Figure S19. HPLC-MS spectra of compound **E** page S36

1. Table S1. Reaction conditions for the synthesis of compound **10**.

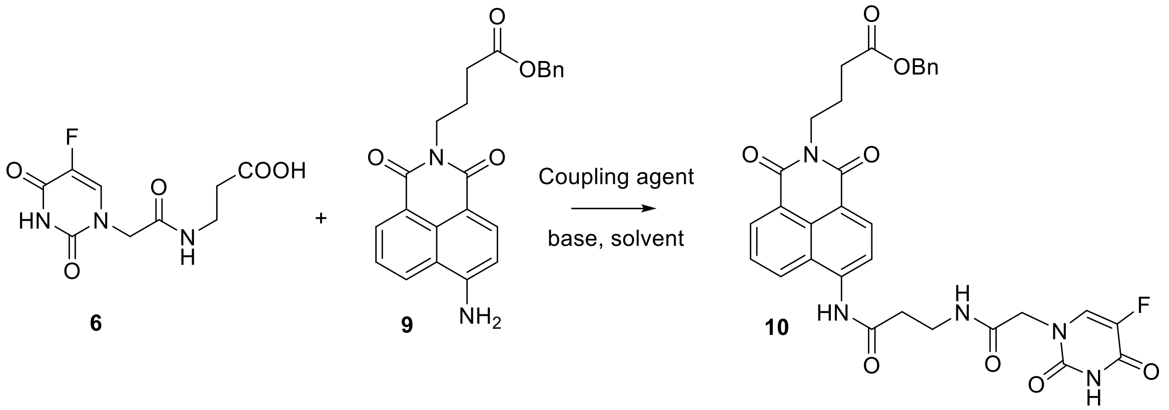

Entry	Amine 9 (eq.)	Acid 6 (eq.)	Solvent	Coupling agent (eq.)	Base (eq.)	T	Time	Yield of 10[a] (%)
**1**	1.2	1	DMF:DCM 1:5	EDC (1) HOBt (1)	TEA (2)	0°C to rt	24 h	-
**2**	1	1.2	ACN	EDC (1) HOBt (0.1)	DIPEA (5) DMAP (1)	rt	72 h	-
**3**	1	1	DMF	DCC (1.1) NHS (1.1)	TEA (1.5)	0°C to 45°C to 80°C	72 h	19
**4**	1	1	DMF	EDC (1.1) NHS (1.1)	TEA (2)	0°C to 45°C to 80°C	5 d	22
**5**	1	1.2	DMF	T3P (2)	DIPEA (4)	0°C to 60°C to 80°C	24 h	20

[a] Isolated yields after flash chromatography.

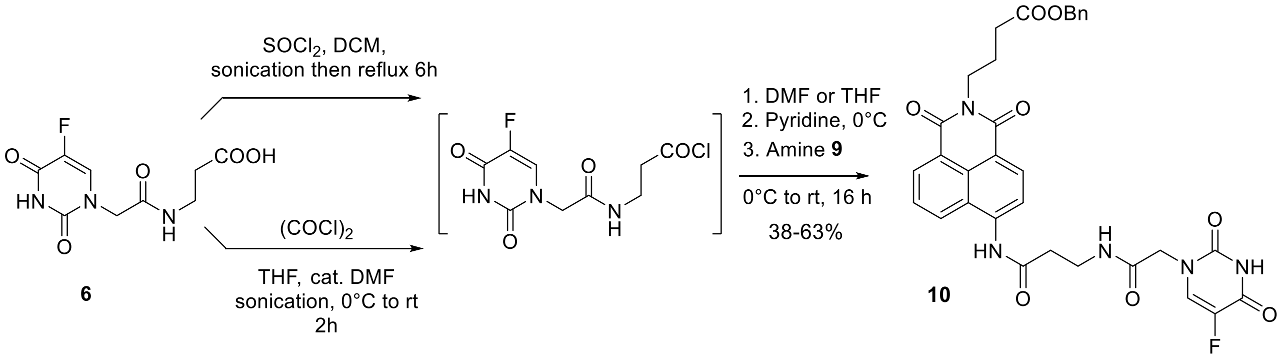

Scheme S1. Synthesis of intermediate **10** via an acyl chloride intermediate formation.

2. Experimental procedures for compounds 5-14, D and E.

General procedure for benzyl ester deprotection by hydrogenolysis (GP1) In a two-neck round bottom flask under nitrogen atmosphere, the starting material was dissolved in the solvent indicated in the specific preparation above. Pd/C was added and H_2 supplemented *via* a lattice balloon. The reaction was stirred under hydrogen atmosphere at room temperature and monitored by TLC. At completion, the reaction mixture was filtered under vacuum onto a celite pad, washing with the solvent. The filtrate was dried under reduced pressure, yielding the target compound without further purifications unless indicated.

Benzyl 3-(2-(5-fluoro-2,4-dioxo-3,4-dihydropyrimidin-1(2H)-yl)acetamido)propanoate (5) In a round bottom flask under nitrogen atmosphere, β-alanine benzylester PTSA salt (274 mg, 0.78 mmol, 1.2 equiv.) and TEA (126 µL, 0.91 mmol, 1.4 equiv.) in anhydrous DCM (3.3 mL) were stirred for 15 min. Meanwhile, in another round bottom flask equipped with a dropping funnel, under nitrogen atmosphere, the acid **4** (123 mg, 0.65 mmol, 1 equiv.) was dissolved in anhydrous DMF (0.85 mL). At 0°C, HOBt (88 mg, 0.65 mmol, 1 equiv.) and EDC (124 mg, 0.65 mmol, 1 equiv.) were added. After 10 minutes, the solution of the desalted β-alanine benzylester was transferred from the first flask into the dropping funnel and slowly dropped at 0°C into the second flask. After 1 h at 0°C, the mixture was stirred for 2 days at r.t. At completion (TLC monitoring), the reaction mixture was dried under reduced pressure to remove DCM and most of the DMF. Then the crude was diluted with EtOAc (15 mL) and water (15 mL) was added. The organic phase was separated and washed with 0.1 M HCl (10 mL x 2) and with saturated aqueous solution of $NaHCO_3$ (3x10 mL). The collected organic layers were dried on Na_2SO_4, filtered and the solvent removed under reduced pressure to afford compound **5** in 78% yield (177 mg, 0.51 mmol) without further purification. ^1H NMR (400 MHz, DMSO-d_6) δ 8.31 (t, *J* = 5.6 Hz, 1H), 7.99 (d, *J* = 6.8 Hz, 1H), 7.44 – 7.27 (m, 5H), 5.10 (s, 2H), 4.25 (s, 2H), 3.34 (q, *J* = 6.5 Hz, 3H), 2.55 (t, *J* = 6.8 Hz, 2H). ^{19}F NMR (376 MHz, DMSO-d_6) δ -170.68 (d, *J* = 6.8 Hz). ^{13}C NMR (100 MHz, DMSO-d_6) δ 171.1, 166.6, 157.6 (d, *J* = 25.9 Hz), 149.7, 139.3 (d, *J* = 228.2 Hz), 136.1, 131.0 (d, *J* = 33.7 Hz), 128.5, 128.0, 127.9, 65.6, 49.5, 34.8, 33.6. ATR-FTIR (cm^{-1}): 3353, 3008, 2958, 2829, 1724, 1694, 1660, 1542, 1305, 1217, 1166, 910, 750, 697. HPLC-MS (ESI+): t_R= 3.91 min; m/z = 372 $[M+Na]^+$, 388 $[M+K]^+$.

3-(2-(5-Fluoro-2,4-dioxo-3,4-dihydropyrimidin-1(2H)-yl)acetamido)propanoic acid (6) Following GP1, compound **6** was obtained from **5** (185 mg, 0.53 mmol) in a 1:1 mixture of anhydrous methanol and THF (5.8 mL total volume) with 10% wt of Pd/C (18.5 mg) and H_2, in 98% yield (135 mg, 0.52 mmol). ^1H NMR (400 MHz, DMSO-d_6) δ 8.2 (t, *J* = 5.6 Hz, 1H), 8.0 (d, *J* = 6.8 Hz, 1H), 4.2 (s, 2H), 3.3 (q, *J* = 6.7 Hz, 2H), 2.4 (t, *J* = 6.8 Hz, 2H).^{19}F NMR (376 MHz, DMSO-d_6) δ -170.75 (d, *J* = 6.8 Hz). ^{13}C NMR (100 MHz, DMSO-d6) δ 172.8, 166.5, 157.6 (d, J = 25.7 Hz), 149.7, 139.2 (d, J = 227.9 Hz), 131.1 (d, J = 33.8 Hz), 49.5, 35.0, 33.9· ATR-FTIR (cm^{-1}): 3330, 3034, 2995, 2810, 1771, 1697, 1671, 1651, 1542, 1487, 1443, 1284, 547. HPLC-MS (ESI+): t_R= 1.26 min; m/z = 260 $[M+H]^+$, 282 $[M+Na]^+$.

Benzyl 4-(6-bromo-1,3-dioxo-1H-benzo[de]isoquinolin-2(3H)-yl)butanoate (7) In a two neck round bottom flask under inert atmosphere (N_2), 4-Bromo-1,8-naphthalic anhydride (250 mg, 0.902 mmol, 1 equiv.) was dissolved in anhydrous DMF (4.5 mL) and then γ-aminobutyric acid benzyl ester PTSA salt (494 mg, 1.35 mmol, 1.5 equiv.) was added followed by TEA (250 µL, 1.81 mmol, 2 equiv.). The reaction was stirred and warmed at 100 °C. At completion (TLC monitoring, 3 h), the reaction was diluted with 50 mL of diethylether and washed with water (2x50 mL) and brine. The organic layer was dried on Na_2SO_4

and the solvent removed under reduced pressure. The desired compound **7** was obtained without further purification as a brown solid (343 mg, 0.758 mmol) in 84% yield.

^{1}H NMR (400 MHz, $CDCl_3$): δ 8.59 (dq, *J* = 7.3, 1.2 Hz, 1H), 8.49 (dq, *J* = 8.4, 1.3 Hz, 1H), 8.34 (dd, *J* = 7.9, 2.1 Hz, 1H), 7.97 (dd, *J* = 7.9, 2.0 Hz, 1H), 7.79 (ddt, *J* = 7.9, 6.7, 1.5 Hz, 1H), 7.39 – 7.22 (m, 5H), 5.08 (s, 2H), 4.23 (d, *J* = 7.7 Hz, 2H), 2.50 (t, *J* = 7.5 Hz, 2H), 2.11 (p, *J* = 7.3 Hz, 2H). $^{13}C\{^1H\}$ NMR (100 MHz, $CDCl_3$): δ 172.7, 163.7, 163.7, 136.4, 133.4, 132.2, 131.3, 131.2, 130.7, 130.4, 129.0, 128.6, 128.3, 128.3, 128.2, 123.0, 122.2, 66.4, 39.8, 32.0, 23.5. ATR-FTIR (cm^{-1}): 3031, 2959, 2000, 1800, 1657, 1568, 1525, 1187, 618. Melting point: 85°C. HPLC-MS (ESI^+): t_R= 9.32 min; m/z = 452 $[M+H]^+$.

Benzyl 4-(6-azido-1,3-dioxo-1H-benzo[de]isoquinolin-2(3H)-yl)butanoate (8) In a round bottom flask under inert atmosphere (N_2), compound **7** (252 mg, 0.56 mmol, 1 equiv.) was added and dissolved in DMF (2.6 mL). Sodium azide (44 mg, 0.67 mmol, 1.2 equiv.) was dissolved in 260 µL of water and slowly added. The reaction was kept at 60°C for 4 hours, 1 hour at 80°C and left overnight at RT. At completion (TLC monitoring) the reaction was quenched with water (10 mL) and extracted with EtOAc (3x15 mL). The organic layers were dried on Na_2SO_4 and the solvent removed under reduced pressure. The crude was dried under high vacuum and the compound **8** was obtained as a brown solid without further purification in 96% yield (222 mg, 0.536 mmol).

^{1}H NMR (400 MHz, $CDCl_3$): δ 8.52 (dd, *J* = 7.3, 1.2 Hz, 1H), 8.44 (d, *J* = 8.0 Hz, 1H), 8.32 (dd, *J* = 8.4, 1.1 Hz, 1H), 7.65 (dd, *J* = 8.4, 7.3 Hz, 1H), 7.36 – 7.23 (m, 6H), 5.07 (s, 2H), 4.20 (t, *J* = 7.1 Hz, 2H), 2.49 (t, *J* = 7.6 Hz, 2H), 2.10 (p, *J* = 7.4 Hz, 2H). $^{13}C\{^1H\}$ NMR (100 MHz, $CDCl_3$): δ 172.8, 164.0, 163.6, 143.5, 136.1, 132.3, 131.8, 129.2, 128.9, 128.6, 128.3, 128.2, 126.9, 124.4, 122.6, 118.9, 114.8, 66.4, 39.6, 32.0, 23.5. ATR-FTIR (cm^{-1}): 2996, 2898, 2852, 2121, 2000-1800, 1651, 1525, 1454, 1248.

Benzyl 4-(6-amino-1,3-dioxo-1H-benzo[de]isoquinolin-2(3H)-yl)butanoate (9) In a round bottom flask, compound **8** (277 mg, 0.67 mmol, 1 equiv.) was dissolved in DMF (5.5 mL). At 0°C, NaHS monohydrate (199 mg, 2.68 mmol, 4 equiv.) dissolved in water (0.55 mL) was slowly dropped. After 10 minutes at 0°C, the mixture was warmed to 60°C and the reaction was monitored by TLC. Another equivalent of NaHS (50 mg dissolved in 0.1 mL of water) was added after 7 hours at RT and left stirring overnight at RT. At completion, the reaction was quenched with water (10 mL) and extracted with EtOAc (3x15 mL). The organic layer was dried on Na_2SO_4, filtered and the solvent removed under reduced pressure and high vacuum. The crude was purified by flash chromatography on silica gel (98:2 DCM:MeOH) to afford the desired product **9** in 75% yield (195 mg, 0.50 mmol) as an orange solid. ^{1}H NMR (400 MHz, $CDCl_3$): δ 8.56 (dd, *J* = 7.3, 1.1 Hz, 1H), 8.38 (d, *J* = 8.2 Hz, 1H), 8.14 – 8.03 (m, 1H), 7.62 (dd, *J* = 8.4, 7.3 Hz, 1H), 7.44 – 7.22 (m, 5H), 6.85 (d, *J* = 8.2 Hz, 1H), 5.07 (d, *J* = 11.9 Hz, 4H), 4.24 (t, *J* = 7.0 Hz, 2H), 2.50 (t, *J* = 7.7 Hz, 2H), 2.12 (h, *J* = 6.8 Hz, 2H).$^{13}C\{^1H\}$ NMR (100 MHz, $CDCl_3$): δ 173.2, 164.7, 164.1, 149.9, 136.0, 134.0, 131.5, 129.9, 128.6, 128.2, 128.2, 127.4, 124.8, 122.8, 120.0, 111.4, 109.5, 66.4, 39.3, 32.1, 23.6. ATR-FTIR (cm^{-1}): 3356, 3257, 2995, 2926, 2852, 2000-1800, 1678, 1525, 1457, 1243. Melting Point: 51°C. HPLC-MS (ESI^+): t_R= 4.686 min; m/z =389 $[M+H]^+$.

Benzyl 4-(6-(3-(2-(5-fluoro-2,4-dioxo-3,4-dihydropyrimidin-1(2H)-yl)acetamido)propanamido)-1,3-dioxo-1H-benzo[de]isoquinolin-2(3H)-yl)butanoate (10)

Procedure with oxalyl chloride in THF. The acid **6** (56 mg, 0.216 mmol, 1 equiv.) was added in a Schlenk tube under nitrogen atmosphere. Anhydrous THF was added (2 mL) and the mixture was stirred and sonicated for 5 minutes. The resulting suspension was cooled to 0°C and oxalyl chloride was slowly

dropped (46 µL, 0.541 mmol, 2.5 equiv.). The mixture was kept at 0°C for 1 hour and at r.t. for further 2 hours. Then, THF and residual oxalyl chloride were removed under vacuum. In the same Schlenk tube, the thus obtained white solid was then dissolved at 0°C in anhydrous DMF (0.5 mL) and pyridine (17 µL, 0.216 mmol, 1 equiv.), and finally the amine **9** (40 mg, 0.103 mmol, 0.5 equiv.) dissolved in THF (1.5 mL) were dropped. After 10 minutes at 0°C the mixture was stirred overnight at r.t. and monitored by TLC. THF was then removed under reduced pressure, the mixture was diluted with EtOAc and water (5 mL). After 3 extractions with ethyl acetate (3x 5 mL), the collected organic layers were dried on Na_2SO_4, filtered and the solvent removed under reduced pressure. The crude was purified by dissolving it into 5 mL of hot methanol and filtering on a paper filter, washing with hot methanol. The yellow solid was recovered and the product **10** was obtained in 52% yield (34.2 mg, 0.054 mmol) without further purification.

Procedure with thionyl chloride in DCM. The acid **6** (27 mg, 0.103 mmol, 1 equiv.) was added in a round bottom flask equipped with a condenser under nitrogen atmosphere. Anhydrous DCM was added (1 mL) and the mixture was stirred and sonicated for 5 minutes. To the resulting suspension, thionyl chloride (90 µL, 1.03 mmol, 10 equiv.) was slowly dropped at r.t. Then the mixture was refluxed at 40°C for 6 hours. Afterwards DCM and residual thionyl chloride were removed under vacuum. In the same Schlenk, the thus obtained white solid, was then dissolved at 0°C in anhydrous DMF (0.5 mL) and pyridine (8.4 µL, 0.103 mmol, 1 equiv.), and finally the amine **9** (20 mg, 0.0515 mmol, 0.5 equiv.), dissolved in anhydrous DMF (0.25 mL), were dropped. After 10 minutes at 0°C, the mixture was stirred overnight at r.t.. At competition (TLC monitoring) the mixture was diluted with EtOAc (5 mL) and quenched with water (5 mL). After 3 extractions with EtOAc (3x 5 mL), the collected organic layers were dried on Na_2SO_4, filtered and the solvent removed under reduced pressure. The crude was purified by washing with hot methanol (5 mL) and filtration on a paper filter. The product **10** was obtained as a yellow solid in 63% yield (20.5 mg, 0.0326 mmol). ^{1}H NMR (600 MHz, DMSO-d_6): δ 11.83 (s, 1H), 10.42 (s, 1H), 8.68 (d, J = 9.7 Hz, 1H), 8.51 (d, J = 7.3 Hz, 1H), 8.47 (d, J = 8.2 Hz, 1H), 8.38 (t, *J* = 5.7 Hz, 1H), 8.29 (d, *J* = 8.2 Hz, 1H), 8.01 (d, *J* = 6.7 Hz, 1H), 7.89 (dd, *J* = 8.5, 7.2 Hz, 1H), 7.38 – 7.27 (m, 5H), 5.01 (s, 2H), 4.30 (s, 2H), 4.10 (t, *J* = 6.9 Hz, 2H), 3.46 (q, *J* = 6.4 Hz, 2H), 2.79 (t, *J* = 6.8 Hz, 2H), 2.47 (t, *J* = 7.3 Hz, 2H), 1.96 (p, *J* = 7.1 Hz, 2H). ^{19}F NMR (565 MHz, DMSO-d_6): δ -170.68 (d, *J* = 6.7 Hz). ^{13}C{^{1}H} NMR (151 MHz, DMSO-d_6): δ 172.3, 170.6, 166.6, 163.6, 163.1, 157.5 (d, *J* = 25.9 Hz), 149.6, 140.1, 139.2 (d, *J* = 228.2 Hz), 136.1, 131.4, 131.0 (d, *J* = 33.7 Hz), 130.8, 129.3, 128.3, 127.9, 127.8, 126.3, 124.2, 122.3, 117.7, 65.3, 49.5, 39.0, 36.0, 35.1, 31.1, 22.8. HPLC-MS (ESI$^+$): t_R=7.32 min; m/z = 652.2 $[M+Na]^+$, 668.2 $[M+K]^+$.

4-(6-(3-(2-(5-Fluoro-2,4-dioxo-3,4-dihydropyrimidin-1(2H)-yl)acetamido)propanamido)-1,3-dioxo-1H-benzo[de]isoquinolin-2(3H)-yl)butanoic acid (11) Following GP1, starting from **10** (11.4 mg, 0.0181 mmol) dissolved into 350 µL of anhydrous DMF with Pd/C (8 mg, 70% wt) and H_2, compound **11** was obtained as a yellow solid after trituration with pentane in 98% yield (9.6 mg, 0.0179 mmol). ^{1}H NMR (600 MHz, DMSO-d_6): δ 10.59 (s, 1H), 8.75 – 8.64 (m, 1H), 8.52 – 8.48 (m, 2H), 8.46 (d, J = 8.2 Hz, 1H), 8.28 (d, J = 8.2 Hz, 1H), 7.98 (d, J = 6.7 Hz, 1H), 7.90 – 7.80 (m, 1H), 4.29 (s, 2H), 4.06 (t, J = 7.1 Hz, 2H), 3.50 – 3.40 (m, 2H), 2.79 (t, J = 6.7 Hz, 2H), 2.20 (t, J = 7.4 Hz, 2H), 1.85 (p, J = 7.3 Hz, 2H). ^{13}C{^{1}H} NMR (151 MHz, DMSO-d_6) δ 170.6, 166.7, 163.5, 163.0, 162.2, 157.8 (d, *J* = 25.0 Hz), 149.9, 140.1, 139.3 (d, *J* = 228.6 Hz), 131.4, 131.0 (d, *J* = 34.3 Hz), 130.7, 129.4, 128.3, 126.3, 124.2, 122.2,

119.7, 117.6, 49.5, 40.0, 36.0, 35.2, 32.6, 23.6. ^{19}F NMR (565 MHz, DMSO) δ -171.0 – -171.4 (m). HPLC-MS (ESI$^+$): t_R= 2.22 min; m/z = 540.1 [M+H]$^+$, 562.2 [M+Na]$^+$.

1-((2R,3R)-2-(2-(Benzyloxy)-2-oxoethyl)-4-oxo-1-(o-tolylcarbamoyl)azetidin-3-yl)ethyl 4-(6-(3-(2-(5-fluoro-2,4-dioxo-3,4-dihydropyrimidin-1(2H)-yl)acetamido)propanamido)-1,3-dioxo-1H-benzo[de]isoquinolin-2(3H)-yl)butanoate (12) In a round bottom flask, under nitrogen atmosphere, compound **11** (7 mg, 0.013 mmol, 1 equiv.) was dissolved in anhydrous DMF (125 µL) followed by addition of anhydrous DCM (250 µL) and, at 0°C, of DMAP (3 mg, 0.026 mmol, 2 equiv.) and EDC (7.5 mg, 0.038 mmol, 3 equiv.). After 20 minutes at 0°C, compound **3** (5.2 mg, 0.013 mmol, 1 equiv.) was added. The mixture was stirred at 0°C for further 30 minutes and then overnight at r.t. The reaction was monitored by TLC, then DCM was removed under reduced pressure and after dilution with EtOAc (3 mL), water was added (5 mL). After 3 more extractions with ethyl acetate (3x10 mL) the collected organic layers were washed with 0.1 M HCl (10 mL) and with a saturated aqueous solution of $NaHCO_3$ until no more acid **11** was detected by TLC in the organic phase. The organic layer was dried with Na_2SO_4, filtered, and the solvent removed under reduced pressure and high vacuum pump. The crude was then triturated at 0°C with acetonitrile (2x0.5 mL). After supernatant removal, the product was obtained as a light-yellow solid (5.0 mg, 0.006 mmol) in 46% yield. If necessary, the compound could be further purified by flash chromatography on a short pad of silica gel (eluent DCM:MeOH 96.5:3.5 + 0.1% AcOH). ^{1}H NMR (600 MHz, DMSO-d_6) δ 11.83 (s, 1H), 10.41 (s, 1H), 8.67 (dt, J = 8.5, 1.3 Hz, 1H), 8.49 – 8.46 (m, 1H), 8.45 – 8.42 (m, 1H), 8.38 (t, J = 5.7 Hz, 1H), 8.27 (d, J = 8.2 Hz, 1H), 8.01 (d, J = 6.7 Hz, 1H), 7.85 (ddd, J = 8.5, 7.2, 2.8 Hz, 1H), 7.69 (dd, J = 8.1, 1.3 Hz, 1H), 7.35 – 7.23 (m, 5H), 7.10 (ddd, J = 15.6, 7.9, 1.6 Hz, 2H), 6.96 (td, J = 7.4, 1.3 Hz, 1H), 5.26 (p, J = 6.4 Hz, 1H), 5.06 (s, 2H), 4.38 (ddd, J = 7.4, 4.4, 2.8 Hz, 1H), 4.29 (d, J = 1.5 Hz, 2H), 4.08 – 4.01 (m, 2H), 3.57 (dd, J = 6.2, 2.8 Hz, 1H), 3.46 (q, J = 6.5 Hz, 2H), 3.12 (dd, J = 16.1, 4.4 Hz, 1H), 3.02 (dd, J = 16.1, 7.6 Hz, 1H), 2.79 (t, J = 6.7 Hz, 2H), 2.40 – 2.37 (m, 2H), 2.14 (s, 3H), 1.93 – 1.88 (m, 2H), 1.25 (d, J = 6.5 Hz, 3H). ^{13}C{^{1}H} NMR (151 MHz, DMSO-d_6) δ 171.5, 170.6, 169.6, 166.7, 166.4, 163.6, 163.0, 157.6 (d, *J* = 25.7 Hz), 152.1, 149.7, 147.4, 140.1, 139.3 (d, *J* = 228.1 Hz), 135.8, 135.3, 131.5, 131.1 (d, *J* = 33.8 Hz), 130.9, 130.8, 130.2, 129.4, 128.3, 128.0, 128.0, 126.4, 126.3, 124.3, 124.2, 122.2, 121.1, 119.7, 117.6, 66.7, 65.8, 59.1, 50.9, 49.6, 38.8, 36.1, 35.8, 35.1, 31.3, 22.9, 17.8, 17.1. ^{19}F NMR (565 MHz, DMSO-d_6): -170.68 (d, J = 6.5 Hz). HPLC-MS (ESI$^+$): t_R= 8.72 min; m/z = 918.3 [M+H]$^+$, 940.3 [M+Na]$^+$. Purity (UPLC, λ=254 nm)=90%.

2-((2R,3R)-3-(1-((4-(6-(3-(2-(5-Fluoro-2,4-dioxo-3,4-dihydropyrimidin-1(2H)-yl)acetamido)propanamido)-1,3-dioxo-1H-benzo[de]isoquinolin-2(3H)-yl)butanoyl)oxy)ethyl)-4-oxo-1-(o-tolylcarbamoyl)azetidin-2-yl)acetic acid (D) Following the general procedure GP1, compound **D** was obtained starting from **11** (4.5 mg, 0.0049 mmol) and 70% wt Pd/C (3 mg), in anhydrous DMF (250 µL) and H_2 in 86% yield (3.5 mg, 0.0042 mmol) as a light yellow solid). ^{1}H NMR (600 MHz, DMSO) δ 10.5 (s, 1H), 8.7 (dd, *J* = 8.5, 1.1 Hz, 1H), 8.5 (dd, *J* = 7.3, 1.0 Hz, 1H), 8.5 (d, *J* = 8.2 Hz, 1H), 8.4 (t, *J* = 5.6 Hz, 1H), 8.3 (d, *J* = 8.2 Hz, 1H), 8.0 (d, *J* = 6.7 Hz, 1H), 7.9 (dd, *J* = 8.5, 7.2 Hz, 1H), 7.6 (d, *J* = 8.0 Hz, 1H), 7.2 (d, *J* = 7.7 Hz, 1H), 7.2 (td, *J* = 7.7, 1.6 Hz, 1H), 7.0 (td, *J* = 7.4, 1.3 Hz, 1H), 6.2 (q, *J* = 7.1 Hz, 1H), 4.8 – 4.7 (m, 1H), 4.3 (s, 2H), 4.1 (t, *J* = 7.0 Hz, 2H), 3.5 (q, *J* = 6.4 Hz, 2H), 2.8 (t, *J* = 6.7 Hz, 2H), 2.7 (dd, *J* = 16.5, 6.7 Hz, 1H), 2.5 (dd, *J* = 16.6, 5.7 Hz, 1H), 2.3 (t, *J* = 7.3 Hz, 2H), 2.2 (s, 3H), 2.0 (dd, *J* = 7.3, 1.2 Hz, 3H), 1.9 – 1.8 (m, 3H). ^{13}C{^{1}H} NMR (151 MHz, DMSO-d_6) δ 174.4, 172.6, 170.7, 166.7, 163.6, 163.1, 162.3, 157.8 (d, *J* = 25.0 Hz), 149.8, 149.0, 140.2, 140.1 (d, *J* = 229.0 Hz), 139.5, 136.2, 131.5, 131.1 (d, *J* = 34.3 Hz), 130.8, 130.2, 129.5, 128.4, 126.4, 126.0, 124.3, 124.3, 123.2,

122.3, 119.9, 117.8, 58.4, 49.6, 42.1, 39.9, 36.1, 35.2, 32.1, 23.4,17.6, 14.8. ^{19}F NMR (565 MHz, DMSO-d_6): -170.64 (d, J = 6.6 Hz). HPLC-MS (ESI$^+$): t_R= 6.08 min; m/z = 828.2 $[M+H]^+$, 850.3 $[M+Na]^+$. Purity (UPLC, λ=254 nm)=95.2%.

3-(5,5-Difluoro-1,3,7,9-tetramethyl-5H-4l4,5l4-dipyrrolo[1,2-c:2',1'-f][1,3,2]diazaborinin-10-yl)propanoic acid (13) In a round bottom flask under nitrogen atmosphere, 2,4-dimethylpirrole (120 µL, 1.2 mmol, 2 equiv.) was dissolved into 10 mL of anhydrous DCM. Then succinic anhydride (60 mg, 0.6 mmol, 1 equiv.), boron trifluoride diethyl etherate (0.5 mL, 4 mmol, 6.6 equiv.) and TEA (0.42 mL, 3 mmol, 5 equiv.) were added. The reaction mixture was left under stirring under reflux for 5 hours and monitored by TLC. At completion, the reaction was quenched with water and extracted with DCM (3x10 mL). The collected organic layers were dried on Na_2SO_4, filtered and the solvent removed under reduced pressure. The crude was purified by flash-chromatography on silica gel (from 50:50 = Ciclohexane: AcOEt to 0: 100 = CicloHex : AcOEt + 0.1% Acetic acid). The product **13** was obtained as an orange solid in 20% yield (40 mg, 0.125 mmol). ^1H NMR (400 MHz, $CDCl_3$) δ 6.08 (s, 2H), 3.37 – 3.29 (m, 2H), 2.69 – 2.63 (m, 2H), 2.52 (s, 6H), 2.45 (s, 6H HPLC-MS (ESI$^+$): t_R=6.0 min; m/z = 301.1 $[M-F]^+$, 321.1 $[M+H]^+$, 623.3 $[2M+Na]^+$.The spectroscopic data are consistent with those reported in the literature: ref: Yanai, H., Hoshikawa, S., Moriiwa, Y., Shoji, A., Yanagida, A., and Matsumoto, T. (2021) A fluorinated carbanionic substituent for improving water solubility and lipophilicity of fluorescent dyes. *Angew. Chem. Int. Ed. 60,* 5168-5172. DOI: 10.1002/anie.202012764.

(R)-1-((2R,3S)-2-(2-(benzyloxy)-2-oxoethyl)-4-oxo-1-(o-tolylcarbamoyl)azetidin-3-yl)ethyl 3-(5,5-difluoro-1,3,7,9-tetramethyl-5H-4l4,5l4-dipyrrolo[1,2-c:2',1'-f][1,3,2]diazaborinin-10-yl)propanoate (14) In a round bottom flask under nitrogen atmosphere, compound **13** (10 mg, 0.0313 mmol, 1 equiv.) dissolved in 0.5 mL of anhydrous DCM was introduced. At 0°C, DMAP (2 mg, 0.0157 mmol, 0.5 equiv.) and EDC hydrochloride (9 mg, 0.047 mmol, 1.5 equiv.) were added. After 15 minutes at 0°C, compound **3** (12.5 mg, 0.313 mmol, 1 equiv.) was added and the reaction stirred at 0°C for 1 hour, then at room temperature for 2 days. At completion (TLC monitoring), the reaction was diluted with DCM (5 mL), quenched with water (5 mL) and extracted with DCM (3x10 mL). The collected organic layers were dried on Na_2SO_4, filtered and the solvent removed under reduced pressure. The crude was purified by flash chromatography on silica gel (90:10 cyclohexane: AcOEt) and the product **14** obtained as an orange solid in 36% yield (7.8 mg, 0.0112 mmol). ^1H NMR (600 MHz, $CDCl_3$): δ 8.36 (s, 1H), 7.91 – 7.87 (m, 1H), 7.33 – 7.27 (m, 5H), 7.22 – 7.16 (m, 2H), 7.05 (td, *J* = 7.5, 1.3 Hz, 1H), 6.03 (s, 2H), 5.38 (p, *J* = 6.5 Hz, 1H), 5.12 (s, 2H), 4.43 (ddd, *J* = 8.6, 3.8, 2.7 Hz, 1H), 3.38 – 3.32 (m, 2H), 3.28 (dd, *J* = 9.7, 7.8 Hz, 2H), 2.84 (dd, *J* = 16.4, 8.6 Hz, 1H), 2.62 (ddd, *J* = 16.3, 9.8, 7.8 Hz, 1H), 2.57 – 2.48 (m, 7H), 2.41 (s, 6H), 2.26 (s, 3H), 1.38 (d, *J* = 6.4 Hz, 3H). $^{13}C\{^1H\}$ NMR (151 MHz, $CDCl_3$): δ 171.0, 169.6, 166.2, 154.9, 147.6, 143.1, 140.5, 135.4, 135.2, 131.3, 130.7, 128.8, 128.6, 128.4, 127.5, 127.0, 124.8, 122.1, 121.1, 67.8, 67.0, 60.4, 51.6, 36.7, 36.5, 35.5, 23.6, 18.3, 17.8, 16.5, 14.6. ^{19}F NMR (377 MHz, $CDCl_3$): δ -146.18 – -147.0 (m, 2F). HPLC-MS (ESI$^+$): t_R= 13.9 min; m/z = 721.3 $[M+Na]^+$. ATR-FTIR (cm^{-1}): 3475, 3334, 2926, 2851,1763, 1734, 1718, 1546, 1508, 1306, 1196. Purity (UPLC, λ=254 nm)=90.4%.

2-((2R,3S)-3-((R)-1-((3-(5,5-Difluoro-1,3,7,9-tetramethyl-5H-4l4,5l4-dipyrrolo[1,2-c:2',1'-f][1,3,2]diazaborinin-10-yl)propanoyl)oxy)ethyl)-4-oxo-1-(o-tolylcarbamoyl)azetidin-2-yl)acetic acid (E) Following GP1, starting from **14** (13 mg, 0.019 mmol, 1 equiv.), in a 1:1 mixture of anhydrous

methanol and THF (3 mL total volume) with 20% wt of Pd/C (3 mg), compound **B** was obtained as an orange-red solid in 99% yield (11 mg, 0.0184 mmol). If necessary, the compound could be purified by flash chromatography on silica gel (eluent 50:50 Cyclohexane:AcOEt and then 50:50 Cyclohexane:AcOEt + 0.1% Acetic acid). ^{1}H NMR (600 MHz, $CDCl_3$): δ 8.38 (s, 1H), 7.92 – 7.85 (m, 1H), 7.23 – 7.16 (m, 2H), 7.06 (td, *J* = 7.5, 1.3 Hz, 1H), 6.04 (s, 2H), 5.34 (h, *J* = 6.6 Hz, 1H), 4.34 (ddd, *J* = 8.8, 4.1, 2.7 Hz, 1H), 3.34 – 3.23 (m, 3H), 3.20 (dd, *J* = 7.3, 2.7 Hz, 1H), 2.68 (dd, *J* = 16.5, 8.8 Hz, 1H), 2.63 – 2.55 (m, 2H), 2.49 (s, 6H), 2.40 (s, 6H), 2.28 (s, 3H), 1.38 (d, *J* = 6.4 Hz, 3H). ^{13}C{^{1}H} NMR (151 MHz, $CDCl_3$): δ 172.2, 171.0, 166.1, 154.9, 147.9, 143.2, 135.1, 131.4, 130.7, 127.7, 127.1, 125.0, 122.2, 121.3, 77.4, 77.2, 76.9, 68.0, 60.5, 51.7, 36.3, 35.5, 23.5, 18.4, 17.8, 16.5, 14.6. ^{19}F NMR (376 MHz, $CDCl_3$) δ -149.13 – -151.72 (m). ATR-FTIR (cm^{-1}): 3334, 2925, 2853, 1765, 1714, 1715, 1592, 1458, 1306,1253, 1196, 982. HPLC-MS (ESI^+): t_R=10.5 min; m/z = 589.3 $[M-F]^+$, 631 $[M+Na]^+$. Purity (UPLC, λ=254 nm)=95.9%.

3. Copies of ^{1}H, ^{13}C and ^{19}F NMR spectra

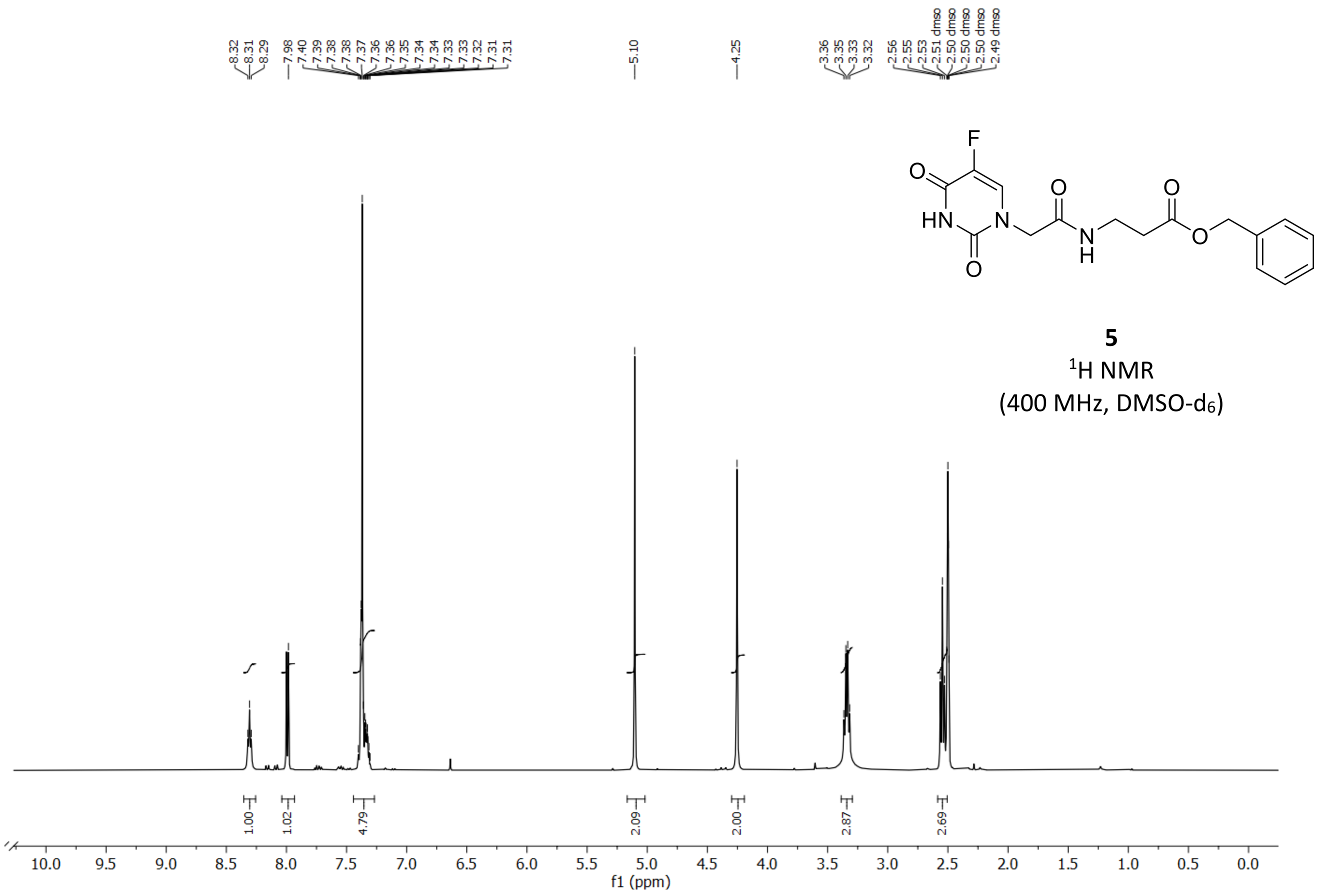

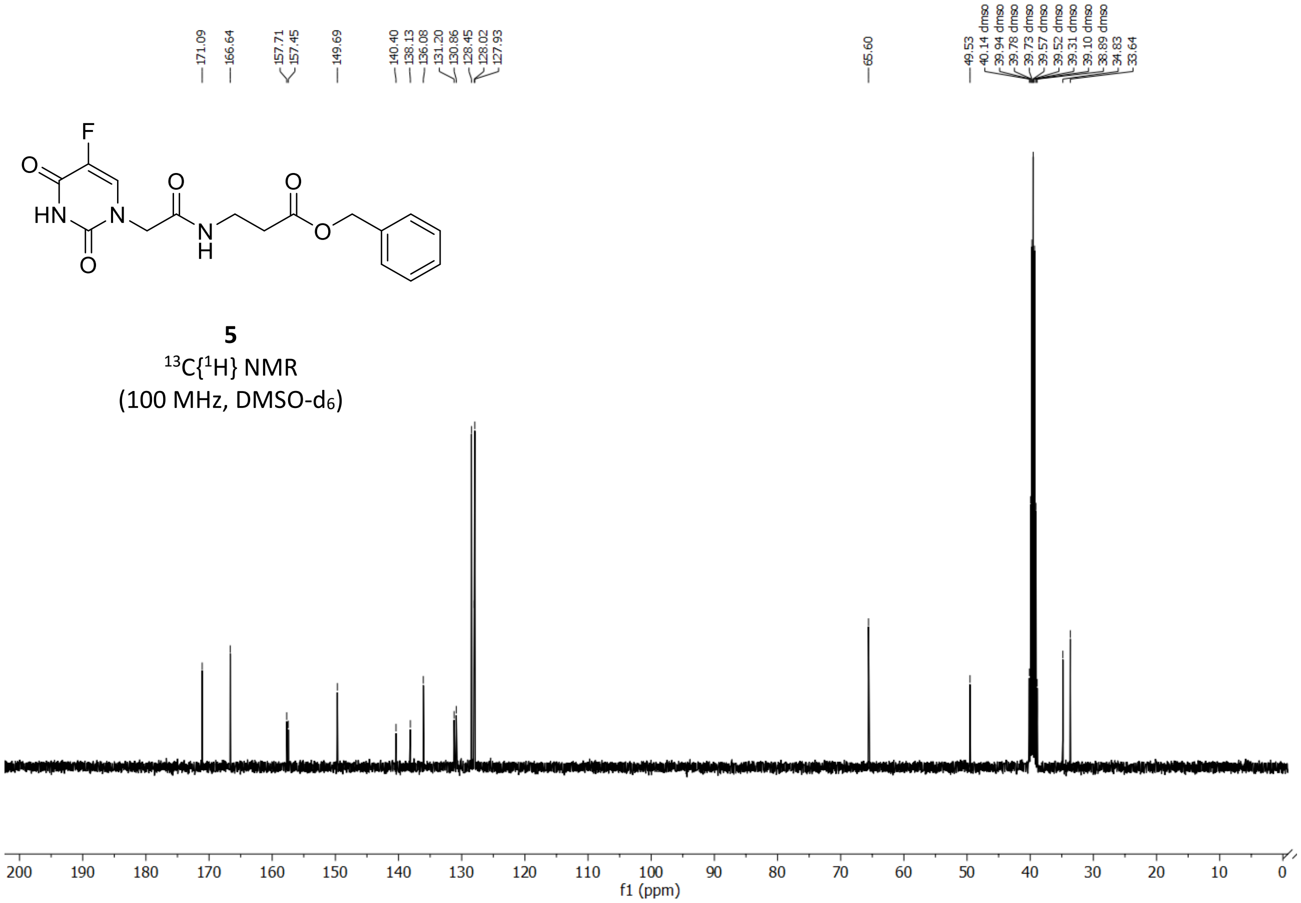

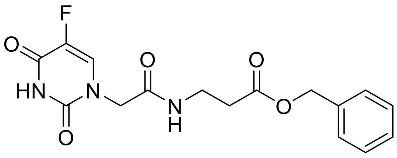

5
^{19}F NMR
(376 MHz, DMSO-d_6)

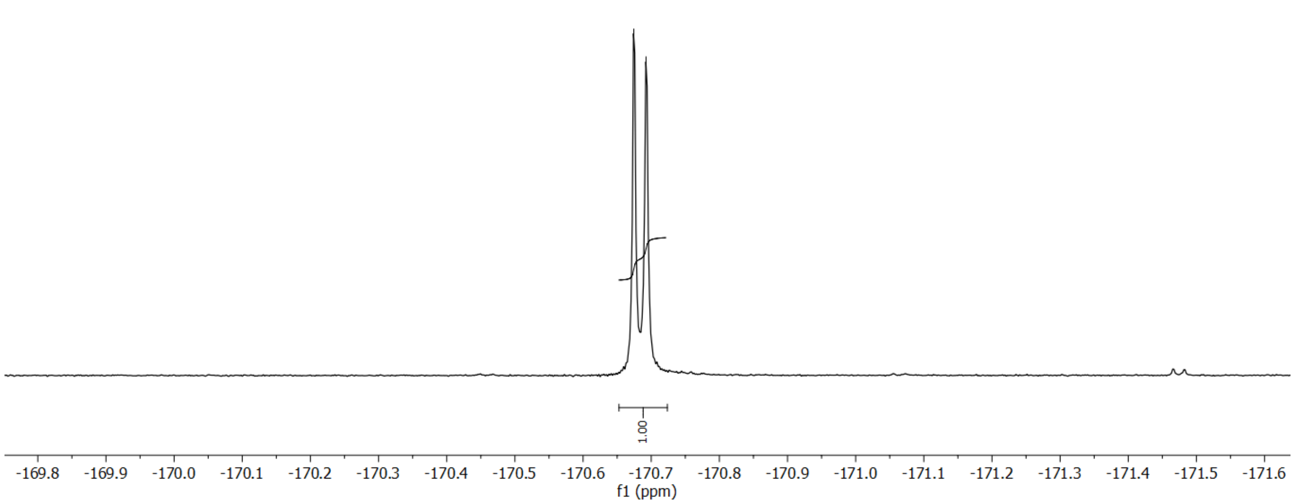

Figure S1. ^{1}H, ^{13}C and ^{19}F NMR spectra of compound **5.**

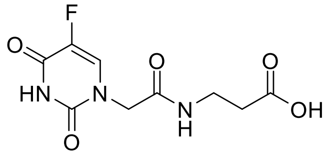

6
^{1}H NMR
(400 MHz, DMSO-d$_6$)

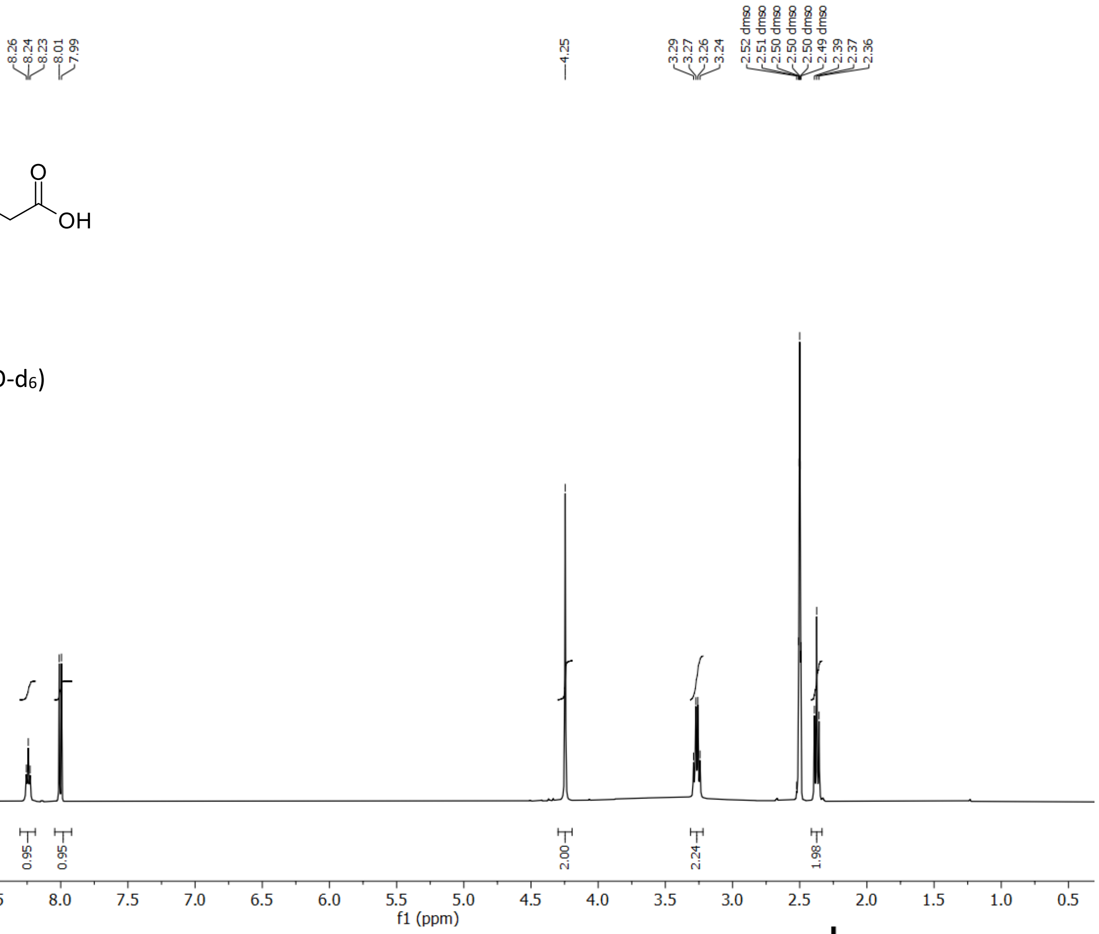

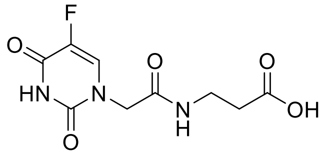

6
^{13}C{^{1}H} NMR
(100 MHz, DMSO-d$_6$)

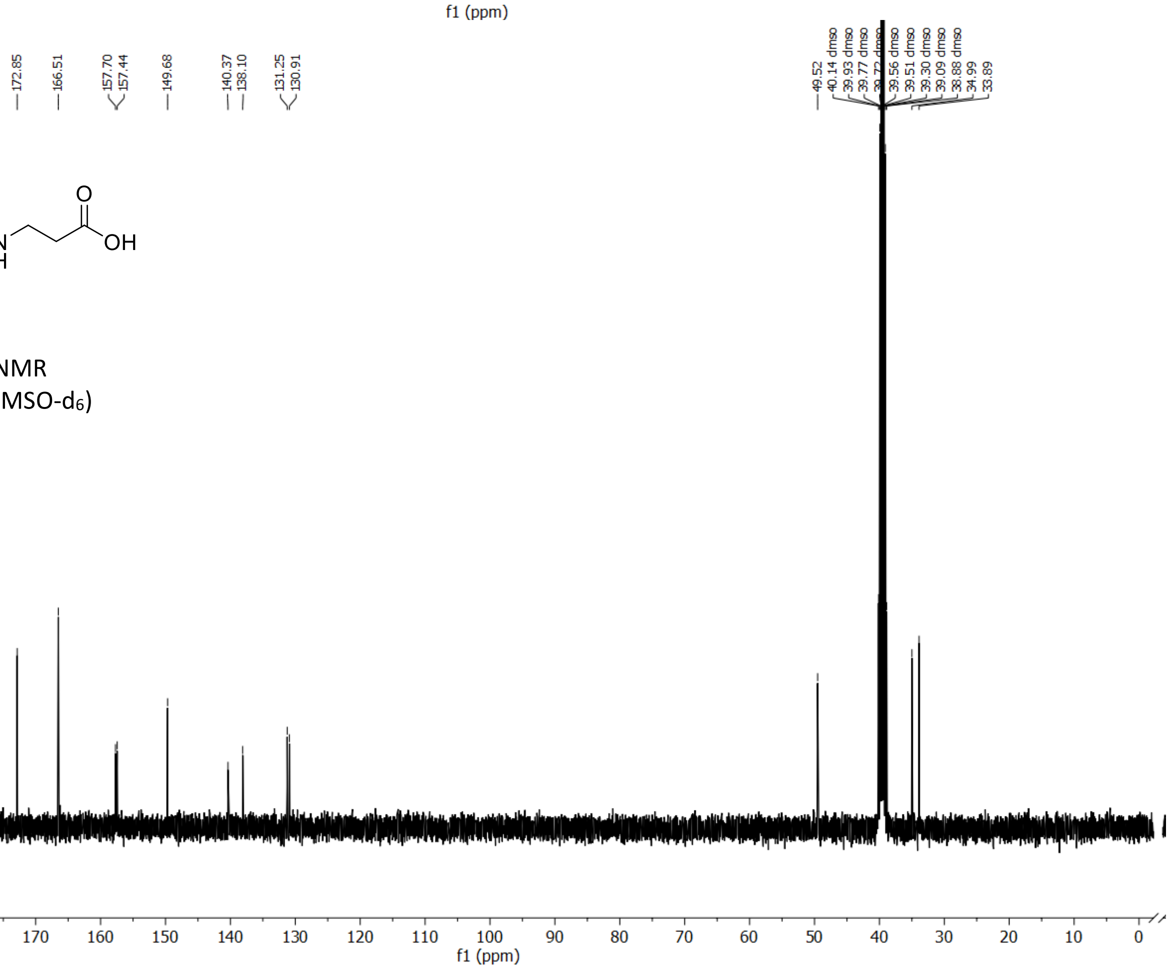

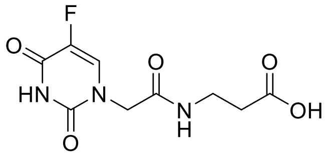

6

^{19}F NMR

(376 MHz, DMSO-d_6)

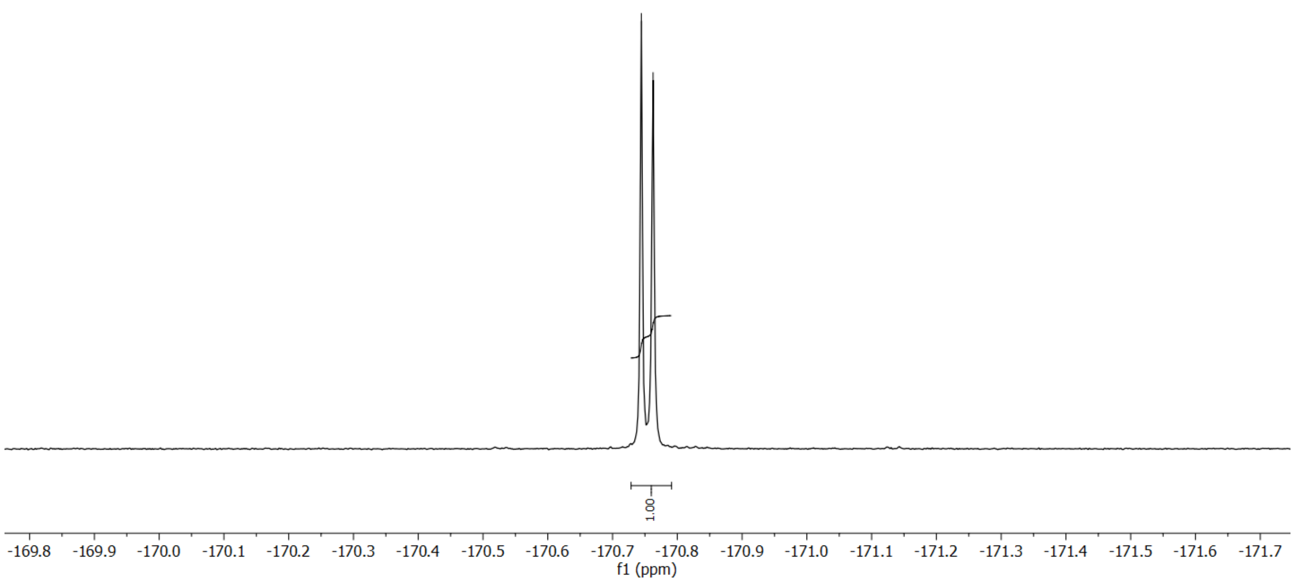

Figure S2. ^{1}H, ^{13}C and ^{19}F NMR spectra of compound **6.**

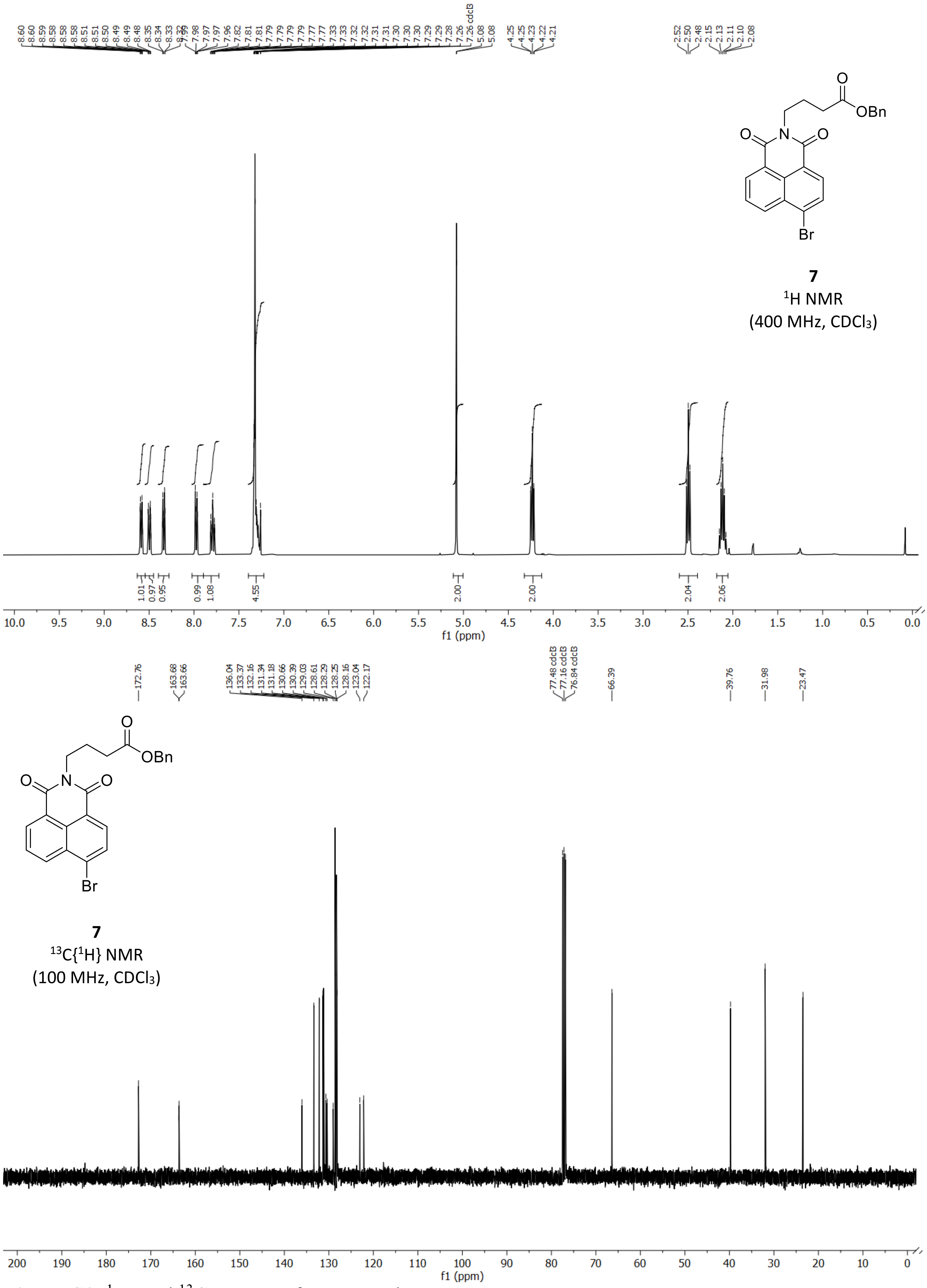

Figure S3. ^{1}H, and ^{13}C spectra of compound **7.**

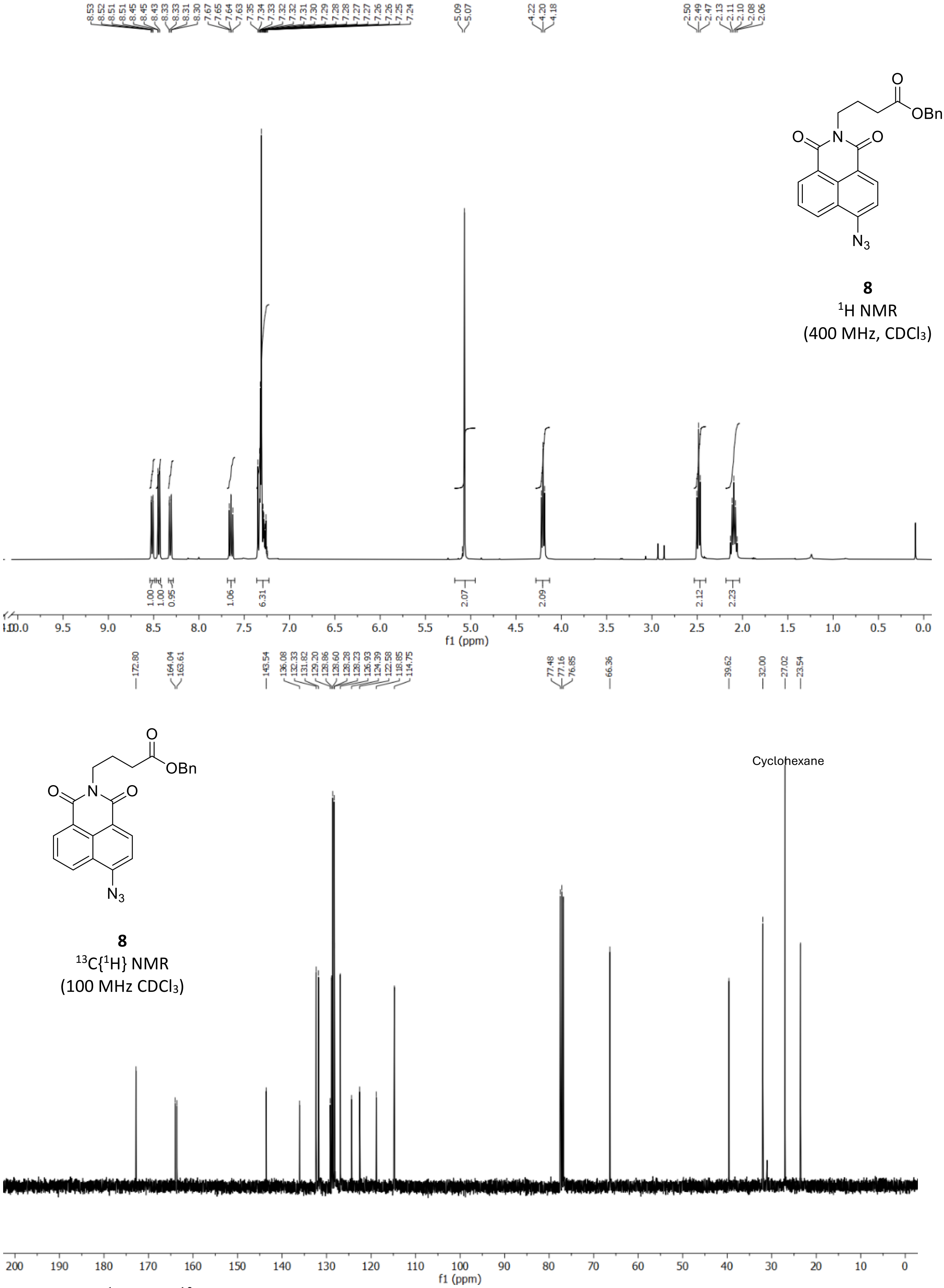

Figure S4. ^{1}H, and ^{13}C spectra of compound **8.**

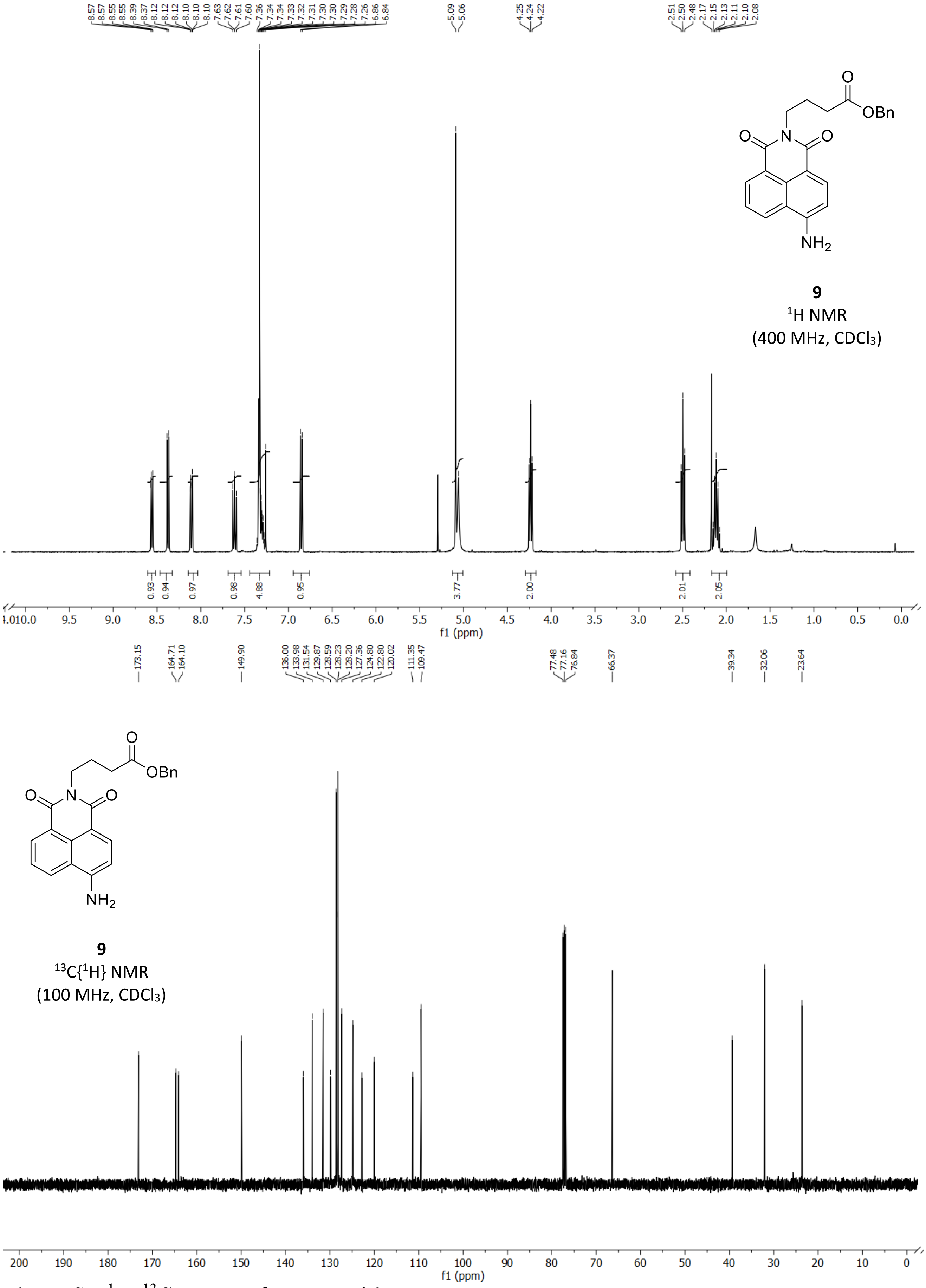

Figure S5. ^{1}H, ^{13}C spectra of compound **9.**

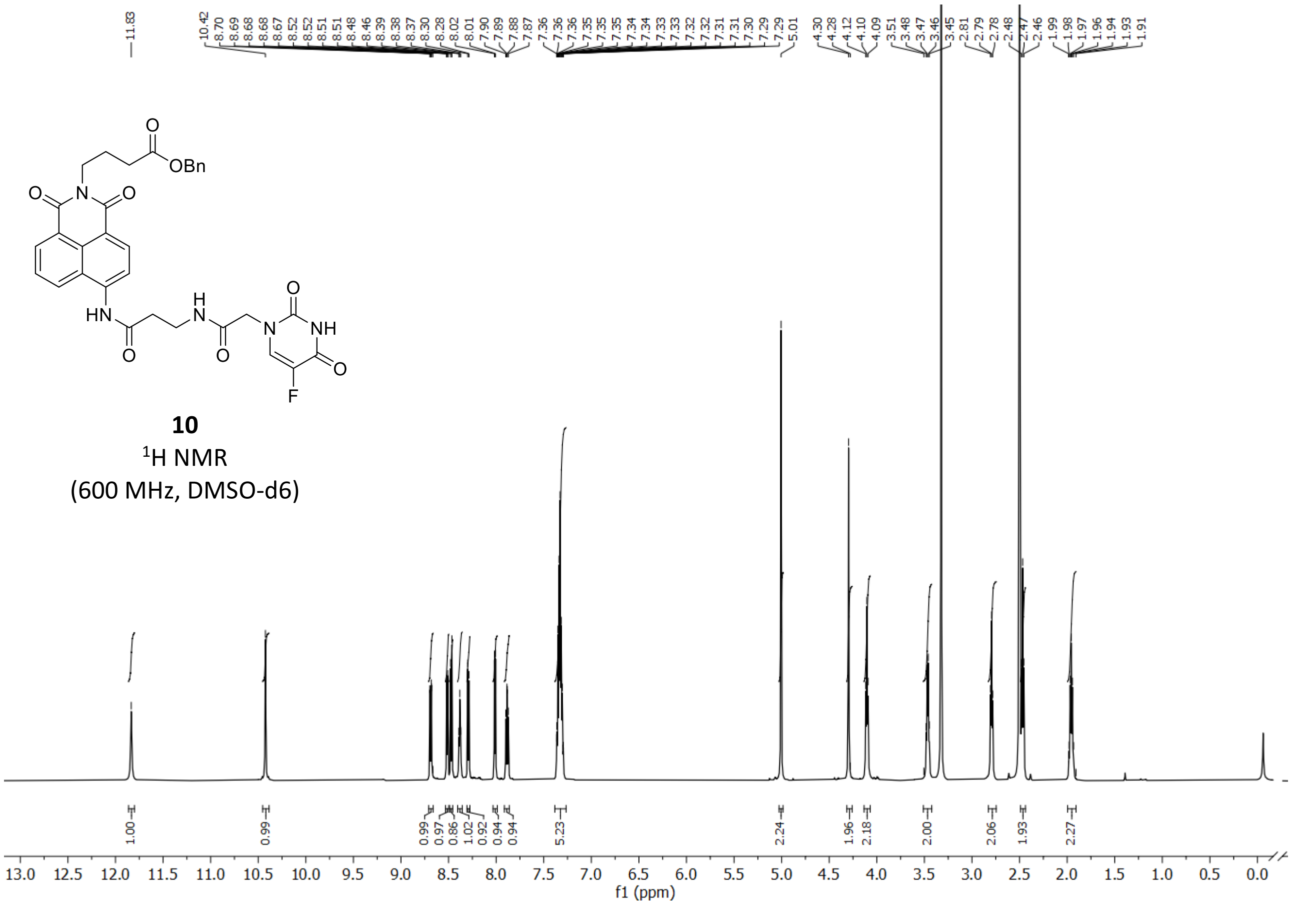

10
^{1}H NMR
(600 MHz, DMSO-d6)

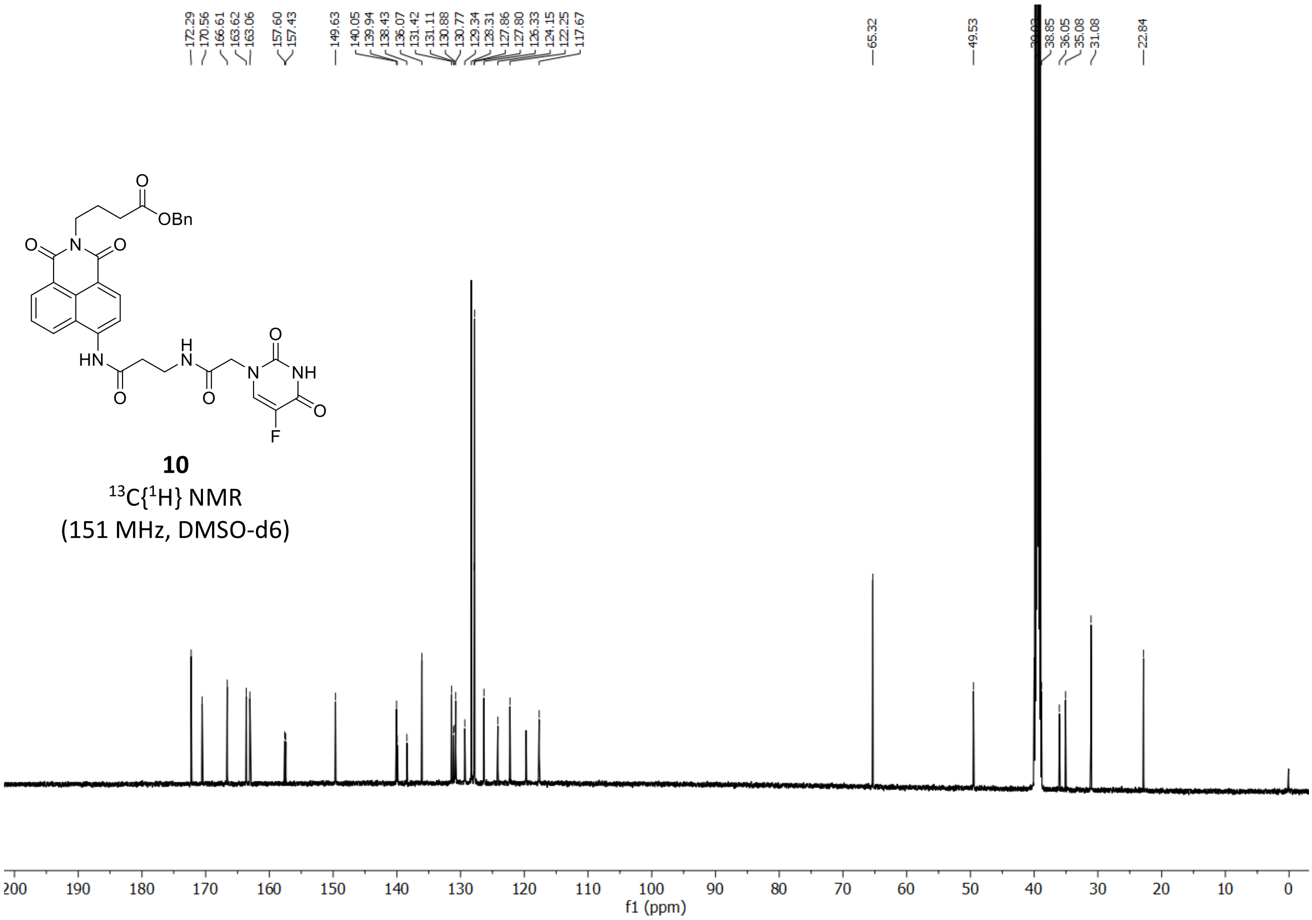

10
^{13}C{^{1}H} NMR
(151 MHz, DMSO-d6)

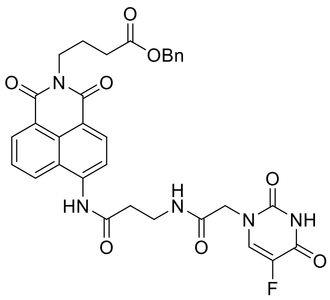

10

^{19}F NMR

(565 MHz, DMSO-d6)

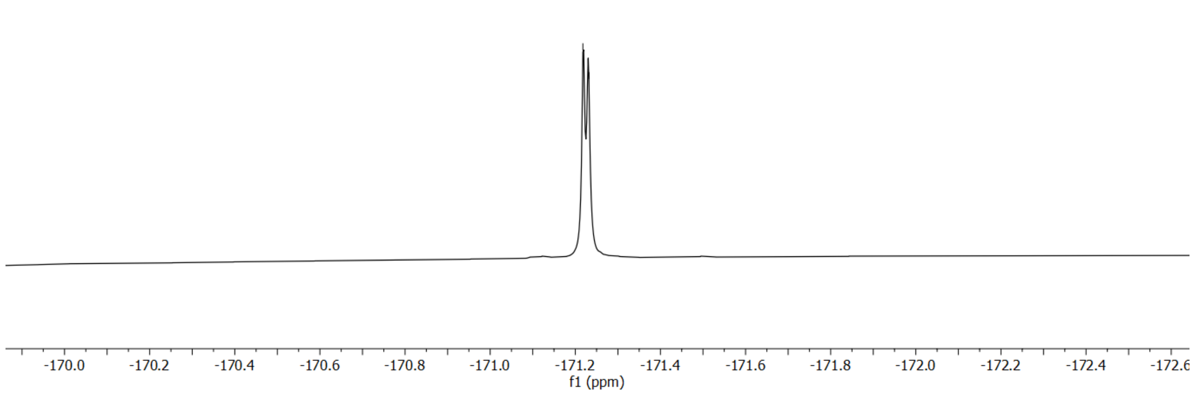

Figure S6. ^{1}H, ^{13}C and ^{19}F NMR spectra of compound **10.**

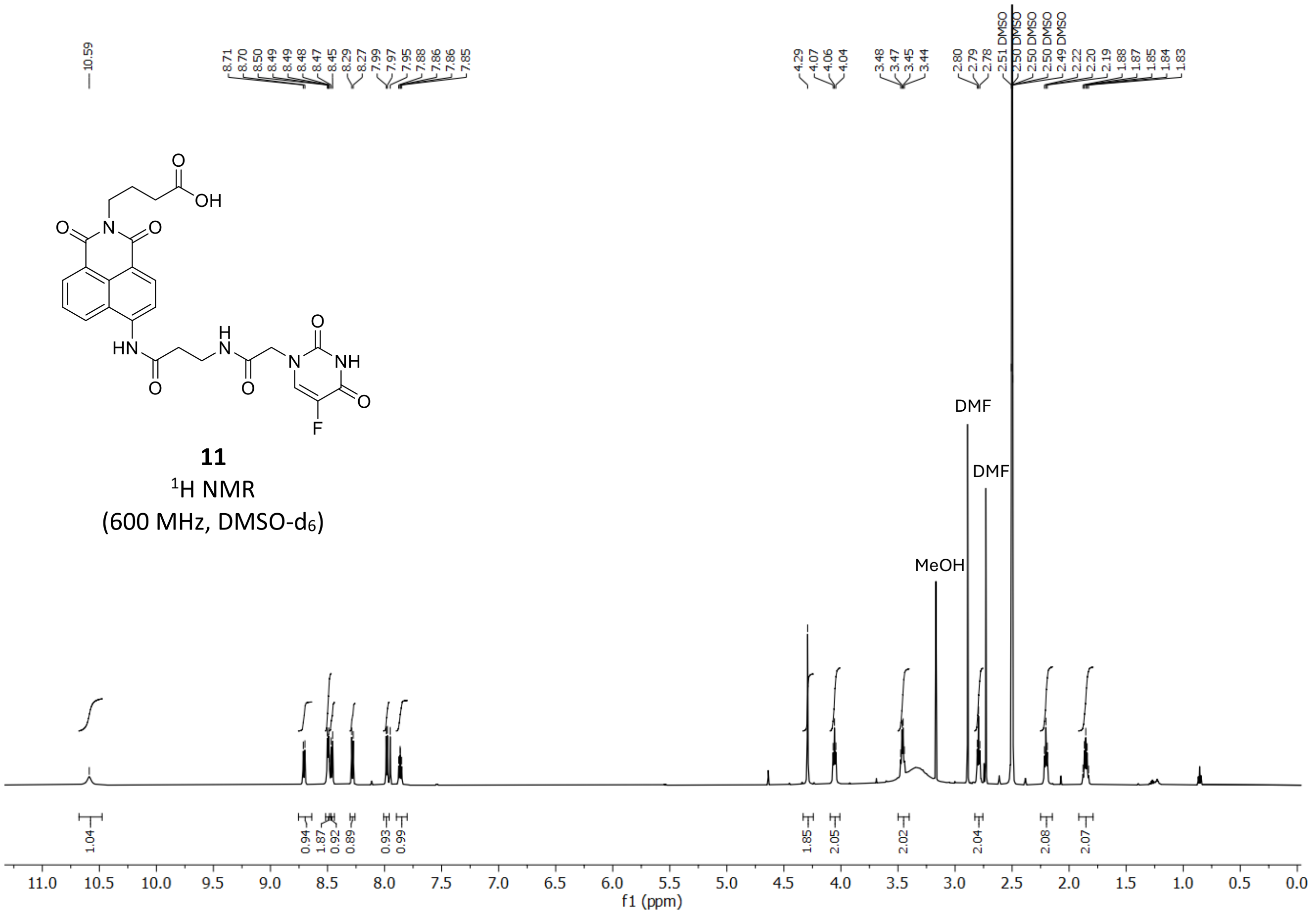
10.59
8.71 8.70 8.50 8.49 8.49 8.48 8.47 8.45 8.29 8.27 7.99 7.97 7.95 7.88 7.86 7.86 7.85
4.29 4.07 4.06 4.04
3.48 3.47 3.45 3.44
2.80 2.79 2.78 2.51 DMSO 2.50 DMSO 2.50 DMSO 2.50 DMSO 2.49 DMSO 2.22 2.20 2.19 1.88 1.87 1.85 1.84 1.83
OH
O
N
HN
H
NH
F
11
1H NMR
(600 MHz, DMSO-d6)
DMF
DMF
MeOH
1.04
0.94 1.87 0.92 0.89 0.93 0.99
1.85 2.05 2.02 2.04 2.08 2.07
f1 (ppm)

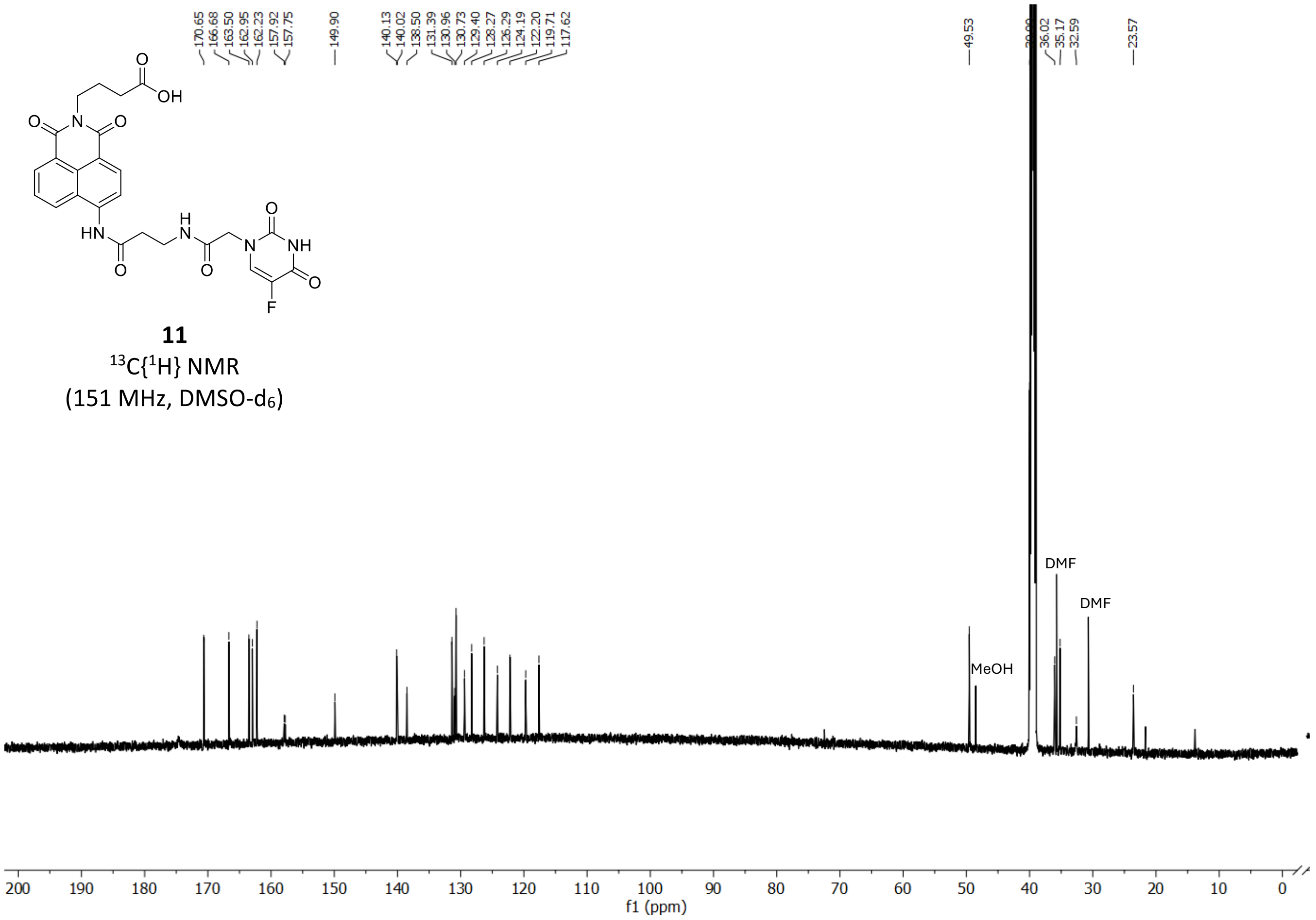
170.65 166.68 163.50 162.95 162.23 157.92 157.75
149.90
140.13 140.02 138.50 131.39 130.96 130.73 129.40 128.27 126.29 124.19 122.20 119.71 117.62
49.53
36.02 35.17 32.59
23.57
11
13C{1H} NMR
(151 MHz, DMSO-d6)
DMF
DMF
MeOH
f1 (ppm)

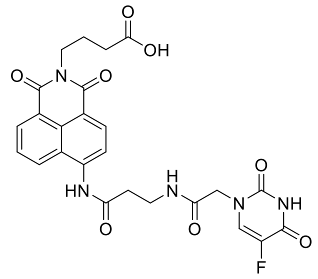

11
^{19}F NMR
(565 MHz, DMSO-d$_6$)

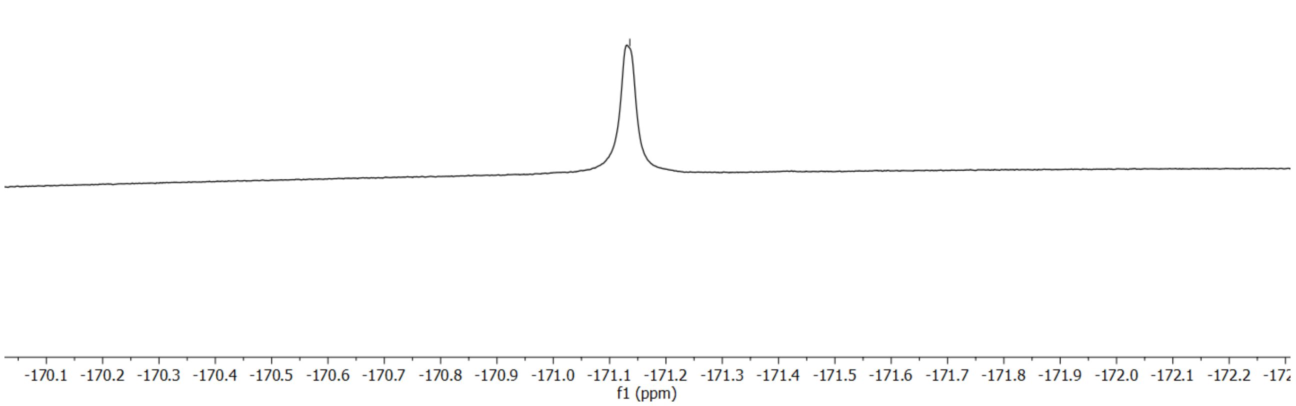

Figure S7. ^{1}H, ^{13}C and ^{19}F NMR spectra of compound **11.**

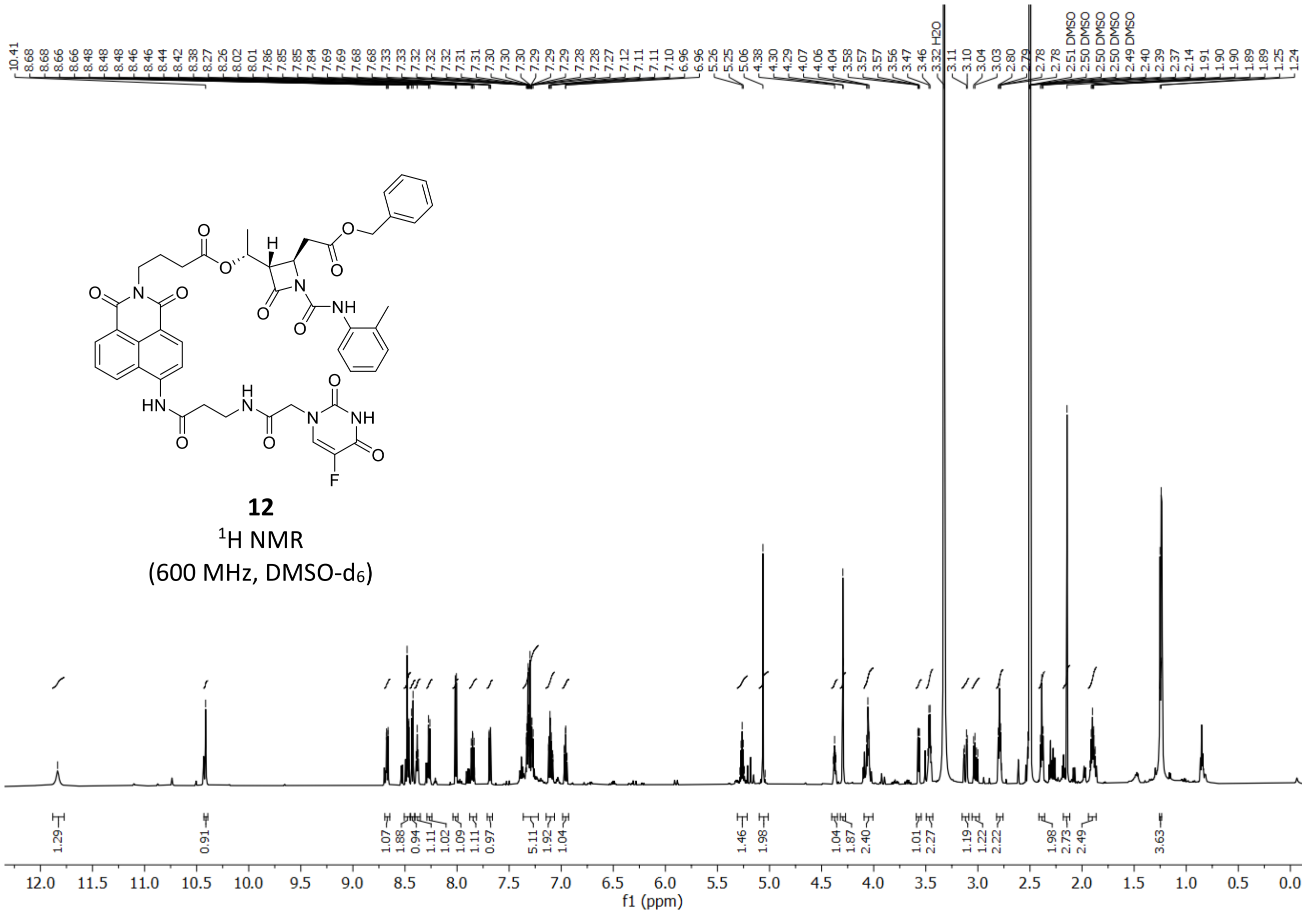

12
^{1}H NMR
(600 MHz, DMSO-d_6)

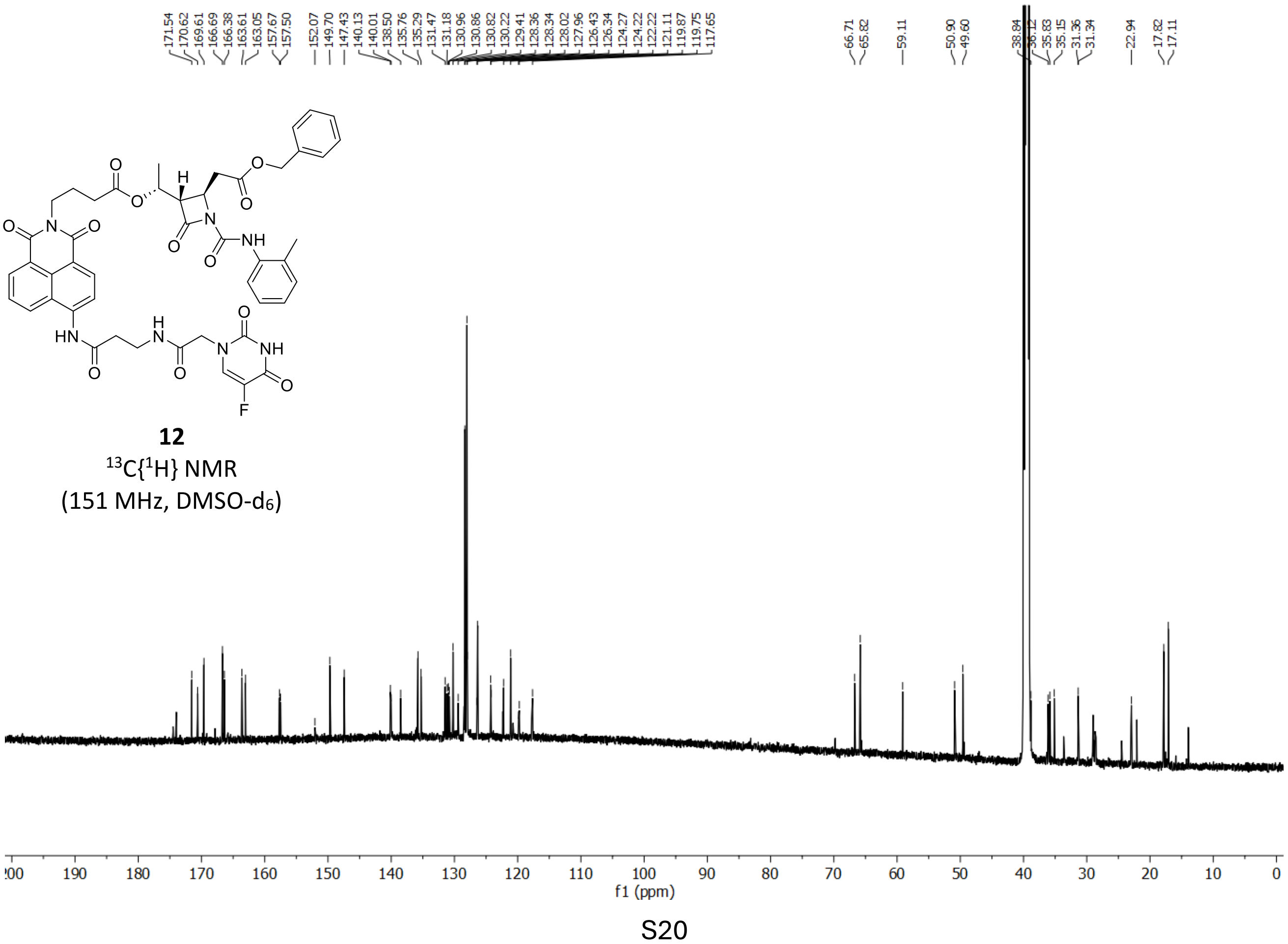

12
$^{13}C\{^{1}H\}$ NMR
(151 MHz, DMSO-d_6)

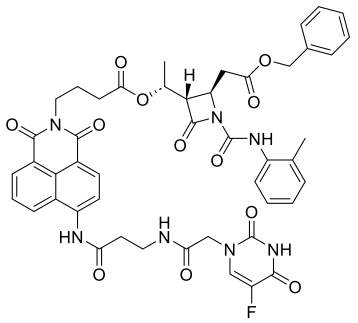

12

^{19}F NMR

(565 MHz, DMSO-d_6)

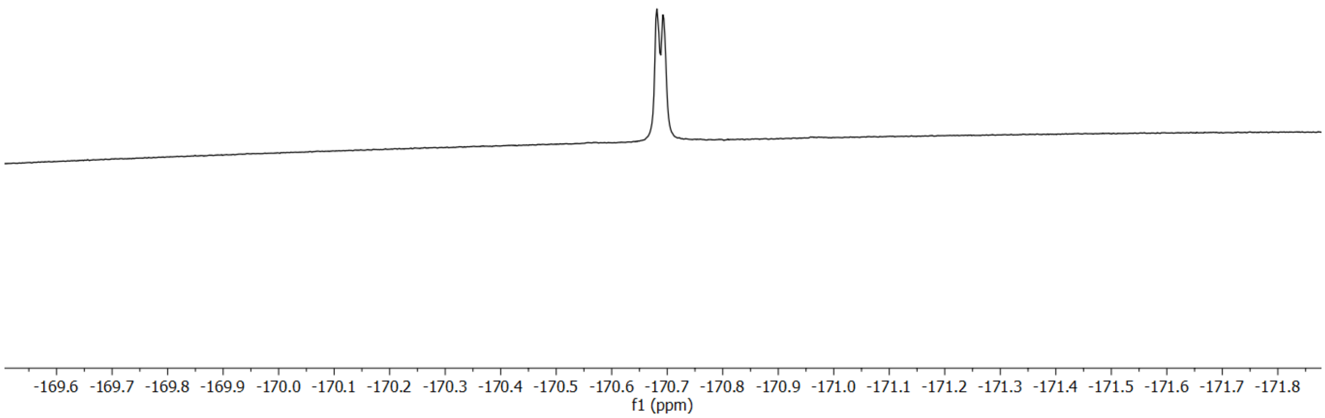

Figure S8. ^{1}H, ^{13}C and ^{19}F NMR spectra of compound **12.**

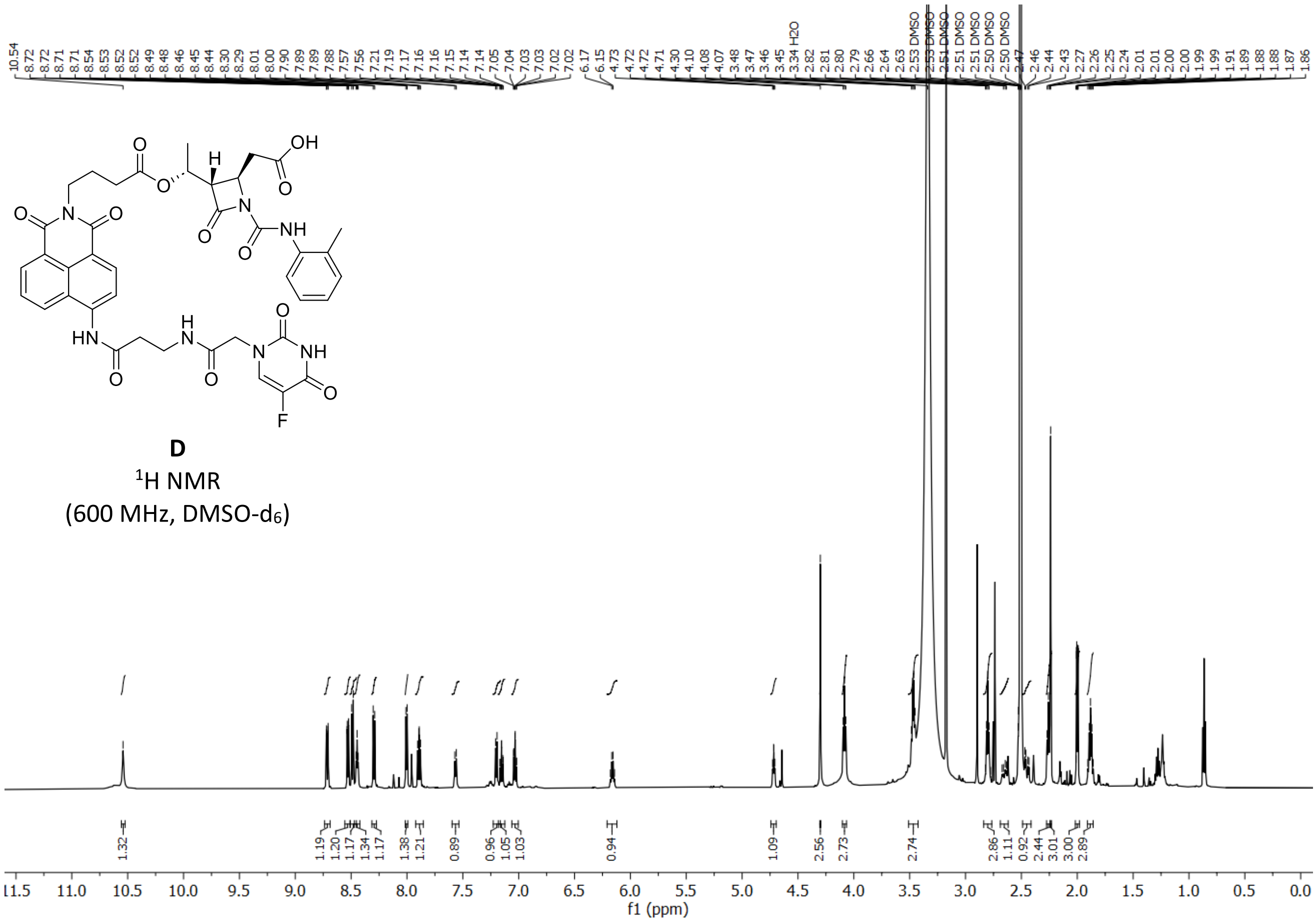

D
^{1}H NMR
(600 MHz, DMSO-d$_6$)
f1 (ppm)

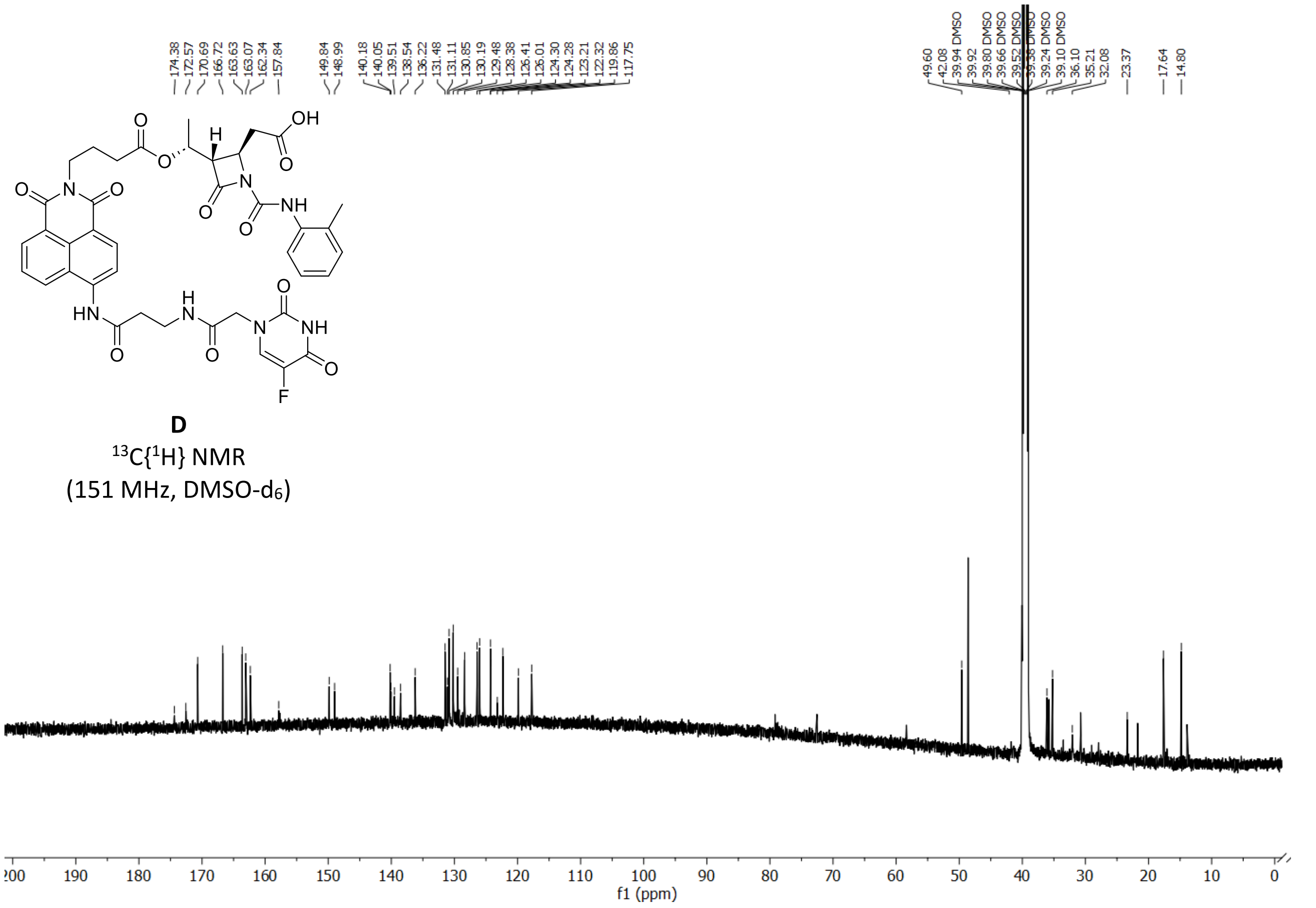

D
^{13}C{^{1}H} NMR
(151 MHz, DMSO-d$_6$)
f1 (ppm)

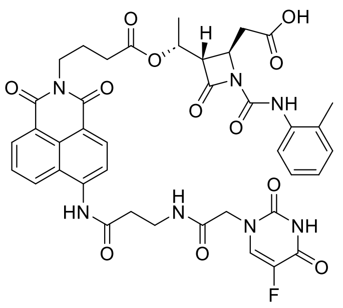

D
^{19}F NMR
(565 MHz, DMSO-d$_6$)

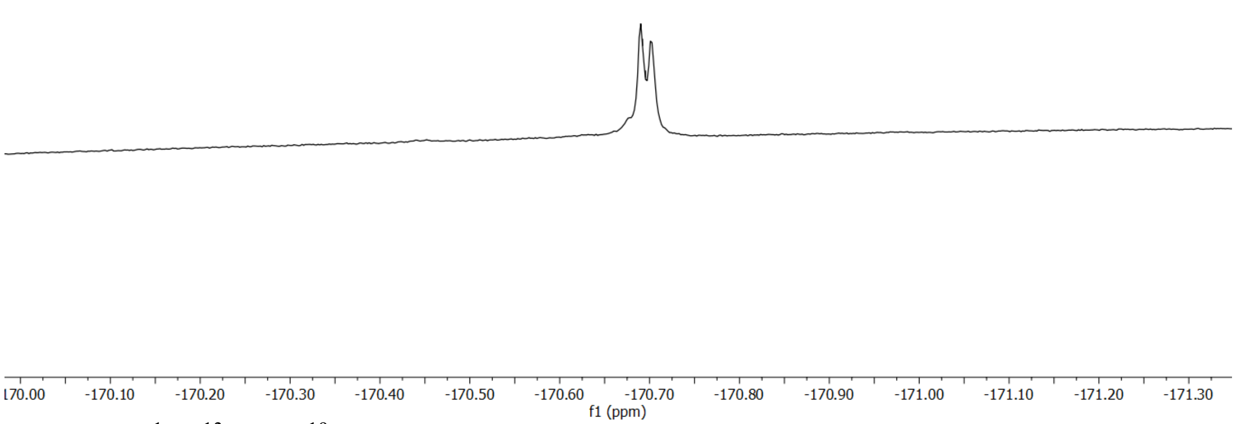

Figure S9. ^{1}H, ^{13}C and ^{19}F NMR spectra of compound **D.**

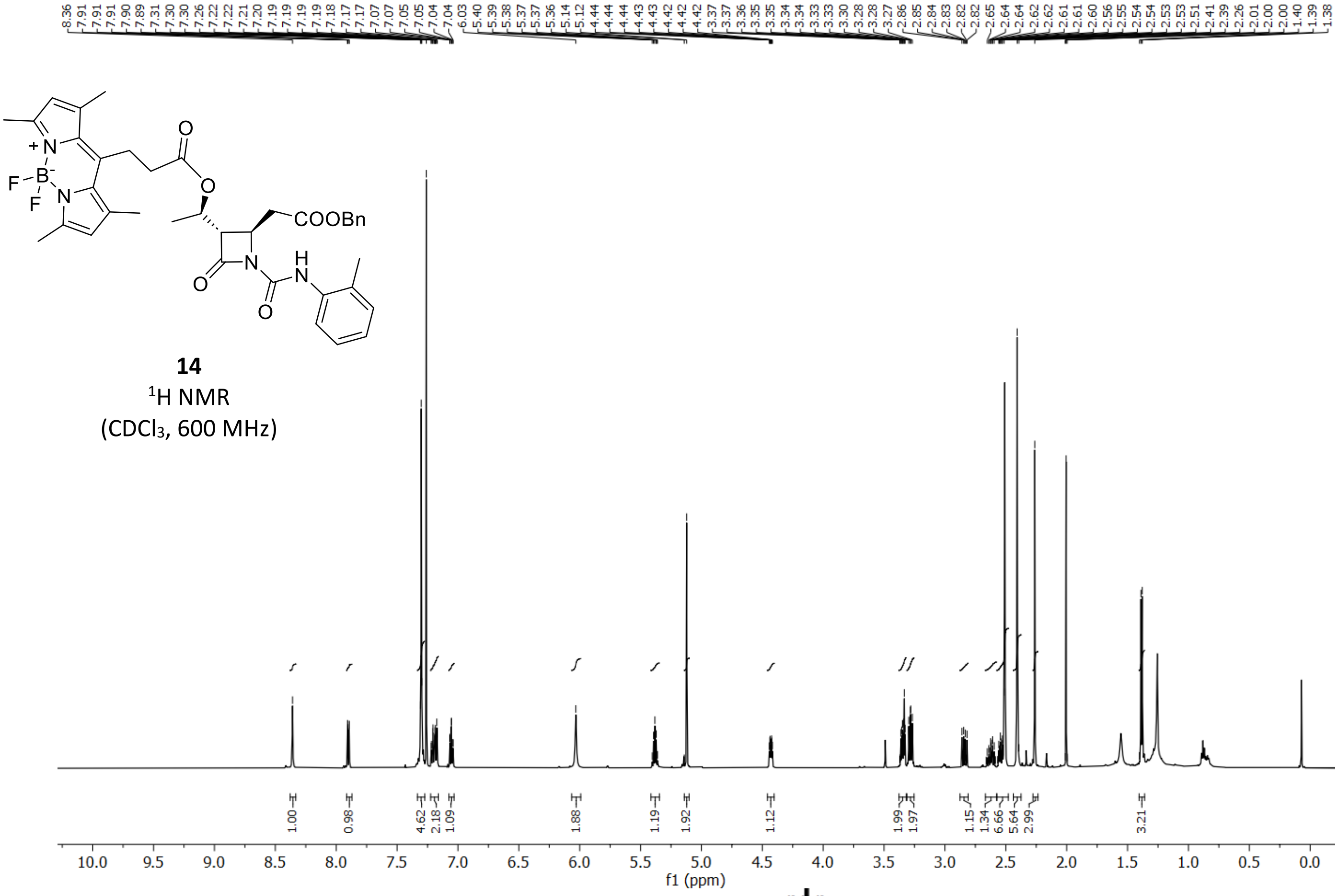

14

^{1}H NMR

($CDCl_3$, 600 MHz)

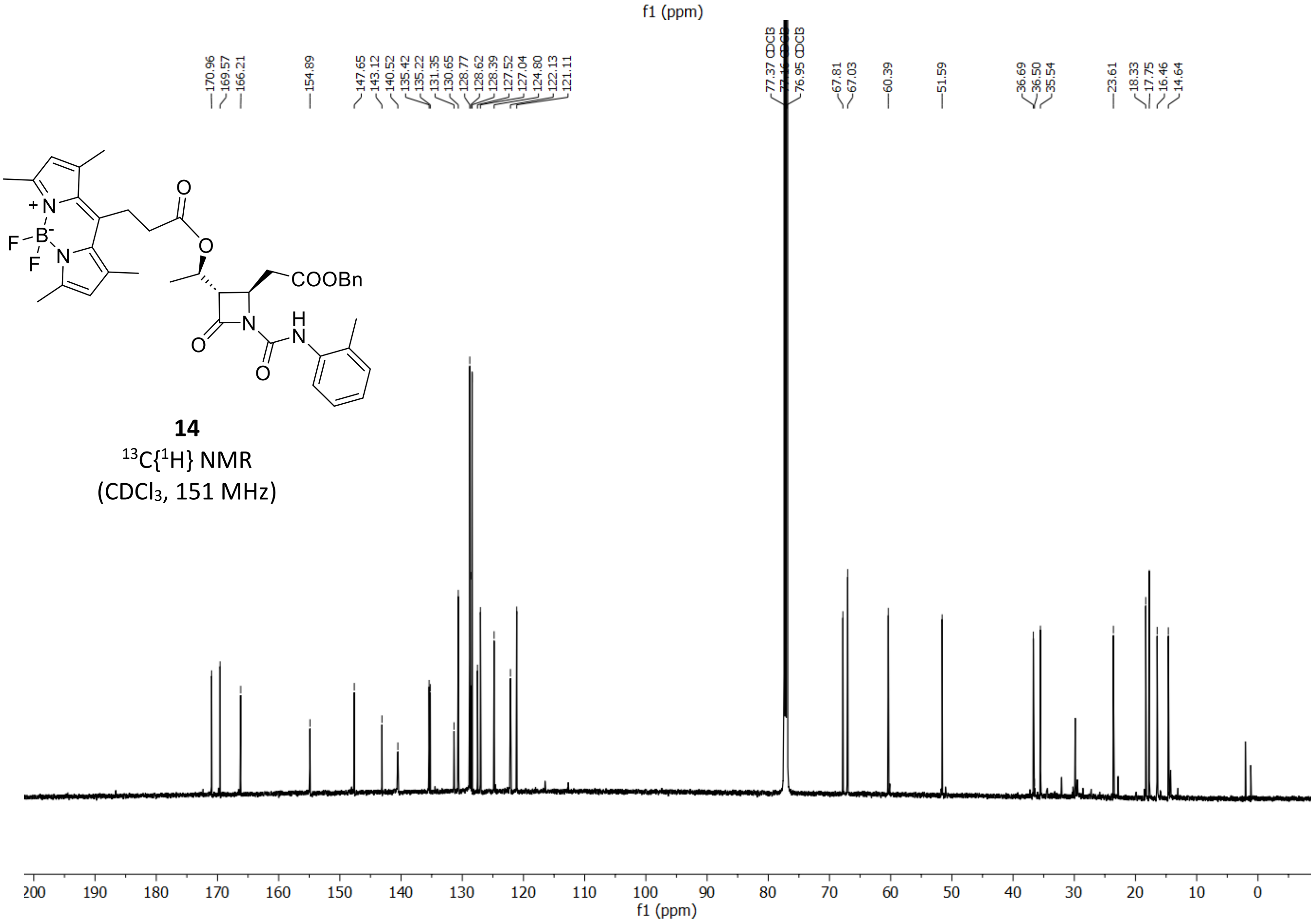

14

$^{13}C\{^{1}H\}$ NMR

($CDCl_3$, 151 MHz)

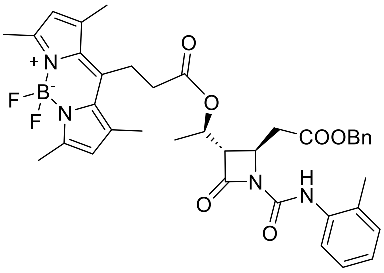

14

^{19}F NMR

($CDCl_3$, 376 MHz)

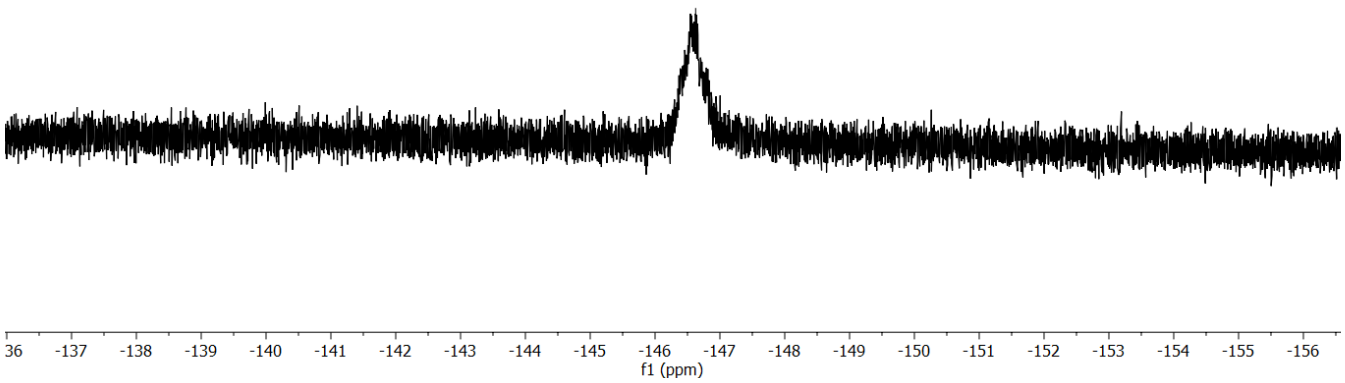

Figure S10. ^{1}H, ^{13}C and ^{19}F NMR spectra of compound **14.**

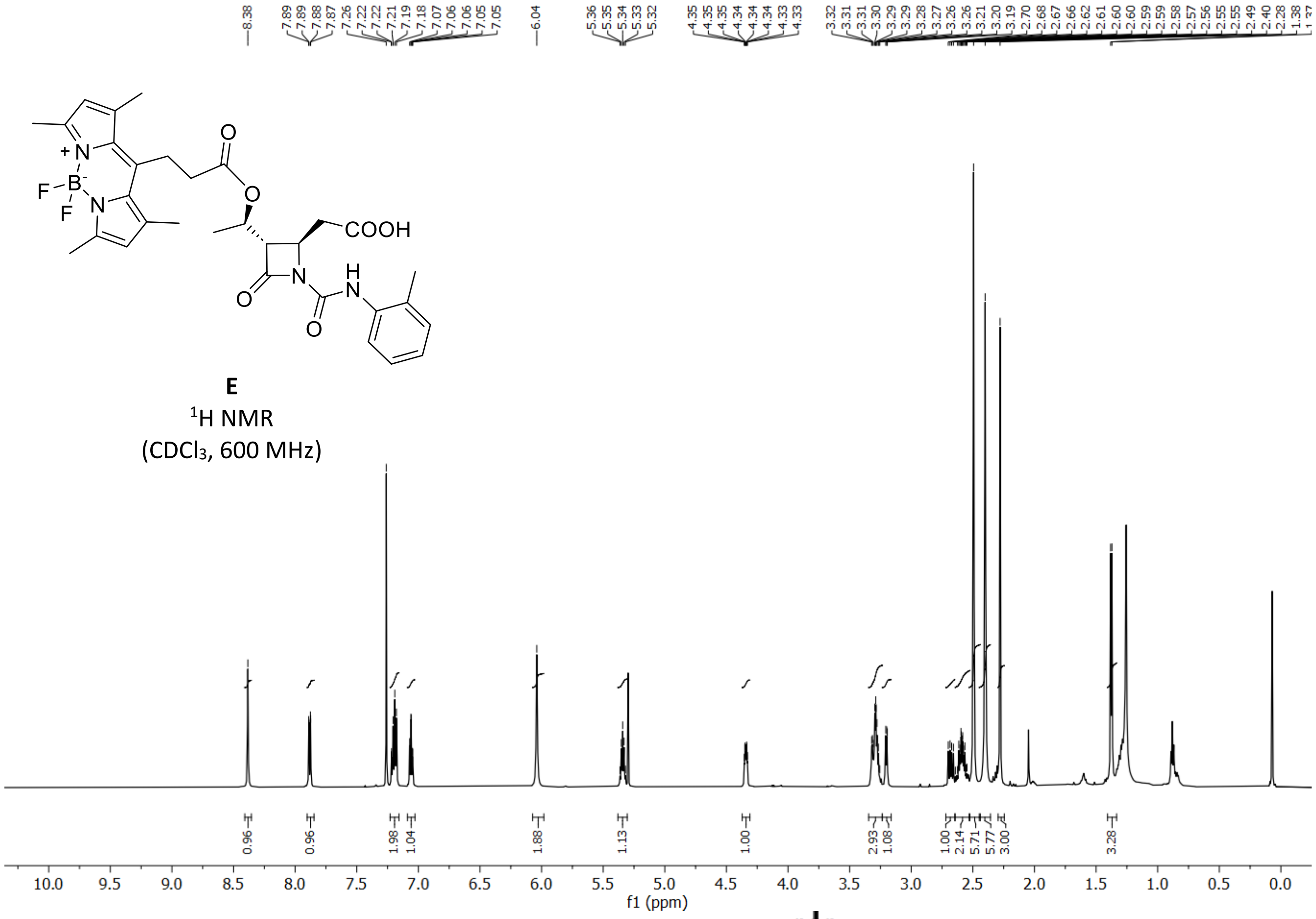

E

^{1}H NMR

($CDCl_3$, 600 MHz)

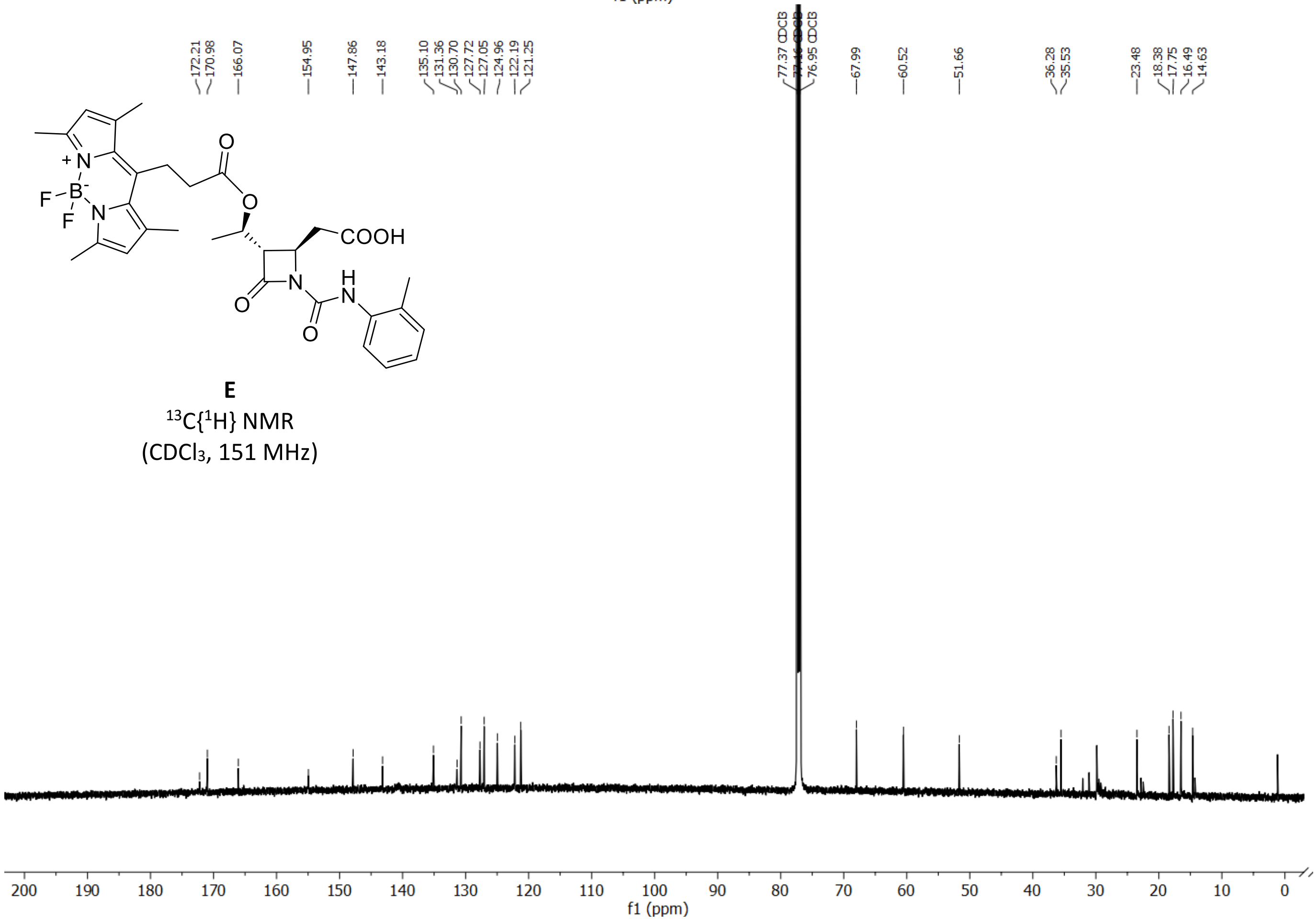

E

$^{13}C\{^1H\}$ NMR

($CDCl_3$, 151 MHz)

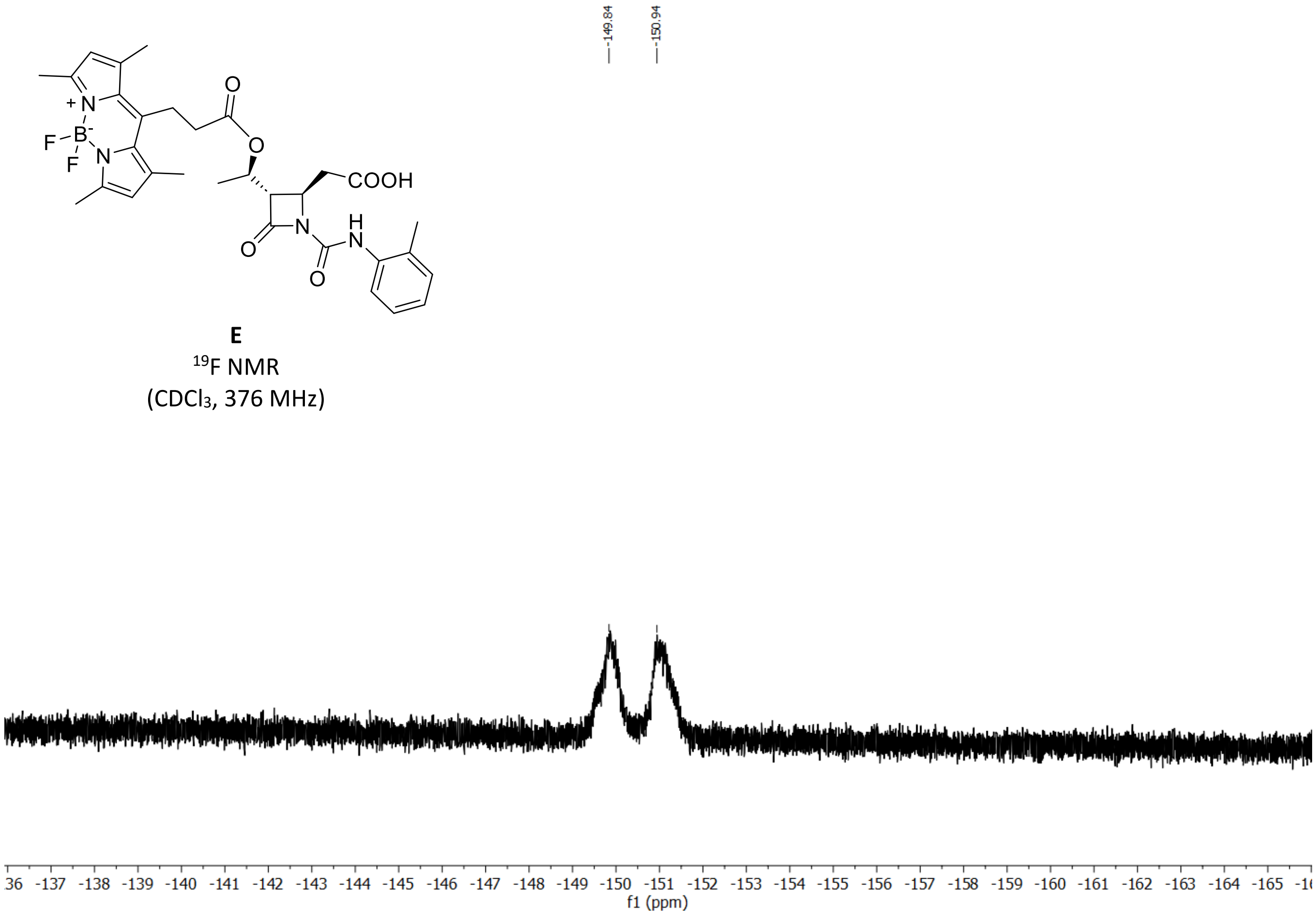

Figure S11. ^{1}H, ^{13}C and ^{19}F NMR spectra of compound **E.**

4 Concentration-response curves, obtained from cell adhesion assays performed in the presence of increasing concentrations of compounds D, E, 12, and 14

Cell adhesion assays. Concentration-response curves, obtained from cell adhesion assays performed in the presence of increasing concentrations of compounds **D**, **E**, **12**, and **14,** are shown in the following figures S2-S4.

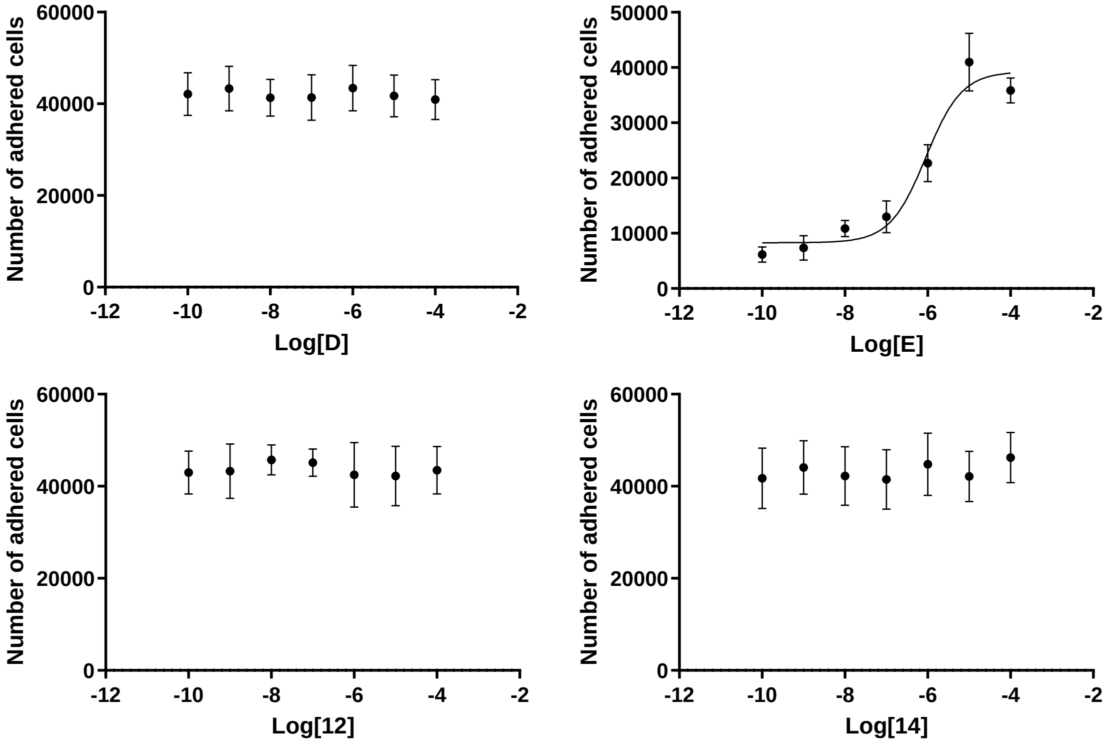

Figure S12 Concentration-response curves obtained from cell adhesion assays to evaluate the effects of the new theranostics **D**, **E**, **12**, and **14** on $\alpha_4\beta_1$ integrin-mediated Jurkat E6.1 cell adhesion to FN. Values represent the mean ± SD of three independent experiments carried out in quadruplicate.

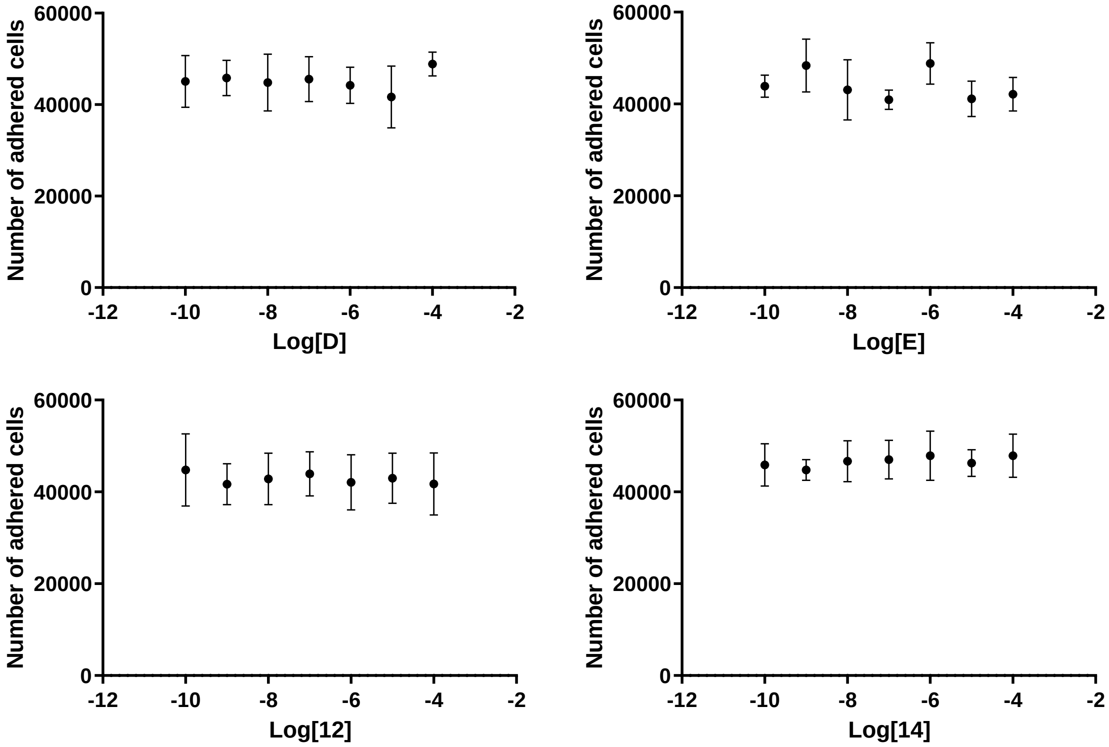

Figure S13 Concentration-response curves obtained from cell adhesion assays to evaluate the effects of the new theranostics **D**, **E**, **12**, and **14** on $\alpha_5\beta_1$ integrin-mediated K562 cell adhesion to FN. Values represent the mean ± SD of three independent experiments carried out in quadruplicate.

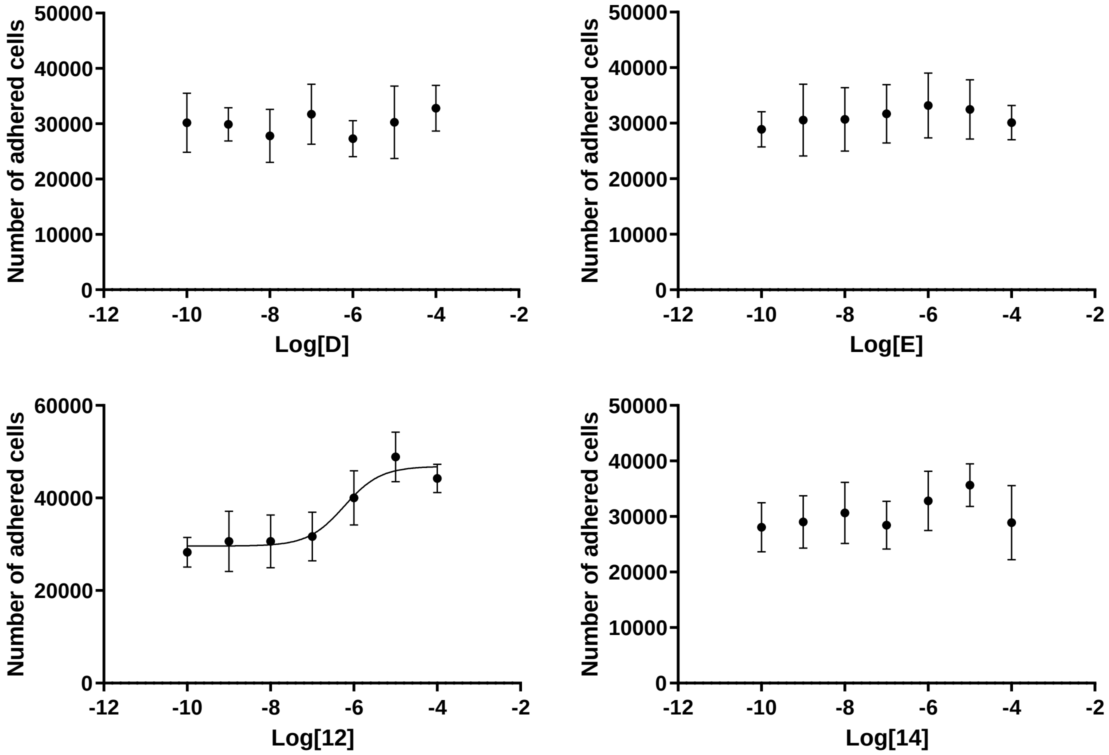

Figure S14 Concentration-response curves obtained from cell adhesion assays to evaluate the effects of the new theranostics **D**, **E**, **12**, and **14** on $\alpha_V\beta_6$ integrin-mediated HT-29 cell adhesion to FN. Values represent the mean ± SD of three independent experiments carried out in quadruplicate.

5 Cell viability assay in various cell lines treated with the theranostic compounds

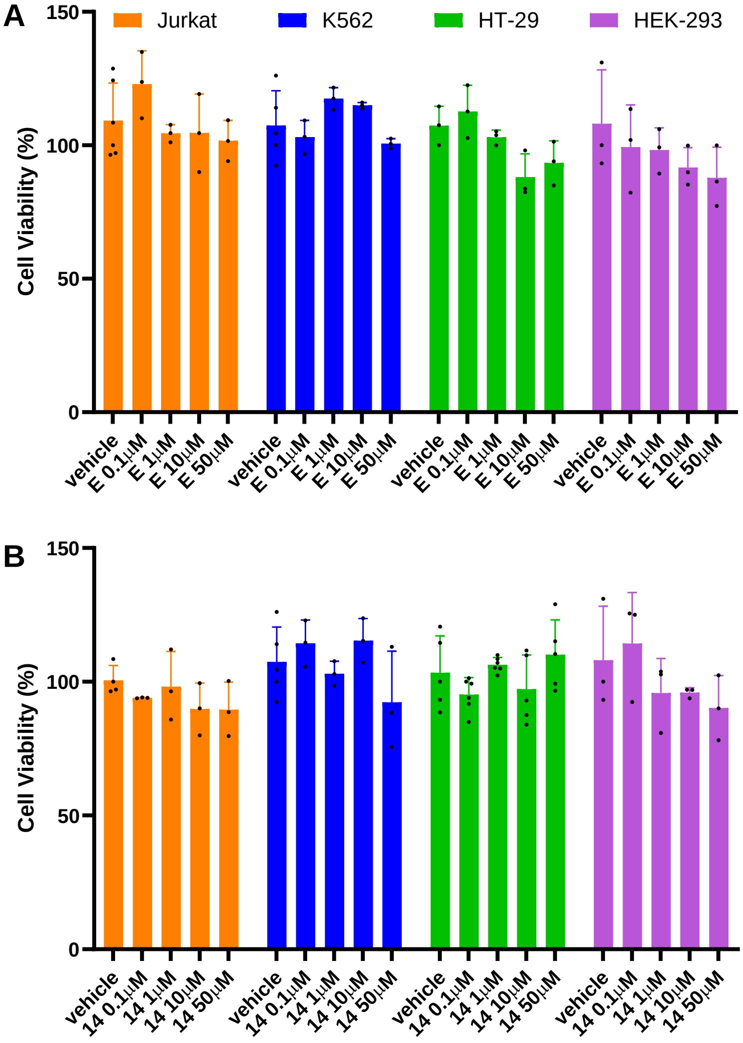

Figure S15. The theranostic compounds **E** and **14** were not able to affect cell viability without being activated by photosensitization. Jurkat E6.1, K562, HT-29, and HEK-293 cells were treated with different concentrations (0.1 – 50 µM) of **E** (**A**) or **14** (**B**) for 24 hours, and cell viability was assessed via MTT assay. Data shown represent the mean ± SD of three independent experiments, performed in triplicate. No statistically significant differences were observed (Dunnett's test after ANOVA).

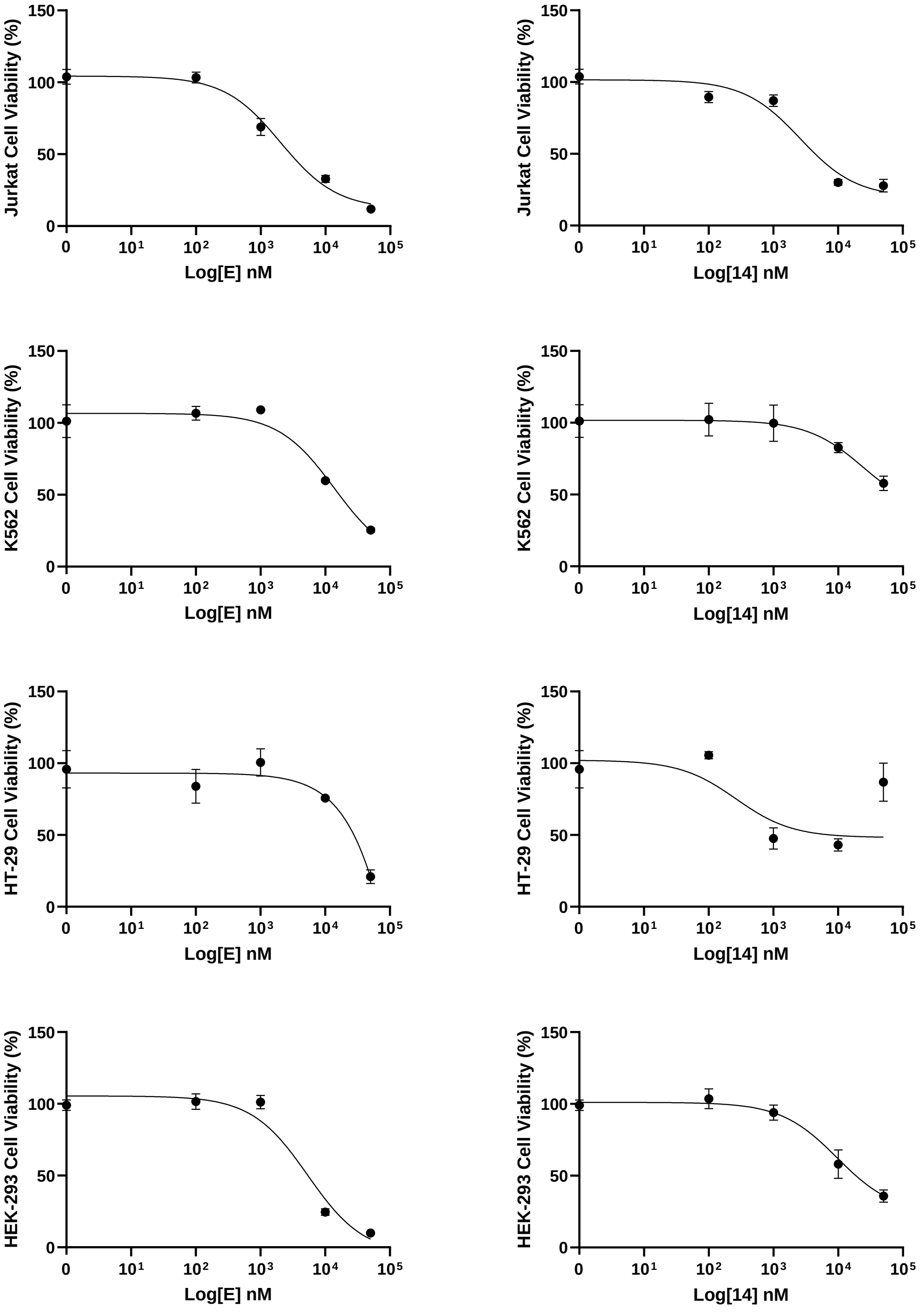

Figure S16. Concentration-response curves obtained from MTT assays to evaluate the potential cytotoxic effects of the new theranostics **E** and **14** on different cell models; those curves were used to derive the IC_{50} values reported in Table 2 (main text). Values represent the mean ± SD of three independent experiments carried out in quadruplicate.

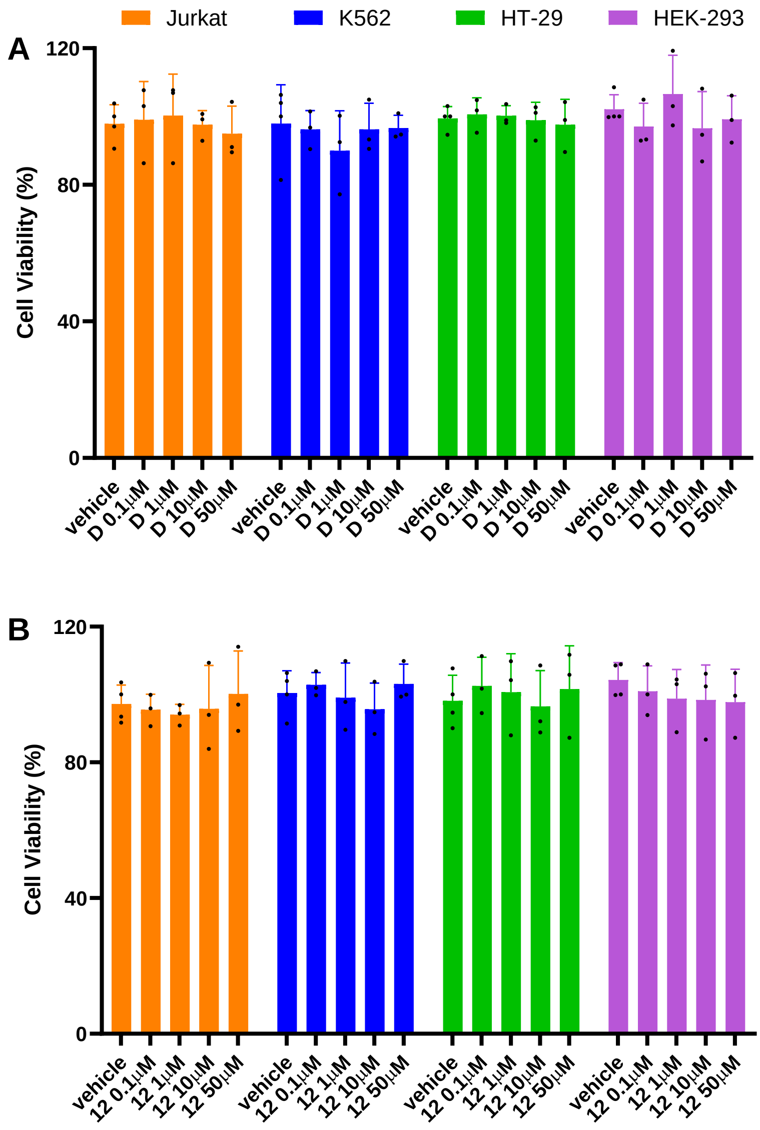

Figure S17. The theranostic compounds **D** and **12** were not able to affect cell viability in all cell models employed. Jurkat E6.1, K562, HT-29, and HEK-293 cells were treated with different concentrations (0.1 – 50 μM) of **D** (**A**) or **12** (**B**) for 24 hours, and cell viability was assessed via MTT assay. Data shown represent the mean ± SD of three independent experiments, performed in triplicate. No statistically significant differences were observed (Dunnett's test after ANOVA).

6. HPLC-MS spectra of target compounds D and E

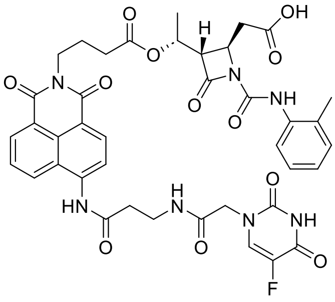

D

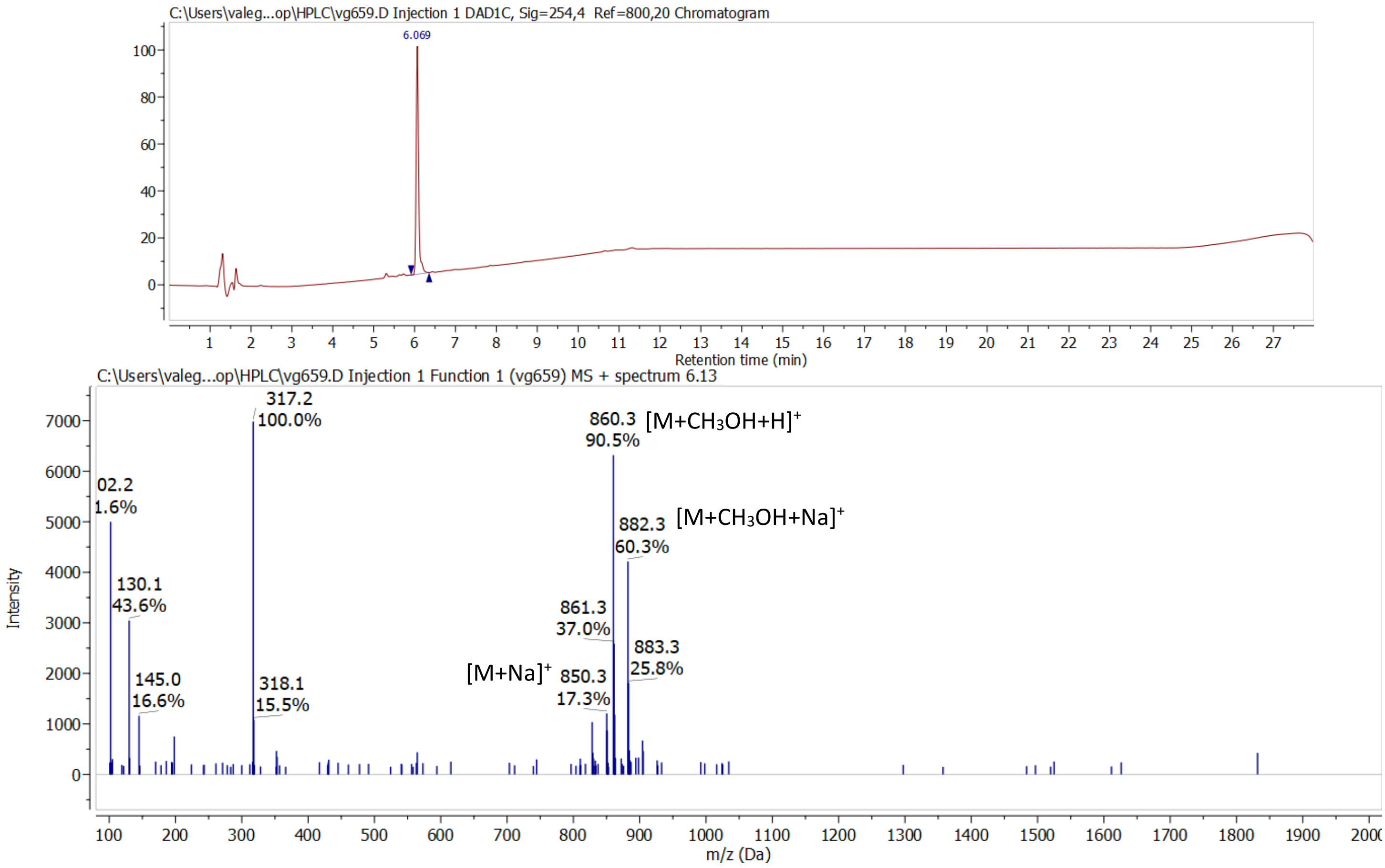

Figure S18. HPLC-MS spectra of compound **D**.

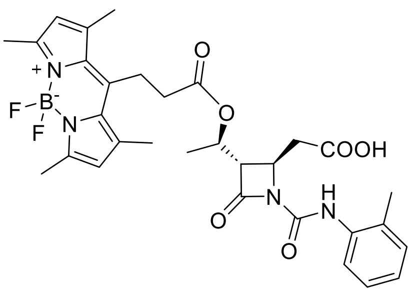

E

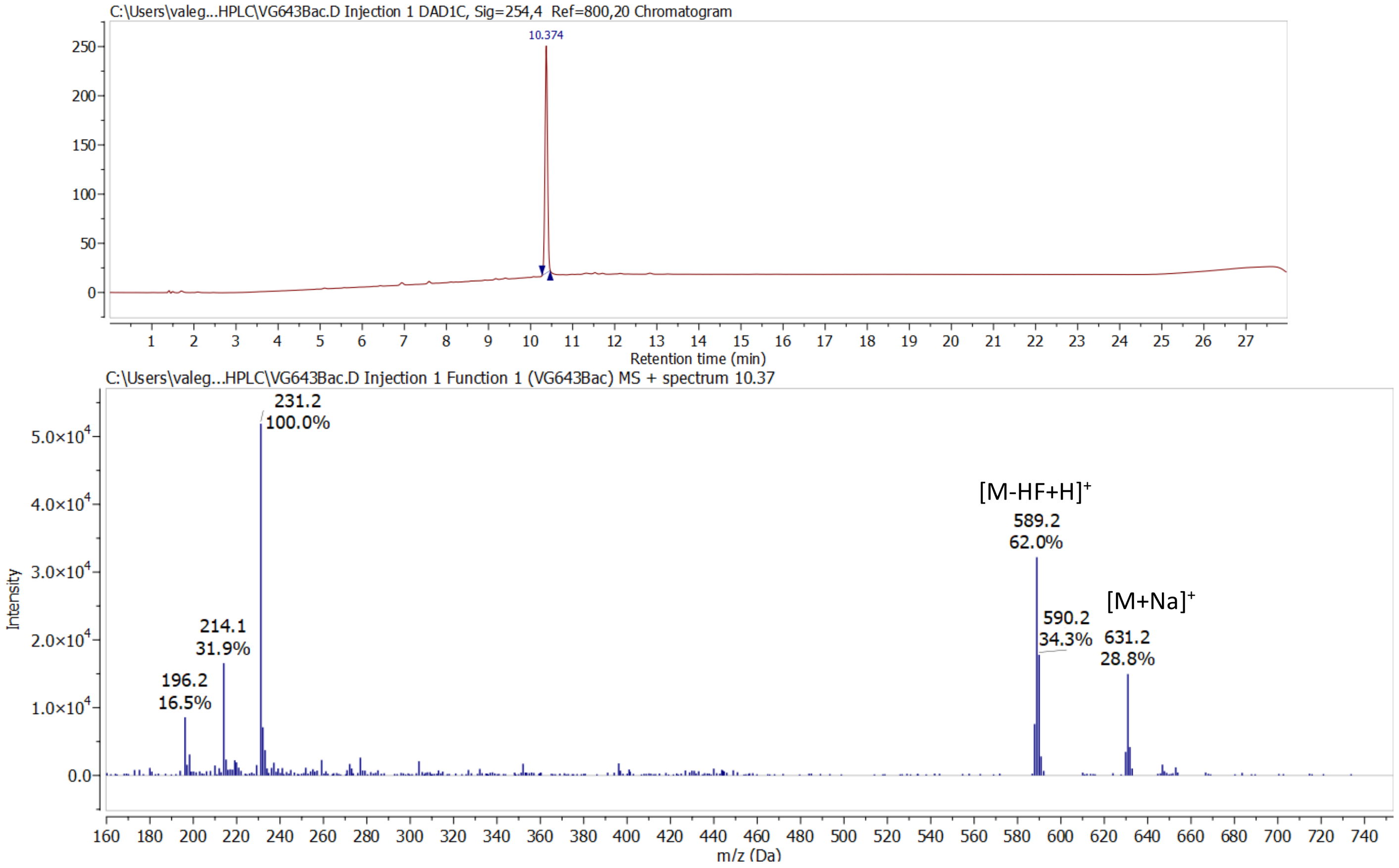

Figure S19. HPLC-MS spectra of compound **E.**
